# Modeling population size independent tissue epigenomes by ChIL‐seq with single thin sections

**DOI:** 10.15252/msb.202110323

**Published:** 2021-11-03

**Authors:** Kazumitsu Maehara, Kosuke Tomimatsu, Akihito Harada, Kaori Tanaka, Shoko Sato, Megumi Fukuoka, Seiji Okada, Tetsuya Handa, Hitoshi Kurumizaka, Noriko Saitoh, Hiroshi Kimura, Yasuyuki Ohkawa

**Affiliations:** ^1^ Division of Transcriptomics Medical Institute of Bioregulation Kyushu University Fukuoka Japan; ^2^ Laboratory of Chromatin Structure and Function Institute for Quantitative Biosciences The University of Tokyo Tokyo Japan; ^3^ Division of Cancer Biology The Cancer Institute of Japanese Foundation for Cancer Research Tokyo Japan; ^4^ Division of Pathophysiology Medical Institute of Bioregulation Kyushu University Fukuoka Japan; ^5^ Cell Biology Center Institute of Innovative Research Tokyo Institute of Technology Yokohama Japan

**Keywords:** dissociation‐free epigenome analysis, *in situ* epigenomics on single thin tissue section, tissue‐specific enhancers, transcriptional state decomposition, traveling ratio, Chromatin, Transcription & Genomics, Methods & Resources

## Abstract

Recent advances in genome‐wide technologies have enabled analyses using small cell numbers of even single cells. However, obtaining tissue epigenomes with cell‐type resolution from large organs and tissues still remains challenging, especially when the available material is limited. Here, we present a ChIL‐based approach for analyzing the diverse cellular dynamics at the tissue level using high‐depth epigenomic data. “ChIL for tissues” allows the analysis of a single tissue section and can reproducibly generate epigenomic profiles from several tissue types, based on the distribution of target epigenomic states, tissue morphology, and number of cells. The proposed method enabled the independent evaluation of changes in cell populations and gene activation in cells from regenerating skeletal muscle tissues, using a statistical model of RNA polymerase II distribution on gene loci. Thus, the integrative analyses performed using ChIL can elucidate *in vivo* cell‐type dynamics of tissues.

## Graphical abstract

“ChIL for tissues” allows obtaining epigenome information from a single‐thin tissue section with high sensitivity. A statistical model of RNApolII distribution is combined with ChIL to analyze cell‐type‐specific transcriptional states in a tissue.

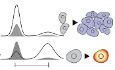

## Introduction

Tissues consist of terminally differentiated cells formed from stem cells, followed by cell‐type conversion and functional arrangement of cell types to the specified spatial localization. At present, the composition of the cells playing different functions and the mechanism by which each type is formed have been elucidated. This has allowed understanding the biological function of each tissue and the pathogenesis and developmental failure of diseases. Tissue composition can be determined using known cell‐type markers. Immunostaining for cell surface antigens and other cell‐type markers enables visual examination that determines the number and localization of cells and tissue morphology. Determining the cell types and the size of a cell population (i.e. number of cells) in tissues can be achieved by single‐cell (sc)RNA‐seq, which is based on the transcriptomic differences of individual cells (Schaum *et al*, 2018). Unsupervised clustering (Blondel *et al*, 2008) of the gene expression profiles allowed the identification of the cell types, including those previously unknown.

Epigenomic analyses are widely performed at the tissue level, such as in the Encyclopedia of DNA Elements (ENCODE), the National Institutes of Health Roadmap Epigenomics Project, and International Human Epigenome Consortium (IHEC) projects that utilize ChIP‐seq for bulk‐level tissues. Several studies have reported the comprehensive identification of functional elements in genomes, such as promoters, enhancers, and the binding sites of transcription factors and their regulatory relationships that characterize tissues (Shen *et al*, 2012; Roadmap Epigenomics Consortium *et al*, 2015; Stunnenberg & Hirst, 2016). However, in epigenomic analyses at tissue‐level, imbalance sampling cannot be avoided because tissues are mixtures of diverse cell types. Particularly, when the number of target cells (e.g., stem cells) is limited, they are masked by the information from the cells constituting the majority in the tissue. Furthermore, in ChIP‐seq, the genome coverage per cell is limited (Rotem *et al*, 2015); i.e., information on cells present in very small amounts in the bulk tissue is lost. Therefore, it is necessary to collect a large amount of target cells that meet the requirements of ChIP‐seq. As such, after defining the target cell types and markers, the sectioning of narrower area, dissection, or cell sorting is utilized. Recently, new methods have been developed for analyzing a small number of cells with higher genome coverage at the single‐cell level, including our ChIL method as well as others (Skene *et al*, 2018; Ai *et al*, 2019; Carter *et al*, 2019; Harada *et al*, 2019; Kaya‐Okur *et al*, 2019; Ku *et al*, 2019; Wang *et al*, 2019; Handa *et al*, 2020). In addition, isolating cells potentially affect the transcriptome of the cells owing to the physical dissociation of the tissues. Several tissue analysis methods that do not involve enzymatic digestion have also been proposed (Fanelli *et al*, 2010, 2011; Cejas *et al*, 2016; Amatori *et al*, 2018; Zhong *et al*, 2019; Font‐Tello *et al*, 2020). However, obtaining epigenomic information from a limited number of cells using ChIP‐seq‐based technology remains a challenge. To understand the biology of all cell types, the use of whole‐tissue analysis with single‐cell technologies is ideal, but very costly.

Several transcriptomic analysis approaches that combine the advantages of bulk RNA‐seq and scRNA‐seq, and can analyze and identify numerous cells at once, have been proposed. For example, the changes in the gene expression of cell types in bulk RNA‐seq profiles during embryogenesis have been interpreted using single‐cell RNA‐seq data collected separately (He *et al*, 2020). The estimation of the cell‐type composition of bulk tissue RNA‐seq based on single‐cell RNA‐seq has also been reported (Newman *et al*, 2019). Because data from different platforms complement each other, a data integration method has also been proposed, particularly the embedding of single‐cell RNA‐seq into seqFISH^+^ (Eng *et al*, 2019) data to virtually reconstruct whole gene expression data using spatial information (Stuart *et al*, 2019; Abdelaal *et al*, 2020). In addition, a computational approach for epigenomic analysis to decompose DNA methylation states into cell types has been suggested (Rahmani *et al*, 2019). However, to date, a universal solution for the cell‐type decomposition of tissue epigenomes has not yet been established.

Here, we propose a framework that integrates ChIL into the analysis of tissue slices and uses single, very small, and thin tissue sections. The obtained bulk tissue epigenome data showed dynamic changes in both the number and cell type, and computational modeling was thus required. We first optimized ChIL for highly sensitive genome‐wide analysis using a single thin section, as well as tissue visualization using immunostaining. ChIL is proposed to enable epigenomic analysis at the single‐cell level, and the acquired thin‐section ChIL data are expected to be the sum of the high‐depth single‐cell epigenomes. Using three different types of tissues, we confirmed the adequate sensitivity, specificity, and reproducibility of ChIL in identifying enhancers, transcription factors, and transcriptionally activated genes in whole tissues. Thus, we built a statistical model that evaluates the changes in the distribution of RNA Polymerase II at the gene loci and provides a robust, cell‐type resolution transcriptional regulatory analysis for large changes in population size.

## Results

### ChIL‐seq enabled spatial epigenomics with single tissue section

**Figure 1:**
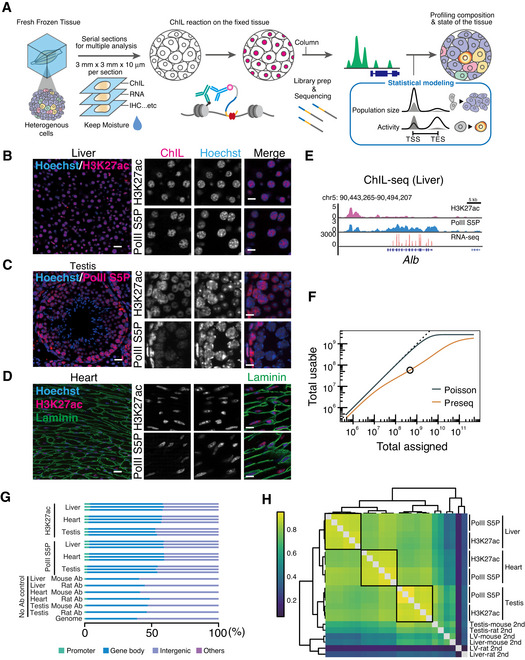
Epigenomic profiling using a single tissue section ASchematic diagram of the ChIL protocol for tissue.B–DImmunofluorescent images of mouse liver (B), testis (C) and heart (D). H3K27ac, PolII‐S5P, and laminin were stained with specific primary antibodies and visualized using fluorescent‐labeled anti‐mouse ChIL‐probe (red: H3K27ac and PolIIS5P) and anti‐rabbit IgG (green: laminin). DNA was counterstained with Hoechst 33342. Scale Bar: 20 µm (left images), 10 µm (right images).EGenome browser images of ChIL‐seq for H3K27ac and PolII‐S5P and bulk tissue RNA‐seq data at the *Alb* locus in liver tissues.FLibrary complexity of ChIL data. Poisson represents an ideal case of the uniform probability of obtaining reads from the mouse genome, whereas preseq refers to the future/past predictions of a species discovery curve of sequenced reads using Preseq (Daley & Smith, 2013). Black circle indicates the read number we sequenced for this prediction.GBreakdown of mapped reads at the annotated genomic regions. Gene body: 3’‐UTR, exon, intron, 5’‐UTR; Others: ncRNA, miRNA, snoRNA, and pseudogenes. The proportions of the annotated region on the mouse genome are shown as “Genome” at the bottom lane.HGenome‐wide correlation at 10 kbp bins. Hierarchical clustering of Pearson’s correlation coefficient of log‐transformed ChIL‐seq counts is shown.

Various cell types exist in tissues, each of which exhibits a unique localization pattern. The transcriptomic and epigenomic pattern of these cells may be affected by the enzymatic isolation process. Therefore, we focused on the use of tissue sections that are free from enzymatic treatment biases for epigenomic analysis and developed an experimental procedure using a single, very small, and thin tissue sections. We here optimized the ChIL for tissue **(**Fig 1A **)**, based on our previously reported sc‐epigenomic analysis tools (Harada *et al*, 2019; Handa *et al*, 2020).

Since the reports on analysis using microtissue sections are limited, and all of them require multiple tissue slices to obtain the required cell number (Fanelli *et al*, 2010, 2011; Cejas *et al*, 2016; Amatori *et al*, 2018; Zhong *et al*, 2019; Font‐Tello *et al*, 2020; Table 1). Low‐input and single‐cell‐level epigenomic analyses have been developed, such as CUT&Run (Skene & Henikoff, 2017) and MOW‐ChIP (Cao *et al*, 2015), and spatial (preprint: Deng *et al*, 2021) and single‐cell‐level (Bartosovic *et al*, 2021) epigenomic analyses have been applied to tissue analysis. However, the dissociation process to obtain a single cell requires a large number of starting cells. Therefore, it is currently difficult to analyze a small amount of starting cells such as those in human clinical samples. The dissociation process may cause a stress‐induced bias in gene expression of the tissue (Machado *et al*, 2021; Miyawaki‐Kuwakado *et al*, 2021). For this reason, epigenomic analysis using tissue sections has been widely used.

**Table 1:** Epigenomic analysis methods on tissue sections

Method	Tissue	Target	Sample Vol.	Author	Journal	Year
PAT‐ChIP	Spleen	Histone modification	10 μm ⨉ 4 sections	Fanelli *et al*	PNAS	2010
					Nat Protoc	2011
FiT‐seq	Seminoma	Histone modification	10 μm ⨉ 10 sections	Cejas *et al*	Nat Med	2016
	Breast cancer					
	Bladder cancer					
	CRC					
EPAT‐ChIP	Breast cancer	Histone modification	10 μm ⨉ 10 sections	Amatori *et al*	Clin Epigenetics	2018
Chrom‐EX PE	Liver	Histone modification Polymerase	20 μm ⨉ 2 sections	Zhong *et al*	BMC Genomics	2019
	Spleen					
FiTAc‐seq	Seminoma	Histone modification	10 μm ⨉ 2–4 sections	Font‐Tello *et al*	Nat Protoc	2020
	Breast cancer					
	Bladder cancer					
	Melanoma					
	PNETs					
ChIL for tissue	Liver	Histone modification Transcription factor Polymerase	10 μm ⨉ (3 mm ⨉ 3 mm) ⨉ 1 section	Maehara *et al*	This study	
	Heart					
	Testis					
	Skeletal muscle					
scCUT&Tag for tissue (dissociation, nucleus isolation, and microfluidic device)	Mouse brain	Histone modifications	150–250 K cells, or < 500 K nuclei extracted from a frozen section	Bartosovic *et al*	Nature Biotechnology	2021
hsrChST‐seq barcoding and CUT&Tag)	Mouse embryos (50 µm^2^)	Histone modifications	One section	Deng *et al*	BioRxiv	2021
	Brain region of an E11 mouse embryo (20 µm^2^)					
	Olfactory bulbs (20 µm^2^)					

We therefore focused on preparing frozen, unfixed tissue sections to equalize the fixation conditions. On plates, unfixed tissue thin sections are fixed with paraformaldehyde then permeabilized, followed by blocking. Immunostaining is then performed by reacting with primary antibodies against the target molecules on chromatin. Then, a fluorescent‐labeled ChIL‐probe attached with secondary antibodies was used to obtain the tissue localization of the target by imaging at the subcellular level. Subsequently, Tn5 transposase inserts an artificial sequence containing a T7 promoter into the genomic region near the target. *In vitro* transcription of the genome sequence near the target protein, starting from the T7 promoter, was performed, and the reverse‐transcribed DNA was sequenced using a next‐generation sequencer. Compared with conventional epigenomic analysis methods for FFPE and fresh‐frozen tissue slices, this method enabled uniform fixation conditions for the analysis of micro‐thin slices. Therefore, using the highly efficient ChIL method, we attempted to analyze tissues with an input size of 3 mm × 3 mm × 10 μm. Thus, we designed ChIL for tissue as a high‐precision method for analyzing the epigenetic information of a group of cells on a tissue section of the target, following the spatial distribution of the specific epigenetic status.

To evaluate the designed ChIL experimental procedure for tissue, the levels of the enhancer marker of histone modification H3K27ac and the recruitment of RNA Polymerase II (PolII), an indicator of transcription, were detected in three different tissues: liver, heart (left ventricle), and testis. Most of the cells were hepatocytes, comprising 70–80% of the liver. The H3K27ac signal visualized by the ChIL‐probe was uniformly distributed across cells on the sections. Subcellularly, the colocalization of H3K27ac and PolII in euchromatin regions (Hoechst‐negative) was observed (Fig 1B). In the testis, which consists of cells at multiple differentiation stages, the PolII signal was strongly distributed and localized in cells with high transcriptional activity, especially near the outer periphery of the seminiferous tubule (Sassone‐Corsi, 2002; Fig 1C), a region where cells in the early stages of sperm differentiation are located (Fig 1C). Meanwhile, the heart was co‐stained using laminin and the ChIL probe to distinguish the cell boundary regions and visualize the basement membrane (Fig 1D). S5P signal showed a localization to the low Hoechst‐dense region of the cell nucleus in which transcription may active, suggesting that immunostaining with ChIL probe was a valid histological staining method at the subcellular level (Fig 1B–D, Appendix Fig S1).

To validate the feasibility of ChIL for sensitive and accurate epigenomic analysis, we performed ChIL‐seq using a single thin section containing 1,000–10,000 cells (Table 2), which was generally assumed as a low number of cells in culture (Harada *et al*, 2019; Handa *et al*, 2020). The number of cells used was less than that of conventional epigenomic methods used especially for tissue analysis (Table 1). Furthermore, the genome‐wide analysis was performed by ChIL reaction on single sections that showed in Fig 1B–D. In the representative visualized epigenomic data in liver (Fig 1E), the accumulation of H3K27ac and PolII at the *Alb* locus, a hepatocyte marker, was observed. The former showed an activated upstream enhancer region, whereas the latter was highly transcriptional activity at the locus. The transcription of *Alb* was also confirmed using RNA‐seq with different serial slices. These results indicate that ChIL enables the simultaneous acquisition of both the tissue distribution of the epigenomic status and the genome‐wide epigenomic data using a single tissue section containing a small number of cells (10^3^–10^4^ cells in 10 μm^2^ area).

**Table 2:** Cell numbers in the tissue sections used in this study

Tissue section	Cell count (rep.#1–3)			Average
Heart H3K27ac	12,297	14,210	9,090	11,866
Heart PolII‐S5P	12,155	13,755	8,847	11,586
Liver H3K27ac	14,999	7,458	11,551	11,336
Liver PolII‐S5P	14,177	14,112	16,330	14,873
Testis H3K27ac	17,085	16,723	16,931	16,913
Testis PolII‐S5P	20,542	17,426	13,634	17,201
TA muscle PolII‐S5P	4,104	3,723	4,421	4,083

Next, to evaluate the genome‐wide distribution of the signals obtained using the ChIL procedure proposed above, we examined the specificity of the signal localization among different tissues and antibodies and the reproducibility of signal localization of the same tissue and antibody. First, to estimate the appropriate number of reads for ChIL‐seq with tissues, we obtained 480 M reads from PolII ChIL‐seq in muscle tissue and evaluated the library complexity (Daley & Smith, 2013) (i.e., the prediction curve of usable reads). As seen in Fig 1F, the number of total usable reads was starting to move away from the black line at approximately 10^7^, indicating a decreasing percentage of usable reads. Therefore, we determined that approximately 10^7^ reads is a good cost‐balanced number of the required reads in the case wherein the number of cells per section is < 10^4^. To obtain a ChIL signal with sufficiently high signal‐to‐noise ratio, we acquired an average of approximately 14 M reads (Dataset EV1), which is comparable to the number of reads in the ENCODE tissue ChIP‐seq (10 M–20 M) (Shen *et al*, 2012).

With this number of reads, the ChIL‐seq data from the liver, heart, and testis were obtained, and the genome‐wide localization of each data set is shown in Fig 1G. In all tissues and H3K27ac and PolII S5P antibodies, signals were concentrated around the coding regions (promoters and gene body) compared with the no‐antibody (herein, No Ab; without primary antibody) controls (53–59% and 41–48%, respectively). The PolII S5P antibody that we used detects both S5P alone and S5PS2P in the C‐terminal domain (CTD) of PolII (Odawara *et al*, 2011). PolII is known to switch from promoter‐proximal pausing state to elongation state by phosphorylation of Ser2 in addition to Ser5 in the C‐terminal domain (CTD) (Komarnitsky, 2000). In Appendix Fig S2, we compared the proportion of mapped reads in the same genomic region in Fig 1G and found that the S5P was a higher proportion than that in the S2PS5P in the gene promoters. These results suggest that ChIL with S5P and S5PS2P antibodies reflect the state of transcription in response to CTD phosphorylation of PolII. The results showed that the genomic sequences were selectively extracted from the transcriptionally activated regions of the genome. In Fig 1H, we describe the correlation matrix of the signal levels on the whole genome to confirm the high reproducibility of the replicates. The dendrogram shows the hierarchical structure of the highest correlation among the replicates (Liver‐H3K2ac: 0.90, Liver PolII: 0.90, Heart‐H3K27ac: 0.87, Heart PolII: 0.92, Testis‐H3K27ac: 0.91, Testis PolII: 0.94 in average of triplicates), and the correlation within the same tissue (e.g., Liver PolII vs. Liver‐H3K27ac: 0.87; Heart‐H3K27ac vs. Heart PolII: 0.88; and Testis‐H3K27ac vs. Testis PolII: 0.88; the list of all correlation coefficients are summarized in Dataset EV2). These results suggest that ChIL‐seq can capture the epigenomic differences between different tissues and is technically reproducible.

### Identification of regulatory factors in the formation of tissue‐specific enhancers

We next assessed the ability of ChIL for low‐input epigenomic analysis of tissues. First, we evaluated the agreement of the ChIL‐seq peaks with the reference ChIP‐seq peaks as we performed in previous reports (Harada *et al*, 2019; Handa *et al*, 2020). ChIP‐seq data from ENCODE Bing Ren’s data (Shen *et al*, 2012) were used as the gold standard reference for ChIP‐seq peaks in tissues (Appendix Fig S3A and B). Recall is coverage of the reference ChIP‐seq peaks, and precision is the proportion of true positives in query ChIL‐seq peaks. Recall was 58–60% for the heart, 62–64% for the liver, and 30–50% for the testis, and precision was 52–56% for the heart, 65–67% for the liver, and 36–46% for the testis. We also calculated the Jaccard index that is an integrated score of both recall and precision (heart: 38–40%; liver: 46–49%; testis: 20–29%). Although it was difficult to make a direct comparison of the values because of the different experimental conditions and cell types used, we compared the performance of ChIL‐seq with that of other methods for tissue epigenome analysis (Table 1). FiTAc‐seq (Font‐Tello *et al*, 2020) is an improved version of FiT‐seq for histone acetylation and is a state‐of‐the‐art method for the epigenomic analysis of FFPE tissue sections. We evaluated the concordance of FiTAc‐seq with ENCODE liver ChIP‐seq (Appendix Fig S3C). FiTAc‐seq showed very high concordance with ENCODE ChIP‐seq data (Recall: 70%, Precision 75%, AUC: 0.96–0.97), even though it was performed on FFPE. PAT‐ChIP of spleen H3K4me3, a similar tissue epigenomic analysis method, showed high agreement with ENCODE tissue ChIP‐seq data (AUC: ˜0.9; Appendix Fig S3D). Moreover, our ChIL‐seq obtained from a relatively small number of cells (one section; Table 1) was also highly comparable with FiTAc‐seq data for the same tissue and histone modifications (AUC: 0.94–0.95). Additionally, in accordance with the ENCODE standard for ATAC‐seq peaks (acceptable for FRiP > 0.2, recommended for > 0.3), the liver and heart were met these criteria. Conversely, the testis tended to have relatively low agreement with the reference ChIP‐seq peaks. This may be because of the more diverse composition of cells in testis tissue, especially compared with liver tissue that mostly consists of homogeneous hepatocytes.

**Figure 2:**
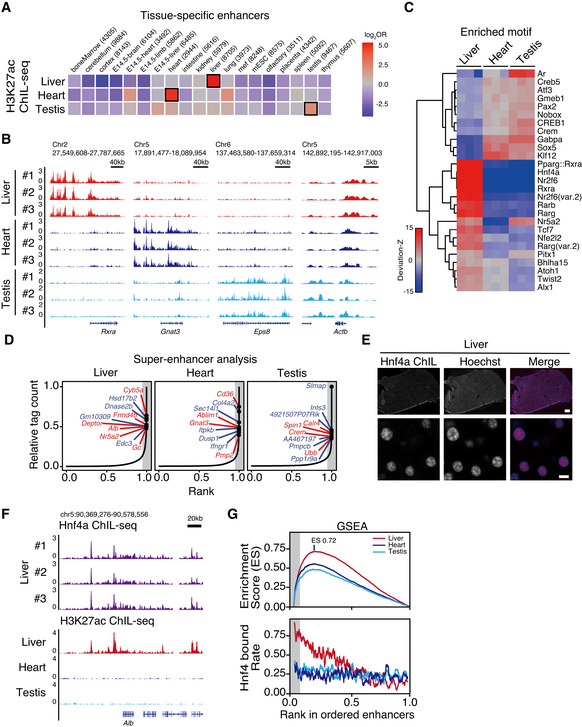
Upstream factor identification through enhancer analysis using ChIL‐seq Tissue specificity of identified enhancers by H3K27ac ChIL‐seq. The odds ratios of hits in the reference tissue‐specific enhancer list identified by bulk‐tissue ChIP‐seq data (Shen *et al*, 2012) are shown. Odds is defined in the Materials and Methods. The cells enclosed by black squares indicate the maximal odds ratios (i.e., maximal specificity) for each row.The IGV tracks of H3K27ac ChIL‐seq at identified tissue‐specific enhancers of *Rxra*, *Gnat3*, *Eps8*, and a house‐keeping gene of *Actb* loci are shown with the replicates.Specific motif enrichment analysis was conducted using chromVAR (Schep *et al*, 2017). Hierarchical clustering of deviation‐Z scores of three replicates of each tissue is shown.Super‐enhancer identification. Tissue‐specific enhancers are identified, so that they are listed more than twice (twice: blue, all: red) in the top 5% in all enhancer candidates and are not in the SEs of other tissues. Gray shades indicate the top 5% of tag count in enhancer candidates.Immunofluorescent images of mouse liver sections. Tissues were stained with anti‐Hnf4ɑ antibody and visualized by a fluorescent‐labeled anti‐mouse ChIL probe. DNA was counterstained with Hoechst 33342. Scale bar: 200 µm (top), 10 µm (bottom).Hnf4ɑ binds to the SE at *Alb* gene loci.Gene set enrichment analysis of Hnf4ɑ‐bound genes (top), and their rate of Hnf4ɑ‐bound genes in sliding windows of 100 genes (bottom).

Next, we performed ChIL using thinly sectioned tissues from the liver, heart, and testis, and the identified enhancers were compared by matching references (Shen *et al*, 2012; Fig 2A). According to the odds ratio (i.e., specificity, the detailed definition is described in Materials and Method), each H3K27ac ChIL‐seq signal preferentially captured the corresponding tissues‐specific enhancer (Liver: 33.5, Heart: 27.1, Testis: 4.1; the other odd ratios are listed in Dataset EV4). Therefore, we successfully detected tissue‐specific enhancers using ChIL‐seq with lower input compared to the previous reports that utilized 500 µg chromatin equivalent to 10^7^ − 10^8^ cells.

We also examined the enrichment of the H3K27ac signal on representative tissue‐specific enhancers, including the liver, heart, and testis. We focused on the enhancer region of *Rxra* genes (Joo *et al*, 2019) specifically expressed in liver tissues, *Gnat3* cardiac muscle‐specific gene retinoic acid receptor, and *Eps8* expressed in the blood–testis barrier (BTB; Lie *et al*, 2009). H3K27ac signal enrichments on each tissue‐specific enhancer were observed on the IGV screen shot (Fig 2B). In contrast, *all Actb*‐expressing tissues showed the ubiquitous enrichment of H3K27ac.

We further evaluated the enrichment of the regulatory sequence in extracted enhancers using ChIL based on the enrichment of the TF‐binding motif (only the top scoring motifs are shown in Fig 2C; the scores of the other motifs and the number of covered motifs by peaks are listed in Dataset EV5 and Dataset EV6). The enrichment of known liver‐specific TF‐binding motifs, Rxra, Hnf4a, Nr2f6, and others was observed in the H3K27ac ChIL‐seq data obtained from the liver. These data are consistent with the liver‐specific regulatory sequences registered as open chromatin regions detected using ATAC‐seq with mouse liver tissues in the database (Liu *et al*, 2019). Meanwhile, the H3K27ac signal obtained from the heart showed relatively higher enrichment at Klf12 than others; Sox5 and androgen receptor (AR) binding motifs were enriched in the testis‐H3K27ac signal, which was consistent with previous studies reporting that AR binds to the androgen responsible element (ARE) on regulatory sequences with histone acetyltransferase to regulate gene expression (Shang *et al*, 2002; Stelloo *et al*, 2018). These data support that H3K27ac ChIL‐seq can identify *cis*‐regulatory elements following the extraction of tissue‐specific enhancers.

Figure 2C shows the similarity between the testis and heart in the chromVAR analysis, and Fig 1H shows the similarity between the liver and heart. The difference appeared to be attributable to the chromVAR method of evaluating only signals around peaks. To test this hypothesis, we evaluated signal correlations of ChIL H3K27ac by restricting the signals on peaks (the union of the peaks in the three tissues; Appendix Fig S4A and B). As expected, the signals of the heart and testis showed similarity to the chromVAR results. This result was also reproduced using the genome‐wide 10 K bins with strong signals (count per million [CPM] > 20 in any tissue). In the liver data, for example, the bins satisfying CPM > 20 account for about 2% of the genome, but for nearly 20% of the total reads (Appendix Fig S4C). Therefore, the liver data show a high accumulation of signal in the peak region. In contrast, testis data showed that bins satisfying CPM > 20 accounted for less than 1% of all bins and contained 2–3% of total reads. The majority (98–99%) of the genomic regions with CPM ≤ 20 signals are considered to be the main source of genome‐wide correlation. This suggests that assessing the peak regions can help cope with the low S/N testis data. These results also suggest that we need to be aware of the difference between genome‐wide correlations, which include potential background signals, and signal correlations on peaks.

Because the enrichment of the ChIL‐seq signal should reflect the quantitative H3K27ac levels as demonstrated by the identification of super‐enhancers (SEs) using ChIP‐seq, we next quantitatively determined the H3K27ac level based on the read counts. Then, SE formation upon TF binding on the extracted cis‐regulatory elements was evaluated. First, we listed the highly enriched regions of the H3K27ac ChIL‐seq signal as SE(Lovén *et al*, 2013; Whyte *et al*, 2013) from each liver, heart, and testis data set. The labeled genes in Fig 2D are representative protein‐coding genes near the identified top ranked SEs, which have the highest read counts in peaks (see Appendix Fig S4D for all replicates). In the liver, known hepatocyte marker genes, *Alb*, and albumin family, *Gc,* are also detected in motif‐enrichment analysis performed in Fig 2C. In addition, the core transcription factor Hnf4α (Watt *et al*, 2003), which activates the genes by itself, was included in the top rank (1.6–3.5%). Furthermore, the SEs featuring each tissue were identified. In the heart (left ventricle), *Ablim1* expressed in the left ventricle and involved in left–right axis formation (Stevens *et al*, 2010) was detected, whereas in the testis, SEs were identified in the vicinity of *Crem*, which is involved in spermatogenesis (Blendy *et al*, 1996).

Finally, to validate the function of the SEs identified in the liver using this method, we performed ChIL targeting Hnf4α, which showed a high specificity score (deviation‐Z) in liver SEs. Hnf4α is known to be an important nuclear receptor during hepatocyte differentiation (DeLaForest *et al*, 2011) and has been shown to contribute to SE formation as a core transcription factor, along with RXRα (Joo *et al*, 2019). Immunostaining with the ChIL Probe showed that the HNF4α was distributed throughout most cells in the liver tissue and detected in the open chromatin region of the nucleus in each cell (Fig 2E). A pronounced accumulation of Hnf4α signals in the SEs in the region was observed (Fig 2F, see Appendix Fig S5A for the motif enrichment analysis on Hnf4α peaks). We next evaluated the selective binding of Hnf4α to the genes in the liver SEs (Fig 2G; Appendix Fig S5B and C for the replicates). Using the gene sets of SEs and typical enhancers neighboring genes obtained in Fig 2D, gene sets enrichment analysis (GSEA; Subramanian *et al*, 2005) demonstrated that the hits of the ChIL‐Hnf4α peaks against liver enhancers scored as high as 0.72 in the enrichment score (Fig 2G, top). Particularly, Hnf4α was bound to 76.4–78.8% of the SEs (Fig 2G bottom). In contrast, in the negative controls of the heart‐ and testis‐specific SEs, the number of SEs bound by Hnf4α was approximately 0.5 in the enrichment score and the percentage of Hnf4α bound to the heart‐ and testis‐specific SEs was at a random chance level (24.4–34.2%).

In summary, the data from ChIL H3K27ac demonstrated that the regulatory candidate transcription factor Hnf4α obtained from the *cis*‐element refinement selectively binds to the liver‐specific SE region of the *Hnf4a* locus. Hnf4α could be validated to provide positive feedback that binds to the SE region of its own *Hnf4a* locus. Our data indicated that ChIL is useful for the regulatory analysis of enhancers, including transcription factors and SEs, using low number of cells.

### PolIIChIL‐seq peaks detected the majority of active genes in tissue

**Figure 3:**
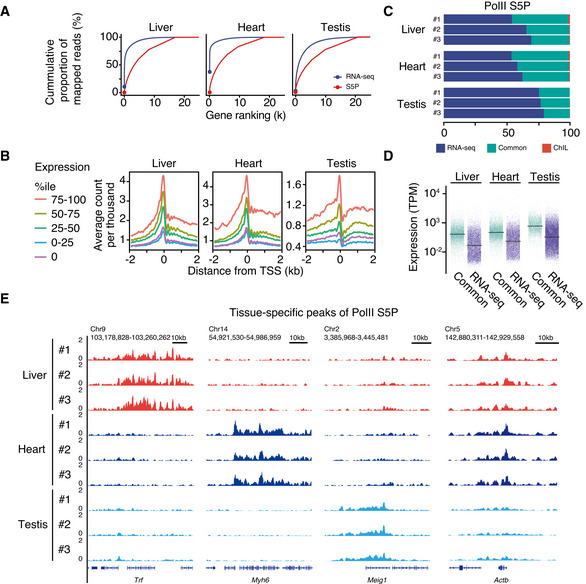
PolII ChIL‐seq detect active genes in tissue Dynamic ranges of bulk‐tissue RNA‐seq and PolII S5P ChIL‐seq. The cumulative proportion in total mapped reads at genes (red: ChIL‐seq, blue: RNA‐seq) were compared. Genes are ordered by the read counts on the exons for RNA‐seq and on ± 750 bp from TSS for ChIL‐seq, respectively.Signal intensities of ChIL‐seq correlated with the expression levels of genes. The lines indicate the average CPM of each expression group at TSS. The expression groups were assigned with respect to the expression levels (TPM) of genes.Coverage of expressed genes by PolII S5P ChIL‐seq peaks. The stacked bar chart shows the proportions of detected genes in the RNA‐seq only (RNA‐seq: blue), PolII S5P ChIL‐seq only (ChIL: red), and both (Common: green).Higher expression levels at PolII S5P ChIL‐seq peaks. The expression levels of all expressed genes (TPM > 0) are shown.The tissue‐specific genes identified by PolII S5P ChIL‐seq. The IGV tracks of all replicates of PolII S5P ChIL‐seq are shown at each specific gene (*Trf*, *Myh6*, and *Meig* for the liver, heart, and testis, respectively). *Actb* is also shown as the ubiquitously expressed gene in the three tissues.

Transcriptome information is obtained by evaluating the binding position of PolII using epigenomic analysis. Here, we detected the active genes based on the binding of PolII on the genome using ChIL. In Fig 3A, we plotted the cumulative number of consumed reads of the detected genes in RNA‐seq and PolII ChIL‐seq in the order of their read counts. Due to the wide dynamic range of RNA‐seq data, high copy‐number mitochondrial‐derived RNAs (e.g., mitochondrial ribosomal RNAs) and highly expressed genes that characterize each tissue (*Alb* in liver, *Myh6* in the heart, *Prm1* in testes), consumed 80% reads on a small fraction of highly expressed genes (Liver 5%, Heart 1%, Testis 11%). The identification of weakly expressed genes and rare populations in bulk tissue RNA‐seq is generally hard to obtain because the top 10% genes spends 80% of its reads in even at the single‐cell level (Liu *et al*, 2014; Van den Berge *et al*, 2019).

In contrast, ChIL‐PolII did not exhibit an exponential increase in the number of consumed reads required to detect gene expression from RNA‐seq. It also efficiently detected more genes as the number of reads increased. We further calculated the expected number of detected genes in the subsamples of reads (RNA‐seq and PolII S5P ChIL‐seq) based on Heck *et al* (1975). In the case of ChIL‐seq, a gene was considered detected if the read count was > 0 in the gene region ± 1 kb. As a result, the number of detected genes of both ChIL‐seq and RNA‐seq was close to each other at 1 × 10^4^–10^6^ reads, and thereafter in all tissues, showed higher numbers in PolII S5P ChIL‐seq (Appendix Fig S6A). These results indicate that PolII S5P ChIL‐seq detects more genes than RNA‐seq when the number of reads is more than 1 × 10^5^. The dynamic range of RNA‐seq depends on the product of the cell number and the concentration of RNA in each gene, whereas that of the PolII signals, in essence, depends on the product of the presence or absence of gene expression (0, 1, or 2) and the cell number. The results are consistent with the fact that highly and ubiquitously expressed genes occupy a high number of reads in the RNA‐seq data. The result suggested that fewer reads are required for gene expression profiling using PolII S5P ChIL‐seq than RNA‐seq.

Thus, the genes were divided into five groups based on their expression levels from RNA‐seq, and the correlation of each PolII S5P ChIL‐seq signal with their expression levels was examined (Fig 3B). In the high‐expression group in all tissues, the intensity of the PolII signal in the TSS was highly correlated with its expression level. In the 75^th^–100^th^ percentile group, a high accumulation of PolII in the gene body region was also detected, suggesting a movement of PolII to the locus upon transcriptional activation. Here, we showed that PolII S5P ChIL‐seq demonstrated a preference for capturing highly expressed genes in tissues. Subsequently, we assessed the overlap between RNA‐seq‐confirmed genes (TPM > 0) and PolII S5P ChIL‐seq peaks. The ChIL‐seq peaks captured approximately 30% (Testis slightly lower, approximately 20%) of the active genes (TPM > 0), whereas false positives were almost absent (Fig 3C). In addition, ChIL‐seq peaks stably detected approximately 40–50% of the genes expressed in RNA‐seq, independent of the TPM threshold for defining the expressed genes in RNA‐seq (Appendix Fig S6B). These results suggest that the peak region is likely to capture genes with high expression because the region with high signal counts was judged to be the peak region (Sun *et al*, 2011). In all tissues, the expression levels of the genes in Common were higher than those that in RNA‐seq group as expected (Fig 3D).

Figure 3E shows an IGV screenshot of the PolII S5P ChIL‐seq. The accumulation was detected at the *Trf* (transferrin) locus in the liver, *Myh6* (cardiac myosin) in the heart, and *Meig1* (a meiosis‐expressed gene) in the testes. These are considered representatives of genes specifically expressed in each tissue. At the *Actb* locus, a house‐keeping gene, the PolII signal was accumulated in all tissues, indicating active transcription. In these highly transcriptionally active genes, a wide distribution of PolII signals was detected on the gene body, suggesting that the PolII binding distribution patterns would enable an in‐depth profiling of the transcriptional programs in tissues.

### Modeling PolII traveling reveals transcriptional dynamics in the rapid change of cell population in skeletal muscle regeneration

We demonstrated that enhancers and transcriptional activity states can be detected with high sensitivity, specificity, and reproducibility at the whole‐tissue level by the optimized ChIL for tissues. Then, PolII S5P ChIL‐seq data in Fig 3 suggested that, in addition to amount of the signal at the gene loci, evaluation of the distribution or its elongation across the entire locus would improve the analysis of the transcriptional activation in various cells in tissue. We thus conceived a concept the statistical modeling of PolII S5P ChIL‐seq data for the epigenomic analysis of heterogeneous tissues.

**Figure 4:**
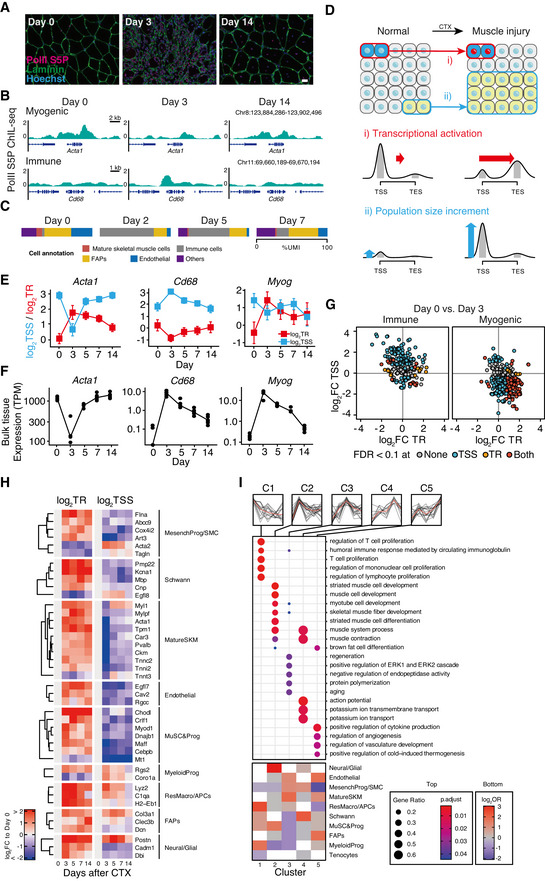
Statistical modeling of the traveling ratio reveals the independent dynamics between population and transcriptional regulation in regenerating skeletal muscle tissues Immunofluorescent images of the mouse tibialis anterior muscle on the indicated days after CTX treatment. The images of anti‐mouse ChIL probe for PolII‐S5P (red) and anti‐rabbit IgG for laminin (green) are shown. Scale bar: 20 µm. Refer to Appendix Fig S6A for more frequent time points.PolII S5P ChIL‐seq signal of the marker genes of mature skeletal muscle (*Acta1*) and macrophages (*Cd68*).Proportion of sequenced reads (%UMI) occupied by the representative cell types in muscle regeneration. The single‐cell data (GSE143437) by De Micheli *et al* (2020) was re‐analyzed. See Appendix Fig S6B for the detailed cell‐type annotations.Extraction of independent dynamics of the population and transcriptional regulation. Change in PolII distribution at the gene loci: a gene (blue) was transcriptionally activated (red nuclei) following the stimuli, while population size was unchanged. Change in the height of PolII distribution: a type of cells (yellow) was grown after the stimuli, while the transcriptional activity was maintained.Estimated mean (95% confidence interval) of TR and the CPM of PolII S5P ChIL‐seq at TSS. Representative genes of mature skeletal muscle cells and immune cells are shown.Bulk‐tissue expression levels (TPM) of the representative genes.Different activities of two major cell types in muscle regeneration. Scatter plots of log_2_FC of day 3 vs. day 0 of TR (x‐axis) and the TSS‐level (y‐axis) are shown: immune cell marker genes (left); myogenic genes: right. Colors indicate significance in TR and TSS levels based on |log_2_FC| > 1 (twofold) and FDR < 0.1.Activities of major cell types in muscle regeneration. The colors of the heatmap show the log_2_FC to day 0 (uninjured) of TR and TSS levels. Representative genes with significant changes in TR are shown.The dynamics of the biological process in muscle regeneration and the participating cell types. Genes were assigned to five groups (C1‐5) based on highest time point of TR. OR indicates the specificity of participation to the biological processes.

We used skeletal muscle regeneration as a model case, wherein numerous cell types dynamically change their composition, particularly that of the mouse tibialis anterior (TA) muscle after cardiotoxin (CTX)‐induced injury. During regeneration, migrating immune cells are dominated the tissue 2–3 days after muscle injury (Tidball, 2005). During this time, the activation of muscle satellite cells (MuSCs), which are responsible for skeletal muscle regeneration, leads to the regenerated muscle fibers observed on day 14. We thus established a model to analyze the gene expression dynamics in each cell type from day 0 (pre‐injured period) and until day 14. ChIL obtained data from five biological replicates using the tissue sections of TA muscles at five time points on days 0, 3, 5, 7, and 14 after the CTX‐induced muscle injury. As shown in Fig 4A (Appendix Fig S7A for the entire time‐course), the basal lamina separating the muscle fibers observed on day 0 was destroyed post‐injury. The destruction of the cells on the third day can be seen in the image of laminin co‐stained with the ChIL probe. Furthermore, the fluorescence image of the ChIL probe suggests the presence of multiple cell types, such as the activated MuSCs, muscle progenitor cells that have started to differentiate, and migrating immune cells associated with the inflammatory response. On day 14, the structure of the muscle fibers possessing central nuclei were observed, thus indicating regenerated muscles.

First, we visualized the distribution of the PolII signal by IGV for representative genes in skeletal muscle and immune cells. Changes in PolII distribution are observed at the locus for *Acta1* (which is highly expressed in skeletal muscle) and *Cd68* (a surface marker of macrophages; Fig 4B). The *Cd68* locus showed an overall increase in the PolII signal from day 0 to day 3, whereas *Acta1* showed an overall decrease. These results indicate the rapid increase in immune cells and the decrease in skeletal muscle cells during the early stages of injury (days 2–3) as shown in Fig 4C (Appendix Fig S7B for detail). In *Acta1*, however, the PolII signal is more concentrated near the transcriptional end site (TES) than the transcriptional start site (TSS). We thus hypothesized that the shape of the PolII distribution contains information on both the population size of cells and the regulatory state of a gene known as the pause/release of the PolII (Muse *et al*, 2007; Zeitlinger *et al*, 2007). Therefore, we established a model for two cases (or their combination) as shown in Fig 4D: one in which a specific gene of resident cells is activated by the induction of muscle regeneration (i), and the other in which the height of the already activated PolII signal increases due to an increase in the number of cells (e.g., migrated immune cells from outside the tissue) (ii). The traveling ratio (TR) is often used to evaluate the degree of PolII pause/release, as in Bartman *et al* (2019), providing a brief description of the geometry of the distribution of the PolII in the gene loci in terms of the ratio of the signal levels between TSS and TES. Furthermore, we modeled the estimation of TR as a form of Poisson regression with an offset term (see details in Materials and Methods). For each locus, the signal level CPM of PolII at the TSS is exp(*β_0_*), and that of TES is exp(*β_1_*) times the TSS level exp(*β_0_*), i.e., exp*(β_0_* + *β_1_)*. To explain the model intuitively, we further demonstrated our TR modeling with artificial data (Appendix Fig S7C–E). The model transformed a pair of (TSS and TES) count values at each locus into TSS and TR (TES/TSS). By fitting the PolII count data to this model, the TSS and TR provide the interpretation of the cell population size with an active gene and the degree of transcription activation of the genes. Furthermore, between‐sample normalization by employing the offset term of the total number of reads maintains the information of the sample size of count data (i.e., a large count is more reliable than small counts). The statistical model therefore allows us to evaluate the confidence intervals for TR and perform statistical tests for changes in varying conditions and time points.

Figure 4E shows the estimated values of the mean PolII levels at TSS and TR, along with the confidence intervals. We then compared the tissue‐wide expression levels of the corresponding genes (Fig 4F). Surprisingly, the tissues‐wide expression of *Acta1* and *Cd68* was synchronized with the pattern of the PolII TSS‐level, whereas the transcription factor myogenin (*Myog*) expressed in muscle progenitor cells at the differentiation stage has a synchronized pattern to TR. These results suggest that the tissue bulk RNA‐seq reflects a product of the cell number and the amount of gene expression.

Therefore, to distinguish the transcriptional activation indicated by the TR, and the population size indicated by the TSS‐level as inferred in Fig 4E and F, we analyzed the changes in the TSS‐levels and TR at day 3 (Fig 4G and Appendix Fig S7F). Each set of genes was associated with each “single” cell type, the definition of which is based on the scRNA‐seq analysis of injured muscle by De Micheli *et al* (2020). The population size of the cells that express the skeletal muscle‐related genes (Fig 4G, right) was decreased after injury, whereas the changes in TR revealed the active transcription of the genes. Meanwhile, in the group of genes associated with immune cells, TSS level was increased, while TR was less altered (Fig 4G, left), which can be interpreted as an increase in the population of cells already possessing active gene loci (i.e., migration). This interpretation is consistent with the dynamic population changes in muscle regeneration clearly revealed by recent studies using scRNA‐seq (Dell’Orso *et al*, 2019; De Micheli *et al*, 2020; Oprescu *et al*, 2020). In summary, the statistical model of PolII ChIL‐seq allowed us to evaluate the transcriptional activity of genes associated with specific cell types, independent of increased population of immune cells, and decreased skeletal muscle cells during muscle regeneration.

Next, we identified the uncharacterized dynamics in muscle regeneration from days 0–14 using the other cell‐type markers defined by De Micheli *et al* (2020). First, to assess changes in the number of cells in the tissue after muscle injury, we created a heatmap that showed the changes in the TSS level of all 83 genes used in this cell‐type annotation (Appendix Fig S7G). In particular, the number of cells in macrophages showed an increasing trend in response to inflammation, and myeloid progenitors and FAP showed a slightly elevated trend. Conversely, the numbers of cells in Schwann, mesenchymal progenitor/smooth muscle cells, MuSCs and progenitors, and mature SKMs were decreased from the preinjury level, whereas many genes showed an increasing TR trend. We next selected 66 genes among the markers that changed the TR (FDR < 0.1) at any time point compared with day 0. The changes in the TR and TSS level of these genes are shown as a heatmap (Fig 4H) to visualize the trends in the transcriptional activation of each gene, as well as the increase or decrease in the number of cells that harbor the activated genes. From the log_2_TSS, which indicates the cell number, we confirmed that mature skeletal muscles (SKMs) decreased once after injury (white to blue); however, most genes were activated at day 3 and returned to the original population size (white) at day 14. Many of the cell types, such as mesenchymal progenitors/SMCs, myeloid progenitors, and resident macrophages/APCs, transiently increased in number after injury but returned to their pre‐injured levels on day 14, indicating association with inflammatory responses (*Ada2, Rgs2, Coro1a, Lyz2, C1qa*; Naito *et al*, 2012; Oprescu *et al*, 2020). Meanwhile, *Myl1*, a gene that was transiently increased after injury, *Tnnc2*, and *Acta1*, showed the same TR pattern, suggesting that these genes also function in regeneration and not only in muscle fiber formation (Wang *et al*, 2015).

Next, we describe the muscle regeneration process by classifying gene groups according to the pattern of TR changes over time. The clusters C1‐5 were assigned according to their peaks (highest point) of TR in the time‐course of regeneration, the tissue‐wide dynamics were appeared in Fig 4I, suggesting transcriptional regulation in muscle regeneration. The C1 exhibits the highest TR at day 0 and thus indicates a down regulated biological process after the injury. The proliferation of the immune cell was repressed, and the major participants are the resident macrophages and APCs and myeloid progenitors. The C2, which has peak at day 3, districted the activation of myogenesis mainly orchestrated by MuSC, muscle progenitors and also by neural cells, which is consistent with previous reports (Wosczyna & Rando, 2018). The C3, which has peak day 5, does not show strong enrichment. The C4 contained muscle contraction, ion transport and action potential related GO terms, which suggests the regenerated muscle was formed at day 7. The C5 (day 14) showed the activation of angiogenesis in the late stage of regeneration (Latroche *et al*, 2017). Here, the statistical modeling that combined PolII‐mediated transcriptional elongation and population size changes achieved by our ChIL provides a strategy for understanding the process of muscle regeneration that is organized by diverse cell types in tissue.

The definition of TR used in this study (TES/TSS) does not consider the information from gene body (GB) regions (Appendix Fig S8A). We therefore evaluated the effect of changing the definition of TR. We first evaluated Pearson correlation coefficients between our TR and the TR that used GB (TR_GB_). Days 0, 3, 5, 7, and 14 were 0.39, 0.30, 0.32, 0.33, and 0.39, respectively (Appendix Fig S8B). Both TRs showed positive correlations, but TR_GB_ showed a rather mild change in TR, whereas our definition of TR (TES/TSS) showed a relatively pronounced change in TR on day 3 (Appendix Fig S8C). To verify this observation, we tested the TR changes of day 0 vs. day 3 after muscle injury. As a result, we found no significant (FDR < 0.1) changes in TR_GB_, except for *Dnajb1* (Appendix Fig S8D). This indicates that TR estimation using the TES region is favorable for statistical evaluation of changes in the transcriptional state.

The above validation shows that the TR statistical model can be used to analyze epigenomic state changes that are less affected by changes in cell population size in the tissue. Moreover, ChIL‐seq has the advantage that multiple epigenomic analyses can be performed on the same tissue using serial sections. As an application of this advantage, we evaluated the H3K27ac data using the same statistical model as used for TR (Appendix Fig S9A). In the case of H3K27ac, TR is not a measure of transcriptional elongation but an indicator of changes in the distribution of histone modifications at a locus, as defined by TES/TSS. As we show in the muscle injury Pol II data, an increased signal around the TSS with increased immune cells was observed, together with a decreased signal in the skeletal muscle and muscle progenitor cells. Next, we searched for genes in which histone acetylation is actively involved in transcriptional regulation (Stasevich *et al*, 2014) to relate the biological significance to the measure of distributional change (TES/TSS) at the locus. For each time point, we clustered each Pol II and H3K27ac data using the TES/TSS index estimated from five replicates (Appendix Fig S9B). We obtained four clusters with correlated signal distribution changes. By calculating the cross‐correlation between them (H3K27ac vs. PolII), we identified 3‐i as the largest cluster among those whose signal distribution changes were synchronized between H3K27ac and PolII. Cluster 3‐i contained many genes expressed in epithelial and skeletal muscle and skeletal muscle progenitor cells (Appendix Fig S9C and D). We also identified cluster 1‐ii, in which H3K27ac and PolII were synchronized but showed a different temporal pattern than cluster 3‐i. Cluster 1‐ii contained genes representative of immune cells. These results suggest the active involvement of chromatin in gene activation during muscle regeneration and demonstrate the advantages of ChIL‐seq for performing epigenomic analysis in the same tissue with multiple types of epigenomes.

### Application to transcriptional profiling in human cancer tissue

**Figure 5:**
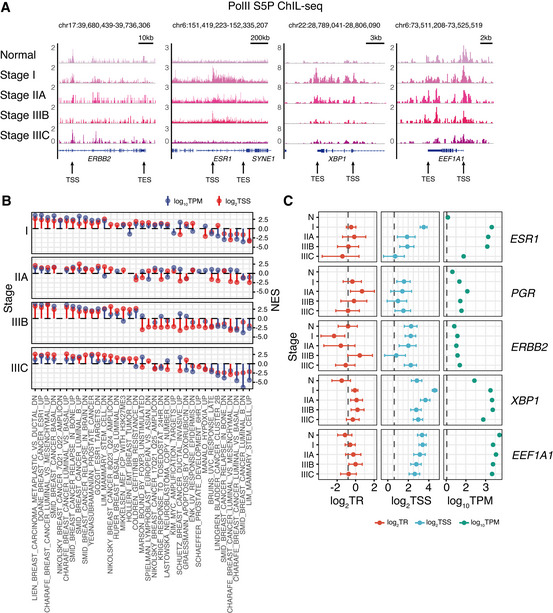
PolII ChIL dissects the transcriptional activity of genes in breast cancer tissue IGV screen shots of PolII S5P ChIL signals are shown for representative genes in breast cancer tissues. For each lane, individual samples were selected from each progressive stage of breast cancer.Gene set enrichment analysis of the CGP (chemical and genetic perturbations) gene set in MsigDB. The dots indicate the estimated NES using log_2_FC to the normal samples in TSS level and TPM. Terms that satisfy BH FDR < 0.1 are shown. P‐values were estimated by the adaptive multi‐level split Monte Carlo scheme implemented in the fgsea (Korotkevich *et al*, 2016) R package.Expression, TSS level, and TR of genes. *EEF1A1* is shown as a control housekeeping gene. The horizontal dotted line indicates the median level of all genes. Error bars indicate 95% confidence intervals for each estimate indicated by dots (*N* = 1 for each).

To demonstrate that ChIL is effective to analyze small amounts of tissue, we performed the analysis on human clinical samples in which the cell composition is diverse among individuals and a large number of cells is difficult to obtain. For each stage of cancer progression (I, IIA, IIIB, and IIIC) and one normal ductal sample, five samples were analyzed by PolII ChIL‐seq and RNA‐seq. In addition to well‐known markers for breast cancer (ER, PR, HER2), *XBP1*, which has been suggested to be upregulated in breast cancer tissues, is shown as a representative example of a significant change in TR (Fig 5A).

We first confirmed whether the characteristics of the gene expression in breast cancer were identified by PolII ChIL similarly to RNA‐seq. Gene set enrichment analysis was performed on the top 1,000‐fold changes to normal tissue in the TSS level and TPM. The CGP (chemical and genetic perturbations) gene set in MsigDB (Liberzon *et al*, 2011) was used and terms that satisfied Benjamini–Hochberg (BH) FDR < 0.1 are shown in Fig 5B. As a result, we detected substantial gene sets that are associated with breast cancer (e.g., BREAST_CANCER and BREAST_CARCINOMA). Additionally, TPM and TSS were in good agreement (92/117) for the same term with the direction of the normalized enrichment score (NES). These results suggest that PolII ChIL‐seq is effective for gene expression profiling in cancer tissues.

Both ChIL‐seq PolII and RNA‐seq indicated expression of ER, PR, and HER2, which suggested that all of them were HER2‐positive subtypes of breast cancer (Fig 5C). The marked expression of the ESR1 gene at stage I, which was comparable with that of *EEF1A1* (housekeeping gene), may be due to the majority of breast cancer tissues being composed of ER‐positive cells. Similarly, except for the decreased level at TSS in the stage IIIC sample, the high accumulation of PolII around the TSS of ER can be interpreted as ER‐positive cells are the majority in the tissues.

The sample from stage IIIB had relatively low accumulation of PolII in TSS at the *ERBB2* locus, but the gene expression level was comparable with other breast cancer tissues. In fact, the sample at stage IIIB was diagnosed to be occupied by a tumor at 30% in the frozen tissue block, which was relatively lower than the other cancer samples (refer to catalog from Origene). This result suggests the existence of a certain number of cells with higher transcriptional activity in the *ERBB2* locus of the tissue section at stage IIIB. Similarly, *XBP1* expression appeared to be maintained by high transcriptional activity, which promotes triple‐negative breast cancer (Chen *et al*, 2014) and is a candidate therapeutic target in high Myc‐expressing breast cancer (Zhao *et al*, 2018). These results demonstrate the potential application of epigenomic analysis of pathological samples by ChIL, such as evaluating the malignancy of tissue.

## Discussion

Here, we established a high‐precision method for tissue epigenomic analysis using single, thin section samples. We proposed a method for elaborate transcriptional profiling that reflects diverse and unknown cellular compositions in tissues by integrating the statistical model of the traveling ratio with highly sensitive epigenomic analysis of ChIL‐seq. RNA‐seq can only provide information on an increase or decrease of the total amount of RNA in tissue sections. Conversely, our method provides two types of information simultaneously: the increase or decrease of the cell population in the tissue and the transcriptional activation of genes at high sensitivity.

In this analysis, we utilized single‐cell analysis transcriptomic data as a reference of cell‐type annotation. The efficient combination of existing single‐cell analysis data and bulk but high‐depth ChIL‐seq data may lead to future approaches to analyze large numbers of individuals at the whole‐cell level.

We demonstrated that the transcriptional regulation of each cell type can be analyzed independently, even in situations with large‐scale variations in tissue cell‐type composition, as in the case of muscle regeneration. PolII ChIL‐seq by itself can also provide a qualitative assessment of the changes in cell population size. Although we did not identify the cell types in the tissues nor estimated their compositional ratios, our framework that combined scRNA‐seq and epigenomic analysis provides solid guidance for future tissue analysis. Using time series data from a skeletal muscle injury model, we demonstrated that our methodology is useful to assess the heterogeneous population size and transcriptional activity of cell types in tissues. We could also associate marker genes with a particular cell type. As is widely conducted in the field of gene expression analysis, ChIL‐seq enables comprehensive screening of transcriptional state changes of all genes. Furthermore, in the analysis of breast cancer tissues, we showed the potential application of detecting transcriptional activity of a minor cell population, which tends to be unclear in RNA‐seq.

The traveling ratio (or pausing index), a concise measure of RNA polymerase II dynamics, which was originally introduced in the ChIP‐chip as a measure of the degree of transcriptional elongation (Muse *et al*, 2007; Zeitlinger *et al*, 2007); and used in GRO‐seq (Core *et al*, 2008) and ChIP‐seq (Rahl *et al*, 2010). We found that the shape of the distribution of PolII at the genomic locus, as revealed by epigenomic analysis, is indeed a useful indicator of the transcriptional activity of a gene, and that the RNA‐seq of bulk tissue is the sum of all transcripts of all cells and is always affected by the population size. TR was previously evaluated by the ratio of the signal around the gene body to the promoter. However, we found that using the TES/TSS ratio, which has the same definition as the analysis of Bartman *et al* (2019), is more sensitive for the statistical test of the traveling ratio.

The statistical modeling of TR provides analogous advantages in the analysis of differentially expressed genes, such as the screening of genes with altered transcriptional states and calculation of confidence intervals for TR. Here, we used a simplified model in which the PolII signal at a single locus is the product of the size of the active population and the degree of activity (traveling ratio). Alternatively, a more realistic model with different transcriptional activities for different cell types and within the same cell type may be possible as proposed in the bulk data decomposition methods (Newman *et al*, 2019; Rahmani *et al*, 2019; Zaitsev *et al*, 2019). Despite our simplified assumption, our established model successfully determined transcriptional activities by cell type within a tissue. In addition, PolII ChIL‐seq data can be modeled using a simple Poisson distribution rather than a negative binomial distribution, which involves a complex dispersion parameter estimation. Furthermore, the use of CPM normalization with offset terms as a natural way of handling replicates made the model easier to apply, interpret, and use for tissue epigenome profiling. Our proposed statistical modeling of the traveling ratio can be carried out using a generalized linear model with an offset term in any statistics software. It requires input data of read counts for TSS and TES and the total number of reads and does not require any specialized analysis software. An implementation example in R is shown in our Github page.

Conventional ChIP‐seq has a limited genome coverage of cell owing to the efficiency of immunoprecipitation. In contrast, the original version of ChIL‐seq achieves a higher genome coverage of at least 90% for histone modifications at the single‐cell level. Accordingly, the acquired data were assumed to be a sum of the deeply profiled cells. Thus, we believe that the acquisition of such high‐depth epigenome data will continue to be necessary for the modeling compositions of tissues as shown in our framework. These high‐depth data are expected to be provided not only by ChIL‐seq, but also by other single‐cell epigenomic analysis methods; thus, other methods can be integrated to our analysis framework.

ChIL‐seq showed great potential to replace ChIP‐seq, which has been the standard method of epigenomic analysis for tissues. In this paper, the high reproducibility of ChIL for tissue, both technically and biologically, was demonstrated. Furthermore, ChIL‐seq achieved comparable performance while using fewer cells than ChIP‐seq (˜1/10,000 of required cell), and parameters, such as fixation conditions, can be monitored based on the quality of immunostaining images. These advantages can reduce cost. In addition, by combining visualization and genome‐wide analysis the spatial characteristic can be profiled and linked with the genome‐wide characteristics of epigenomes as shown in the massive wave of PolII in the testis. For more advanced applications, by leveraging the pairing of serial thin sections of the same mouse, the correlation between spatial and genome‐wide patterns of heterologous proteins, such as histone modifications and transcription factors, may be reliably estimated. We believe our proposed method is a useful tool for tissue epigenomic analysis, together with recent scRNA‐seq and microscopy‐based spatial transcriptomics.

## Materials and Methods

### Reagents and Tools table

**Table:** 

Reagent/Resource	Reference or Source	Identifier or Catalog number
**Experimental Models**		
C57BL/6N (*M. musculus*)	Japan SLC, Inc.	C57BL/6NCrSlc
Frozen OCT‐embedded Tissue Block (cancer stage: normal) (*H. sapiens*)	OriGene	#CB509696
Frozen OCT‐embedded Tissue Block (cancer stage: stageIIA) (*H. sapiens*)	OriGene	#CB648009
Frozen OCT‐embedded Tissue Block (cancer stage: stageIIIB) (*H. sapiens*)	OriGene	#CB615985
Frozen OCT‐embedded Tissue Block (cancer stage: stageIIIC) (*H. sapiens*)	OriGene	#CB552569
Frozen OCT‐embedded Tissue Block (cancer stage: stageI) (*H. sapiens*)	OriGene	#CB651675
**Antibodies**		
mouse anti‐H3K27ac	Kimura H *et al* (2008)	9E2H10, RRID:AB_2819244
mouse anti‐Rpb1 S2P CTD (S2PS5P)	Stasevich TJ *et al* (2014)	PC26, RRID:AB_2819246
rat anti‐RNA polymerase II S5P	Odawara *et al* (2011)	1H4B6
rabbit anti‐Laminin	Sigma‐Ardrich	#L9393
rabbit anti‐HNf4α	Cell Signaling Technology	#3113, RRID:AB_2295208,C11F12
**Oligonucleotides and sequence‐based reagents**		
MEDS‐B		
5' ‐ GTCTCGTGGGCTCGGAGATGTGTATAAGAGACAG ‐ 3'	Harada A *et al* (2019)	
5' Ph ‐ CTGTCTCTTATACACATCT ‐ 3'		
* annealed at 10 μM, as described in ChIL DNA, stored at 4°C		
Read2 primer (12 μM)		
5’‐ GTCTCGTGGGCTCGGAGATGTGTATAAGAGACAGNNNNNNNNNNN −3’	Harada A *et al* (2019), Handa T *et al* (2020)	
UDI primers (ChIL custom primers for PCR amplification, 10 μM)		
Fw: AATGATACGGCGACCACCGAGATCTACAC‐8 bp index‐TCGTCGGCAGCGTCAGATGTG	Harada A *et al* (2019), Handa T *et al* (2020)	
Rv: CAAGCAGAAGACGGCATACGAGAT‐8 bp index‐GTCTCGTGGGCTCGGAGATGT		
**Chemicals, enzymes and other reagents**		
PBS	Nacalai Tesque	#27575‐31
Cardiotoxin	LATOXAN	#L8102
16% Paraformaldehyde	Electron Microscopy Sciences	#15710‐S
ibidi µ‐Plate 96‐well TC (ibiTreat)	ibidi	#b89626
MAS coated micro cover glass	Matsunami	MAS coated cover glass(0.13‐0.17 mm), special ordered, used as slide glass
ULTRA PAP PEN S	Bio Medical Science	#BC‐PAPPEN‐S
TritonX‐100	Roche	#11332481001
VECTOR M.O.M. Immunodetection Kit	Vector Laboratories	#BMK‐2202
Blocking ONE	Nacalai Tesque	#03953
Isopentane	Nacalai Tesque	#26404‐75
ChIL probe (mouse, rat, rabbit)	Harada A *et al* (2019), Handa T *et al* (2020)	0.5 mg/ml
2× Tn5 dialysis buffer	Harada A *et al* (2019), Handa T *et al* (2020)	100 mM HEPES‐KOH (pH7.2), 200 mM NaCl, 0.2 mM EDTA, 20% Glycerol, 0.2% TritonX‐100, 2 mM DTT
Tn5	Picelli *et al* (2014) (in‐house prepared)	0.833 mg/ml
5× TAPS‐DMF	Harada A *et al* (2019), Handa T *et al* (2020)	50 mM TAPS‐NaOH (pH8.5), 25 mM MgCl_2_, 50% DMF (N,N‐dimethylformamide)
T4 DNA Ligase	NEB	#M0202L
T4 DNA Polymerase	NEB	#M0203L
Thermo T7 RNA Polymerase	TOYOBO	#TRL‐252
ATP, GTP, CTP, UTP (100 mM)	Thermo Scientific	#R0441, #R0451, #R0461, #R0471
Recombinant RNase Inhibitor	TaKaRa	#2313A
Recombinant DNase I (RNase‐free)	TaKaRa	#2270A
RNaseA	Nacalai Tesque	#30100‐31
SMARTScribe Reverse Transcriptase	TaKaRa	#639538
10× Lysis buffer	TaKaRa	#635013
Advantage® UltraPure PCR Deoxynucleotide Mix (10 mM each dNTP)	TaKaRa	#639125
SeqAmp DNA Polymerase	TaKaRa	#638504
AMPure XP	BECKMAN	#A63881
RNAClean XP	BECKMAN	#A66514
Elution buffer (EB)	Qiagen	#19086
Hoechst 33342 solution (1 mg/ml)	Nacalai Tesque	#19172‐51
DNA Clean & Concentrator‐5	ZYMO	#D4014
SMART‐Seq® Stranded Kit	TaKaRa	#634444
DNA Clean & Concentrator Kit (DCC‐5)	ZYMO RESEARCH	#D4014
Direct‐Zol RNA kit	ZYMO RESEARCH	#R2063
**Software**		
trim galore (version 0.6.2)	https://www.bioinformatics.babraham.ac.uk/projects/trim_galore/	
salmon (version 1.5.1)	Patro R *et al* (2017)	
bowtie2 (version 2.3.5.1)	Langmead and Salzberg (2012)	
samtools (verison 1.9)	Li H *et al* (2009)	
deeptools (version 3.4.1)	Ramírez F *et al* (2016)	
preseq (version 2.0.3)	Daley T and Smith AD (2013)	
chromVAR (version 1.14.0)	Schep A N (2017)	
MACS2 (version 2.2.7.1)	Zhang Y *et al* (2008)	
agplus (version 1.0)	Maehara K and Ohkawa Y (2015)	
**Other**		
Illumina HiSeq 1500	Illumina	
Illumina NovaSeq 6000	Illumina	
KEYENCE BZ‐X710	KEYENCE	

### Methods and Protocols

#### Ethical statement

All animal procedures were conducted in accordance with the Guidelines for the Care and Use of Laboratory Animals and were approved by the Institutional Animal Care and Use Committee (IACUC) at Kyushu University. The study protocol was approved (number: 579‐04) by the institutional review board for clinical research (IRB) of Kyushu University.

#### Tissue preparation

Eight‐week‐old C57BL/6N mice were used as replicates for this study. The liver, left ventricle, and testis were prepared from male, and tibialis anterior (TA) muscles were from female mice. Tissues were freshly frozen using isopentane chilled with LN2 and stored at −80°C. Muscle regeneration studies were performed as previously reported, except for the injection of CTX into the TA muscle (Ono *et al*, 2011). Injured and intact TA muscles were sampled from five mice at day 0, 3, 5, 7, and 14 after CTX injury. The day 0 indicates a needle‐injured control. The frozen tissue blocks of breast cancer tissue were purchased from OriGene.

#### Tissue ChIL experiments

Modifications to the original ChIL protocol for tissue analysis are underlined.

Tips) Maintain the humidity of tissue sections during the experiment.

Day 1: Tissue section preparation. Dissect tissue and snap freeze using LN_2_‐chilled isopentane without fixation. Store the tissue in a − 80°C freezer until use. Cryosection the tissue at 10‐μm thicknesses. Mount the sections on an Ibidi 96‐well plate (ibidi #b89626) or glass slide (Matsunami, MAS coated 0.13–0.17 mm thickness, special ordered). Pap pens (BMS #BC‐PAPPEN‐S) were used to segregate reagents on a glass slide to avoid cross‐contamination between mounted tissues and hold reagents.

Day 2: Immunostaining. Wash the section quickly with a sufficient volume of PBS (Nacalai Tesque #27575‐31). After removing the PBS, add 100 μl of 4% paraformaldehyde (Electron Microscopy Sciences #15710‐S) and 0.3% Triton X‐100 (Roche #11332481001) in PBS. Incubate at RT for 5 min. After removing the paraformaldehyde, wash the cells with 200 μl of 0.3% Triton X‐100 in PBS twice. After removing the PBS, add 100 μl of 0.3% Triton X‐100/PBS. Incubate at RT for 5 min. After removing the Triton X‐100, wash the cells with 200 μl PBS twice. After removing the PBS, add 100 μl of Blocking One (Nacalai Tesque #03953). Incubate at RT for 20 min. For mouse/rat antibody‐stained sections, additional blocking is performed by incubating with 100 µl M.O.M. IgG (Vector Laboratories #BMK‐2202) in PBS for 1 h. After removing the blocking reagent, wash the section with 200 μl PBS. After removing the PBS, add 100 μl primary antibody (1 μg/ml) in M.O.M. protein concentrate/PBS. Incubate at 4°C overnight. Antibodies used in this study are listed in the Reagents and tools table.

Day 3: ChIL probe staining. After removing the primary antibody, wash the cells with 200 μl PBS three times. After removing the PBS, add 100 μl ChIL probe (5 μg/ml) in ice‐cold MOM protein concentrate and 0.5 M NaCl in PBS. Incubate at 4°C overnight.

Day 4: ChIL reaction for library construction. After removing the ChIL probe, wash the cells with 200 μl PBS three times. Nuclear counterstaining is performed with Hoechst® 33342, followed by imaging all sections under a KEYENCE BZ‐X710. After removing the PBS, prebuffer sections with 100 μl of 1× Tn5 dialysis buffer and add 100 μl in‐house Tn5 (0.2 μl/well) in 1× Tn5 dialysis buffer. Incubate at RT for 1 h. Remove Tn5 and add 100 μl of MEDS‐B (10 μM, 0.2 μl/well) in 1× Tn5 dialysis buffer. Incubate at RT up to 1 h. After removing the solution, wash the sections with 200 μl PBS. After removing the PBS, wash the sections with 100 μl of 1× Tn5 dialysis buffer. After removing the buffer, add 100 μl of 1× TAPS‐DMF buffer. Incubate at 37°C for 1 h. After removing the buffer, add 100 μl of 0.2% SDS. Incubate at RT for 10 min. After removing the SDS, wash the cells with 200 μl PBS three times. After removing the PBS, wash the sections with 100 μl of 1× T4 DNA Ligase buffer. Prepare a fill‐in solution as follows: nuclease‐free water (88.5 μl), 10× T4 DNA Ligase buffer (10 μl), dNTP mix (0.5 μl, 10 mM each), T4 DNA ligase (0.5 μl) (TaKaRa #M0202L), and T4 DNA Polymerase (0.5 μl) (TaKaRa #M0203L) in 100 μl each well. After removing the buffer, add 100 μl of fill‐in solution. Incubate at RT for 2 h. After removing the supernatant, add 100 μl of 0.2% SDS. Incubate at RT for 10 min. After removing the SDS, wash the sections with 200 μl PBS three times. After removing the PBS, wash the sections with 100 μl of 1× T7 RNA Polymerase buffer in 0.05% Tween‐20/nuclease‐free water. Prepare an *in situ* transcription solution as follows: 0.05% Tween‐20/nuclease‐free water (69.4 μl), 10× T7 RNA Polymerase buffer (8 μl), 100 mM NTPs (0.5 μl each), 40 U/ml RNase Inhibitor (0.5 μl) (TaKaRa #2313A), and T7 RNA Polymerase (0.1 μl, 100 U/well) (TOYOBO #TRL‐252) in 80 μl each well. After removing the buffer, add 80 μl of *in situ* transcription solution. Seal the wells with parafilm or use a humidified chamber. Incubate at 37°C overnight.

Day 5: Library preparation. aRNA was purified using an RNAClean XP (BECKMAN #A66514) in accordance with the manufacturer’s instructions. The following SMART‐seq v4 library construction step is the same as the original ChIL (Harada *et al*, 2019; Handa *et al*, 2020) except for a smaller number of PCR cycles (15 cycles) at the library amplification. Library purification. Column purification (ZYMO #D4014) was performed to remove small by‐products (< 100 bp). The following bead purification and validation steps are the same as the original ChIL protocol (recover 200–700 bp DNA fragments).

#### Deep sequencing

Libraries were sequenced on Hiseq1500 and NovaSeq 6000 (Illumina). Reads were aligned to the GRCm38 and GRCh38 reference genome using Bowtie2 (Langmead & Salzberg, 2012) with the default option. Duplicated reads were discarded using Samtools (rmdup). The uniquely mapped reads were used for further analysis.

#### Quality assessments of ChIL‐seq data

The matrix of read counts on the equally sized (10 kb) windows on the mouse genome was generated using deepTools (Ramírez *et al*, 2016) (version. 3.4.1) with the command: *multiBamSummary bins ‐bs 10000 ‐‐ignoreDuplicates*. Pearson correlation coefficients were calculated using the log‐transformed read count (with +0.5 pseudo‐counts). The breakdown of mapped reads at the genomic regions was calculated using HOMER (annotatePeaks.pl). The library complexity was evaluated by Preseq (Daley & Smith, 2013) (ds.rSAC in the preseqR package). The theoretical case assumed uniform probabilities of obtaining reads from the mouse genome (i.e., a common expected value of the Poisson distribution). The expected number of detected genes in sub‐samples was estimated by Preseq (preseqR.interpolate.rSAC).

#### RNA‐seq analysis

Total RNA was extracted from the cryosections (up to five serial sections) using Direct‐Zol kit (Zymo #R2063). The total of 10 ng RNA was extracted for library preparation using a SMART‐Seq Stranded Kit (Takara #634444) according to the manufacturer’s instructions. Libraries were sequenced on Hiseq1500 and NovaSeq 6000 (Illumina). Gene expression quantification was performed using Salmon (Patro *et al*, 2017) *quant* with the default option.

#### Tissue‐specific enhancer analysis

Peaks of H3K27ac ChIL‐seq were called using MACS2 (Zhang *et al*, 2008) with the option: *callpeak ‐‐call‐summits ‐‐nomodel ‐‐nolambda ‐q 0.05*. Tissue specificities of the peaks were evaluated using the odds ratio in the known tissue‐specific enhancer lists (Shen *et al*, 2012). The odds ratio is defined as *(p/(1‐p))/(q/(1‐q))*, where *p* is the proportion of hits in the target tissue and *q* is the proportion of hits to the other tissues in the enhancer lists. ChromVAR (Schep *et al*, 2017) analysis was performed using consensus peaks of each tissue. The consensus peaks were constructed by taking the intersection of the peaks of three biological replicates. Typical and super‐enhancer candidates were called using HOMER (Heinz *et al*, 2010) *finePeaks* with the option: *‐style super ‐superSlope −1000 ‐gsize 3e9*. The pre‐ranked GSEA (Subramanian *et al*, 2005) was performed using tag (read) count‐ordered enhancer peaks. Then, the peaks were marked by a binary indicator overlapping with Hnf4a ChIL‐seq peaks (called by MACS2 as described above with the option: *‐q 1e‐5*).

#### Transcriptional activation analysis by PolII S5P ChIL‐seq

Aggregation plots of the gene expression percentile groups were created using agplus (Maehara & Ohkawa, 2015). The gene groups were divided according to the TPM of the bulk RNA‐seq analysis of each tissue (liver, heart, and testis).

#### Statistical modeling of traveling ratio

The read counts of PolII S5P ChIL‐seq at the TSS region (−750 to +750 bp) and TES region (0 to +1,500 bp) at all mouse transcripts were fitted to the following Poisson regression model. For each gene, we assume that the read count *y_ij_* of the *i*‐th replicate at site *j* (TSS or TES) follows the Poisson distribution, where the mean parameter *λ_ij_* satisfies the relation: *λ_ij_/M_i_ =* exp*(β_0_* *+ β_1_s_ij_)*. The offset term *M_i_* is the total reads (in millions) of the replicate *i*, and *s_ij_* is the indicator variable that the read count *y_ij_* is either TSS (*s_ij_* = *0*) or TES (*s_ij_* = *1*). Since the offsetting is equivalent to the CPM normalization of the mean count, exp*(β_0_)* and exp*(β_1_)* can be referred to as the mean CPM at TSS and the magnification factor of TES to TSS (i.e., the traveling ratio) of the gene, respectively. In R, the glm function can be used to fit the model as follows: glm(y˜site,family = poisson,offset = log(total/1e6),data = tbl), where y is the read count *y_ij_*, site is *s_ij_*, and total is the number of total reads *M*_i_. For more detail, please see our demonstrations in github (Data availability section). The model evaluates variance and can thus estimate the confidence intervals of the traveling ratio by utilizing all replicates (5 in our case) that have different total sequenced reads. We assumed that the contrasts *X − Y* (e.g., fold‐changes of TR between day 3 and day 0) follow a Gaussian distribution, and the variance was calculated from *V_X_ + V_Y_* (variances of *X* and *Y*) under the independence assumption of *X* and *Y*. *P*‐values were estimated from the model, and multiple test correction was performed using the Benjamini–Hochberg procedure in the selected genes of interest.

## Author contributions

KM, KTo, AH, and YO conceived and designed the experiments. KTa and AH performed the experiments. KM and KTa analyzed the data. KM performed statistical analysis. SS, SO, MF, NS, TH, HKu, and HKi contributed materials and analysis tools. KM, KTo, and YO wrote the paper. All authors read and approved the final manuscript.

## Conflict of interest

The authors declare that they have no conflict of interest except A.H., T.H., H. Ku., H. Ki., and Y.O. who are involved in a pending patent related to ChIL.

## Supporting information



Appendix

Dataset EV1

Dataset EV2

Dataset EV3

Dataset EV4

Dataset EV5

Dataset EV6

## Data Availability

The datasets and computer code produced in this study are available in the following databases.
RNA‐seq and ChIL‐seq data: Gene Expression Omnibus (GEO) database under accession code GSE159024 (https://www.ncbi.nlm.nih.gov/geo/query/acc.cgi?acc=GSE159024). Statistical modeling of tissue ChIL‐seq: Github (https://github.com/kazumits/tissueChIL).

## References

[msb202110323-bib-0001] Abdelaal T , Mourragui S , Mahfouz A , Reinders MJT (2020) SpaGE: spatial gene enhancement using scRNA‐seq. Nucleic Acids Res 48: e107 32955565 10.1093/nar/gkaa740 PMC7544237

[msb202110323-bib-0002] Ai S , Xiong H , Li CC , Luo Y , Shi Q , Liu Y , Yu X , Li C , He A (2019) Profiling chromatin states using single‐cell itChIP‐seq. Nat Cell Biol 21: 1164–1172 31481796 10.1038/s41556-019-0383-5

[msb202110323-bib-0003] Amatori S , Persico G , Paolicelli C , Hillje R , Sahnane N , Corini F , Furlan D , Luzi L , Minucci S , Giorgio M *et al* (2018) Epigenomic profiling of archived FFPE tissues by enhanced PAT‐ChIP (EPAT‐ChIP) technology. Clin Epigenetics 10: 143 30446010 10.1186/s13148-018-0576-y PMC6240272

[msb202110323-bib-0004] Bartman CR , Hamagami N , Keller CA , Giardine B , Hardison RC , Blobel GA , Raj A (2019) Transcriptional burst initiation and polymerase pause release are key control points of transcriptional regulation. Mol Cell 73: 519–532 30554946 10.1016/j.molcel.2018.11.004 PMC6368450

[msb202110323-bib-0005] Bartosovic M , Kabbe M , Castelo‐Branco G (2021) Single‐cell CUT&Tag profiles histone modifications and transcription factors in complex tissues. Nat Biotechnol 39: 825–835. 33846645 10.1038/s41587-021-00869-9 PMC7611252

[msb202110323-bib-0006] Blendy JA , Kaestner KH , Weinbauer GF , Nieschlag E , Schütz G (1996) Severe impairment of spermatogenesis in mice lacking the CREM gene. Nature 380: 162–165. 8600391 10.1038/380162a0

[msb202110323-bib-0007] Blondel VD , Guillaume J‐L , Lambiotte R , Lefebvre E (2008) Fast unfolding of communities in large networks. J Stat Mech: Theory Exp 2008: P10008

[msb202110323-bib-0008] Cao Z , Chen C , He B , Tan K , Lu C (2015) A microfluidic device for epigenomic profiling using 100 cells. Nat Methods 12: 959–962. 26214128 10.1038/nmeth.3488 PMC4589469

[msb202110323-bib-0009] Carter B , Ku WL , Kang JY , Hu G , Perrie J , Tang Q , Zhao K (2019) Mapping histone modifications in low cell number and single cells using antibody‐guided chromatin tagmentation (ACT‐seq). Nat Commun 10: 3747. 31431618 10.1038/s41467-019-11559-1 PMC6702168

[msb202110323-bib-0010] Cejas P , Li L , O'Neill NK , Duarte M , Rao P , Bowden M , Zhou CW , Mendiola M , Burgos E , Feliu J *et al* (2016) Chromatin immunoprecipitation from fixed clinical tissues reveals tumor‐specific enhancer profiles. Nat Med 22: 685–691. 27111282 10.1038/nm.4085

[msb202110323-bib-0011] Chen XI , Iliopoulos D , Zhang Q , Tang Q , Greenblatt MB , Hatziapostolou M , Lim E , Tam WL , Ni M , Chen Y *et al* (2014) XBP1 promotes triple‐negative breast cancer by controlling the HIF1α pathway. Nature 508: 103–107 24670641 10.1038/nature13119 PMC4105133

[msb202110323-bib-0012] Core LJ , Waterfall JJ , Lis JT (2008) Nascent RNA sequencing reveals widespread pausing and divergent initiation at human promoters. Science 322: 1845–1848 19056941 10.1126/science.1162228 PMC2833333

[msb202110323-bib-0013] Daley T , Smith AD (2013) Predicting the molecular complexity of sequencing libraries. Nat Methods 10: 325–327 23435259 10.1038/nmeth.2375 PMC3612374

[msb202110323-bib-0014] De Micheli AJ , Fraczek P , Soueid‐Baumgarten S , Ravichandran H , De Vlaminck I , Elemento O , Cosgrove BD (2020) Single‐cell analysis of the muscle stem cell hierarchy identifies heterotypic communication signals involved in skeletal muscle regeneration. Cell Rep 30: 3583–3595 32160558 10.1016/j.celrep.2020.02.067 PMC7091476

[msb202110323-bib-0015] DeLaForest A , Nagaoka M , Si‐Tayeb K , Noto FK , Konopka G , Battle MA , Duncan SA (2011) HNF4A is essential for specification of hepatic progenitors from human pluripotent stem cells. Development 138: 4143–4153 21852396 10.1242/dev.062547 PMC3171218

[msb202110323-bib-0016] Dell’Orso S , Juan AH , Ko KD , Naz F , Perovanovic J , Gutierrez‐Cruz G , Feng X , Sartorelli V (2019) Single cell analysis of adult mouse skeletal muscle stem cells in homeostatic and regenerative conditions. Development 146: dev174177 30890574 10.1242/dev.174177 PMC6602351

[msb202110323-bib-0017] Deng Y , Zhang D , Liu Y , Su G , Enninful A , Bai Z , Fan R (2021) Spatial epigenome sequencing at tissue scale and cellular level. bioRxiv 10.1101/2021.03.11.434985 [PREPRINT]

[msb202110323-bib-0018] Eng C‐H , Lawson M , Zhu Q , Dries R , Koulena N , Takei Y , Yun J , Cronin C , Karp C , Yuan G‐C *et al* (2019) Transcriptome‐scale super‐resolved imaging in tissues by RNA seqFISH+. Nature 568: 235–239 30911168 10.1038/s41586-019-1049-y PMC6544023

[msb202110323-bib-0019] Fanelli M , Amatori S , Barozzi I , Soncini M , Dal Zuffo R , Bucci G , Capra M , Quarto M , Dellino GI , Mercurio C *et al* (2010) Pathology tissue‐chromatin immunoprecipitation, coupled with high‐throughput sequencing, allows the epigenetic profiling of patient samples. Proc Natl Acad Sci 107(50): 21535–21540 21106756 10.1073/pnas.1007647107 PMC3003125

[msb202110323-bib-0020] Fanelli M , Amatori S , Barozzi I , Minucci S (2011) Chromatin immunoprecipitation and high‐throughput sequencing from paraffin‐embedded pathology tissue. Nat Protoc 6: 1905–1919. 22082985 10.1038/nprot.2011.406

[msb202110323-bib-0021] Font‐Tello A , Kesten N , Xie Y , Taing L , Varešlija D , Young LS , Hamid AA , Van Allen EM , Sweeney CJ , Gjini E *et al* (2020) FiTAc‐seq: fixed‐tissue ChIP‐seq for H3K27ac profiling and super‐enhancer analysis of FFPE tissues. Nat Protoc 15: 2503–2518 32591768 10.1038/s41596-020-0340-6

[msb202110323-bib-0022] Handa T , Harada A , Maehara K , Sato S , Nakao M , Goto N , Kurumizaka H , Ohkawa Y , Kimura H (2020) Chromatin integration labeling for mapping DNA‐binding proteins and modifications with low input. Nat Protoc 15: 3334–3360. 32807906 10.1038/s41596-020-0375-8

[msb202110323-bib-0023] Harada A , Maehara K , Handa T , Arimura Y , Nogami J , Hayashi‐Takanaka Y , Shirahige K , Kurumizaka H , Kimura H , Ohkawa Y (2019) A chromatin integration labelling method enables epigenomic profiling with lower input. Nat Cell Biol 21: 287–296. 30532068 10.1038/s41556-018-0248-3

[msb202110323-bib-0024] He P , Williams BA , Trout D , Marinov GK , Amrhein H , Berghella L , Goh S‐T , Plajzer‐Frick I , Afzal V , Pennacchio LA *et al* (2020) The changing mouse embryo transcriptome at whole tissue and single‐cell resolution. Nature 583: 760–767 32728245 10.1038/s41586-020-2536-x PMC7410830

[msb202110323-bib-0025] Heck KL , van Belle G , Simberloff D (1975) Explicit calculation of the rarefaction diversity measurement and the determination of sufficient sample size. Ecology 56: 1459–1461

[msb202110323-bib-0026] Heinz S , Benner C , Spann N , Bertolino E , Lin YC , Laslo P , Cheng JX , Murre C , Singh H , Glass CK (2010) Simple combinations of lineage‐determining transcription factors prime cis‐regulatory elements required for macrophage and B cell identities. Mol Cell 38: 576–589 20513432 10.1016/j.molcel.2010.05.004 PMC2898526

[msb202110323-bib-0027] Joo MS , Koo JH , Kim TH , Kim YS , Kim SG (2019) LRH1‐driven transcription factor circuitry for hepatocyte identity: Super‐enhancer cistromic analysis. EBioMedicine 40: 488–503 30638865 10.1016/j.ebiom.2018.12.056 PMC6413675

[msb202110323-bib-0028] Kaya‐Okur HS , Wu SJ , Codomo CA , Pledger ES , Bryson TD , Henikoff JG , Ahmad K , Henikoff S . CUT&Tag for efficient epigenomic profiling of small samples and single cells. Nat Commun 10: 1930 10.1038/s41467-019-09982-5 PMC6488672 31036827

[msb202110323-bib-0029] Kimura H , Hayashi‐Takanaka Y , Goto Y , Takizawa N , Nozaki N (2008) The organization of histone H3 modifications as revealed by a panel of specific monoclonal antibodies. Cell Struct Funct 33: 61–73 18227620 10.1247/csf.07035

[msb202110323-bib-0030] Komarnitsky P (2000) Different phosphorylated forms of RNA polymerase II and associated mRNA processing factors during transcription. Genes Dev 14: 2452–2460 11018013 10.1101/gad.824700 PMC316976

[msb202110323-bib-0031] Korotkevich G , Sukhov V , Sergushichev A . Fast gene set enrichment analysis. Bioinformatics 32: 1891–1894. 10.1093/bioinformatics/btw030 26833345

[msb202110323-bib-0032] Ku WL , Nakamura K , Gao W , Cui K , Hu G , Tang Q , Ni B , Zhao K (2019) Single‐cell chromatin immunocleavage sequencing (scChIC‐seq) to profile histone modification. Nat Methods 16: 323–325 30923384 10.1038/s41592-019-0361-7 PMC7187538

[msb202110323-bib-0033] Langmead B , Salzberg SL (2012) Fast gapped‐read alignment with Bowtie 2. Nat Methods 9: 357–359 22388286 10.1038/nmeth.1923 PMC3322381

[msb202110323-bib-0034] Latroche C , Weiss‐Gayet M , Muller L , Gitiaux C , Leblanc P , Liot S , Ben‐Larbi S , Abou‐Khalil R , Verger N , Bardot P *et al* (2017) Coupling between myogenesis and angiogenesis during skeletal muscle regeneration is stimulated by restorative macrophages. Stem Cell Reports 9: 2018–2033 29198825 10.1016/j.stemcr.2017.10.027 PMC5785732

[msb202110323-bib-0035] Li H , Handsaker B , Wysoker A , Fennell T , Ruan J , Homer N , Marth G , Abecasis G , Durbin R , 1000 Genome Project Data Processing Subgroup (2009) The sequence alignment/map format and SAMtools. Bioinformatics 25: 2078–2079 19505943 10.1093/bioinformatics/btp352 PMC2723002

[msb202110323-bib-0036] Liberzon A , Subramanian A , Pinchback R , Thorvaldsdottir H , Tamayo P , Mesirov JP (2011) Molecular signatures database (MSigDB) 3.0. Bioinformatics 27: 1739–1740 21546393 10.1093/bioinformatics/btr260 PMC3106198

[msb202110323-bib-0037] Lie PPY , Mruk DD , Lee WM , Cheng CY (2009) Epidermal growth factor receptor pathway substrate 8 (Eps8) is a novel regulator of cell adhesion and the blood‐testis barrier integrity in the seminiferous epithelium. FASEB J 23: 2555–2567 19293393 10.1096/fj.06-070573 PMC2717761

[msb202110323-bib-0038] Liu C , Wang M , Wei X , Wu L , Xu J , Dai X , Xia J , Cheng M , Yuan Y , Zhang P *et al* (2019) An ATAC‐seq atlas of chromatin accessibility in mouse tissues. Sci Data 6: 65 31110271 10.1038/s41597-019-0071-0 PMC6527694

[msb202110323-bib-0039] Liu Y , Zhou J , White KP (2014) RNA‐seq differential expression studies: More sequence or more replication? Bioinformatics 30: 301–304 24319002 10.1093/bioinformatics/btt688 PMC3904521

[msb202110323-bib-0040] Lovén J , Hoke HA , Lin CY , Lau A , Orlando DA , Vakoc CR , Bradner JE , Lee TI , Young RA (2013) Selective inhibition of tumor oncogenes by disruption of super‐enhancers. Cell 153: 320–334 23582323 10.1016/j.cell.2013.03.036 PMC3760967

[msb202110323-bib-0041] Machado L , Relaix F , Mourikis P (2021) Stress relief: emerging methods to mitigate dissociation‐induced artefacts. Trends Cell Biol 31: 888–897 34074577 10.1016/j.tcb.2021.05.004

[msb202110323-bib-0042] Maehara K , Ohkawa Y (2015) Agplus: A rapid and flexible tool for aggregation plots. Bioinformatics 31: 3046–3047 25995229 10.1093/bioinformatics/btv322

[msb202110323-bib-0043] Miyawaki‐Kuwakado A , Wu Q , Harada A , Tomimatsu K , Fujii T , Maehara K , Ohkawa Y (2021) Transcriptome analysis of gene expression changes upon enzymatic dissociation in skeletal myoblasts. Genes Cells 26: 530–540 33987903 10.1111/gtc.12870

[msb202110323-bib-0044] Muse GW , Gilchrist DA , Nechaev S , Shah R , Parker JS , Grissom SF , Zeitlinger J , Adelman K (2007) RNA polymerase is poised for activation across the genome. Nat Genet 39: 1507–1511 17994021 10.1038/ng.2007.21 PMC2365887

[msb202110323-bib-0045] Naito A , Sumida T , Nomura S , Liu M‐L , Higo T , Nakagawa A , Okada K , Sakai T , Hashimoto A , Hara Y *et al* (2012) Complement C1q activates canonical Wnt signaling and promotes aging‐related phenotypes. Cell 150: 659–660 10.1016/j.cell.2012.03.047 PMC3529917 22682250

[msb202110323-bib-0046] Newman AM , Steen CB , Long Liu C , Gentles AJ , Chaudhuri AA , Scherer F , Khodadoust MS , Esfahani MS , Luca BA , Steiner D *et al* Determining cell type abundance and expression from bulk tissues with digital cytometry. Nat Biotechnol 37: 773–782 31061481 10.1038/s41587-019-0114-2 PMC6610714

[msb202110323-bib-0047] Odawara J , Harada A , Yoshimi T , Maehara K , Tachibana T , Okada S , Akashi K , Ohkawa Y (2011) The classification of mRNA expression levels by the phosphorylation state of RNAPII CTD based on a combined genome‐wide approach. BMC Genom 12: 516 10.1186/1471-2164-12-516 PMC3209707 22011111

[msb202110323-bib-0048] Ono Y , Calhabeu F , Morgan JE , Katagiri T , Amthor H , Zammit PS (2011) BMP signalling permits population expansion by preventing premature myogenic differentiation in muscle satellite cells. Cell Death Differ 18: 222–234 20689554 10.1038/cdd.2010.95 PMC3044455

[msb202110323-bib-0049] Oprescu SN , Yue F , Qiu J , Brito LF , Kuang S (2020) Temporal dynamics and heterogeneity of cell populations during skeletal muscle regeneration. iScience 23: 100993 32248062 10.1016/j.isci.2020.100993 PMC7125354

[msb202110323-bib-0050] Patro R , Duggal G , Love MI , Irizarry RA , Kingsford C (2017) Salmon provides fast and bias‐aware quantification of transcript expression. Nat Methods 14: 417–419 28263959 10.1038/nmeth.4197 PMC5600148

[msb202110323-bib-0051] Picelli S , Björklund ÅK , Reinius B , Sagasser S , Winberg G , Sandberg R (2014) Tn5 transposase and tagmentation procedures for massively scaled sequencing projects. Genome Res 24: 2033–2040 25079858 10.1101/gr.177881.114 PMC4248319

[msb202110323-bib-0052] Rahl PB , Lin CY , Seila AC , Flynn RA , McCuine S , Burge CB , Sharp PA , Young RA (2010) C‐Myc regulates transcriptional pause release. Cell 141: 432–445 20434984 10.1016/j.cell.2010.03.030 PMC2864022

[msb202110323-bib-0053] Rahmani E , Schweiger R , Rhead B , Criswell LA , Barcellos LF , Eskin E , Rosset S , Sankararaman S , Halperin E . Cell‐type‐specific resolution epigenetics without the need for cell sorting or single‐cell biology. Nat Commun 10: 3417 31366909 10.1038/s41467-019-11052-9 PMC6668473

[msb202110323-bib-0054] Ramírez F , Ryan DP , Grüning B , Bhardwaj V , Kilpert F , Richter AS , Heyne S , Dündar F , Manke T (2016) deepTools2: a next generation web server for deep‐sequencing data analysis. Nucleic Acids Res 44: W160–W165. 27079975 10.1093/nar/gkw257 PMC4987876

[msb202110323-bib-0055] Roadmap Epigenomics Consortium , Kundaje A , Meuleman W , Ernst J , Bilenky M , Yen A , Heravi‐Moussavi A , Kheradpour P , Zhang Z , Wang J *et al* (2015) Integrative analysis of 111 reference human epigenomes. Nature 518: 317–330 25693563 10.1038/nature14248 PMC4530010

[msb202110323-bib-0056] Rotem A , Ram O , Shoresh N , Sperling RA , Goren A , Weitz DA , Bernstein BE (2015) Single‐cell ChIP‐seq reveals cell subpopulations defined by chromatin state. Nat Biotechnol 33: 1165–1172 26458175 10.1038/nbt.3383 PMC4636926

[msb202110323-bib-0057] Sassone‐Corsi P (2002) Unique chromatin remodeling and transcriptional regulation in spermatogenesis. Science 296: 2176–2178 12077401 10.1126/science.1070963

[msb202110323-bib-0058] Schaum N , Karkanias J , Neff NF , May AP , Quake SR , Wyss‐Coray T , Darmanis S , Batson J , Botvinnik O , Chen MB *et al* (2018) Single‐cell transcriptomics of 20 mouse organs creates a Tabula Muris the tabula Muris consortium*. Nature 562: 367–372. 30283141 10.1038/s41586-018-0590-4 PMC6642641

[msb202110323-bib-0059] Schep AN , Wu B , Buenrostro JD , Greenleaf WJ (2017) chromVAR: inferring transcription‐factor‐associated accessibility from single‐cell epigenomic data. Nat Methods 14: 975–978 28825706 10.1038/nmeth.4401 PMC5623146

[msb202110323-bib-0060] Shang Y , Myers M , Brown M (2002) Formation of the androgen receptor transcription complex. Mol Cell 9: 601–610 11931767 10.1016/s1097-2765(02)00471-9

[msb202110323-bib-0061] Shen Y , Yue F , McCleary DF , Ye Z , Edsall L , Kuan S , Wagner U , Dixon J , Lee L , Lobanenkov VV *et al* (2012) A map of the cis‐regulatory sequences in the mouse genome. Nature 488: 116–120 22763441 10.1038/nature11243 PMC4041622

[msb202110323-bib-0062] Skene PJ , Henikoff JG , Henikoff S (2018) Targeted in situ genome‐wide profiling with high efficiency for low cell numbers. Nat Protoc 13: 1006–1019 29651053 10.1038/nprot.2018.015

[msb202110323-bib-0063] Skene PJ , Henikoff S (2017) An efficient targeted nuclease strategy for high‐resolution mapping of DNA binding sites. eLife 6: e21856 28079019 10.7554/eLife.21856 PMC5310842

[msb202110323-bib-0064] Stasevich TJ , Hayashi‐Takanaka Y , Sato Y , Maehara K , Ohkawa Y , Sakata‐Sogawa K , Tokunaga M , Nagase T , Nozaki N , McNally JG *et al* (2014) Regulation of RNA polymerase II activation by histone acetylation in single living cells. Nature 516: 272 25252976 10.1038/nature13714

[msb202110323-bib-0065] Stelloo S , Nevedomskaya E , Kim Y , Schuurman K , Valle‐Encinas E , Lobo J , Krijgsman O , Peeper DS , Chang SL , Feng F‐C *et al* (2018) Integrative epigenetic taxonomy of primary prostate cancer. Nat Commun 9: 4900 30464211 10.1038/s41467-018-07270-2 PMC6249266

[msb202110323-bib-0066] Stevens J , Ermakov A , Braganca J , Hilton H , Underhill P , Bhattacharya S , Brown NA , Norris DP (2010) Analysis of the asymmetrically expressed Ablim1 locus reveals existence of a lateral plate Nodal‐independent left sided signal and an early, left‐right independent role for nodal flow. BMC Dev Biol 10: 54 20487527 10.1186/1471-213X-10-54 PMC2885315

[msb202110323-bib-0067] Stuart T , Butler A , Hoffman P , Hafemeister C , Papalexi E , Mauck WM , Hao Y , Stoeckius M , Smibert P , Satija R (2019) Comprehensive integration of single‐cell data. Cell 177: 1888–1902 31178118 10.1016/j.cell.2019.05.031 PMC6687398

[msb202110323-bib-0068] Stunnenberg HG , Hirst M (2016) The international human Epigenome consortium: a blueprint for scientific collaboration and discovery. Cell 167: 1897 10.1016/j.cell.2016.12.002 27984737

[msb202110323-bib-0069] Subramanian A , Tamayo P , Mootha VK , Mukherjee S , Ebert BL , Gillette MA , Paulovich A , Pomeroy SL , Golub TR , Lander ES *et al* (2005) Gene set enrichment analysis: A knowledge‐based approach for interpreting genome‐wide expression profiles. Proc Natl Acad Sci 102: 15545–15550 16199517 10.1073/pnas.0506580102 PMC1239896

[msb202110323-bib-0070] Sun H , Wu J , Wickramasinghe P , Pal S , Gupta R , Bhattacharyya A , Agosto‐Perez FJ , Showe LC , Huang THM , Davuluri RV (2011) Genome‐wide mapping of RNA Pol‐II promoter usage in mouse tissues by ChIP‐seq. Nucleic Acids Res 39: 190–201 20843783 10.1093/nar/gkq775 PMC3017616

[msb202110323-bib-0071] Tidball JG (2005) Inflammatory processes in muscle injury and repair. Am J Physiol Regul Integr Comp Physiol. 288: R345–R353 15637171 10.1152/ajpregu.00454.2004

[msb202110323-bib-0072] Van den Berge K , Hembach KM , Soneson C , Tiberi S , Clement L , Love MI , Patro R , Robinson MD (2019) RNA sequencing data: Hitchhiker’s guide to expression analysis. Annu Rev Biomed Data Sci 2: 139–173

[msb202110323-bib-0073] Wang J‐H , Wang Q‐J , Wang C , Reinholt B , Grant AL , Gerrard DE , Kuang S (2015) Heterogeneous activation of a slow myosin gene in proliferating myoblasts and differentiated single myofibers. Dev Biol 402: 72–80. 25794679 10.1016/j.ydbio.2015.02.025 PMC4435531

[msb202110323-bib-0074] Wang Q , Xiong H , Ai S , Yu X , Liu Y , Zhang J , He A (2019) CoBATCH for high‐throughput single‐cell epigenomic profiling. Mol Cell 76: 206–216 31471188 10.1016/j.molcel.2019.07.015

[msb202110323-bib-0075] Watt AJ , Garrison WD , Duncan SA (2003) HNF4: a central regulator of hepatocyte differentiation and function. Hepatology 37: 1249–1253 12774000 10.1053/jhep.2003.50273

[msb202110323-bib-0076] Whyte WA , Orlando DA , Hnisz D , Abraham BJ , Lin CY , Kagey MH , Rahl PB , Lee TI , Young RA (2013) Master transcription factors and mediator establish super‐enhancers at key cell identity genes. Cell 153: 307–319 23582322 10.1016/j.cell.2013.03.035 PMC3653129

[msb202110323-bib-0077] Wosczyna MN , Rando TA (2018) A Muscle Stem Cell Support Group: Coordinated Cellular Responses in Muscle Regeneration. Dev Cell 46: 135–143 30016618 10.1016/j.devcel.2018.06.018 PMC6075730

[msb202110323-bib-0078] Zaitsev K , Bambouskova M , Swain A , Artyomov MN (2019) Complete deconvolution of cellular mixtures based on linearity of transcriptional signatures. Nat Commun 10: 2209 31101809 10.1038/s41467-019-09990-5 PMC6525259

[msb202110323-bib-0079] Zeitlinger J , Stark A , Kellis M , Hong JW , Nechaev S , Adelman K , Levine M , Young RA (2007) RNA polymerase stalling at developmental control genes in the Drosophila melanogaster embryo. Nat Genet 39: 1512–1516 17994019 10.1038/ng.2007.26 PMC2824921

[msb202110323-bib-0080] Zhang Y , Liu T , Meyer CA , Eeckhoute J , Johnson DS , Bernstein BE , Nussbaum C , Myers RM , Brown M , Li W *et al* (2008) Model‐based analysis of ChIP‐Seq (MACS). Genome Biol 9: R137 18798982 10.1186/gb-2008-9-9-r137 PMC2592715

[msb202110323-bib-0081] Zhao NA , Cao J , Xu L , Tang Q , Dobrolecki LE , Lv X , Talukdar M , Lu Y , Wang X , Hu DZ *et al* (2018) Pharmacological targeting of MYC‐regulated IRE1/XBP1 pathway suppresses MYC‐driven breast cancer. Journal of Clinical Investigation 128: 1283–1299 10.1172/JCI95873 PMC5873887 29480818

[msb202110323-bib-0082] Zhong J , Ye Z , Clark CR , Lenz SW , Nguyen JH , Yan H , Robertson KD , Farrugia G , Zhang Z , Ordog T *et al* (2019) Enhanced and controlled chromatin extraction from FFPE tissues and the application to ChIP‐seq. BMC Genom 20: 249 10.1186/s12864-019-5639-8 PMC6440302 30922218

