# Supplementary material for: Modeling population size independent tissue epigenomes by ChIL‐seq with single thin sections
Source: Mol Syst Biol. 2021 Nov 3;17(11):e10323. doi: 10.15252/msb.202110323 (PMC8564819; doi:10.15252/msb.202110323)
Supplement: Supplementary file 1 — Appendix [file MSB-17-e10323-s003.pdf]

# Appendix Figures

## Modeling population size independent tissue epigenomes by ChIL-seq with single-thin sections

Kazumitsu Maehara[1], Kosuke Tomimatsu[1], Akihito Harada[1], Kaori Tanaka[1], Shoko Sato[2], Megumi Fukuoka[3], Seiji Okada[4], Tetsuya Handa[5], Hitoshi Kurumizaka[2], Noriko Saitoh[3], Hiroshi Kimura[5], and Yasuyuki Ohkawa[1*]

[1]Division of Transcriptomics, Medical Institute of Bioregulation, Kyushu University, 3-1-1 Maidashi, Higashi-ku, Fukuoka 812-0054, Japan.

[2]Institute for Quantitative Biosciences, The University of Tokyo, 1-1-1 Yayoi, Bunkyo-ku, Tokyo 113-0032, Japan.

[3]Division of Cancer Biology, The Cancer Institute of Japanese Foundation for Cancer Research, Tokyo, Japan

[4]Division of Pathophysiology, Medical Institute of Bioregulation, Kyushu University, 3-1-1 Maidashi, Higashi-ku, Fukuoka 812-0054, Japan.

[5]Cell Biology Center, Institute of Innovative Research, Tokyo Institute of Technology, 4259 Nagatsuta, Midori-ku, Yokohama 226-8503, Japan

[*]Corresponding author: Yasuyuki Ohkawa

**Email:** yohkawa@bioreg.kyushu-u.ac.jp

## Contents

**Appendix Figure S1: Immunofluorescent images of whole sections stained with the ChIL-probe** .......... 3

**Appendix Figure S2: Promoter escape of PolII** ........ 4

**Appendix Figure S3: Predictive performance of ChIL-seq and other methods for tissue epigenome analysis** ........ 5

**Appendix Figure S4: Super-enhancer analysis of each replicate** ........ 6

**Appendix Figure S5: Motifs and enhancers are enriched in Hnf4α ChIL-seq peaks** ........ 7

**Appendix Figure S6: Tolerant definition of active genes by RNA-seq** ........ 8

**Appendix Figure S7: Statistical modeling of PolII reveals transcriptional dynamics in muscle regeneration** ........ 9

**Appendix Figure S8: The statistical test is sensitive to the definition of the traveling ratio** ........ 11

**Appendix Figure S9: Exploration of chromatin-regulated genes in muscle regeneration** ........ 12

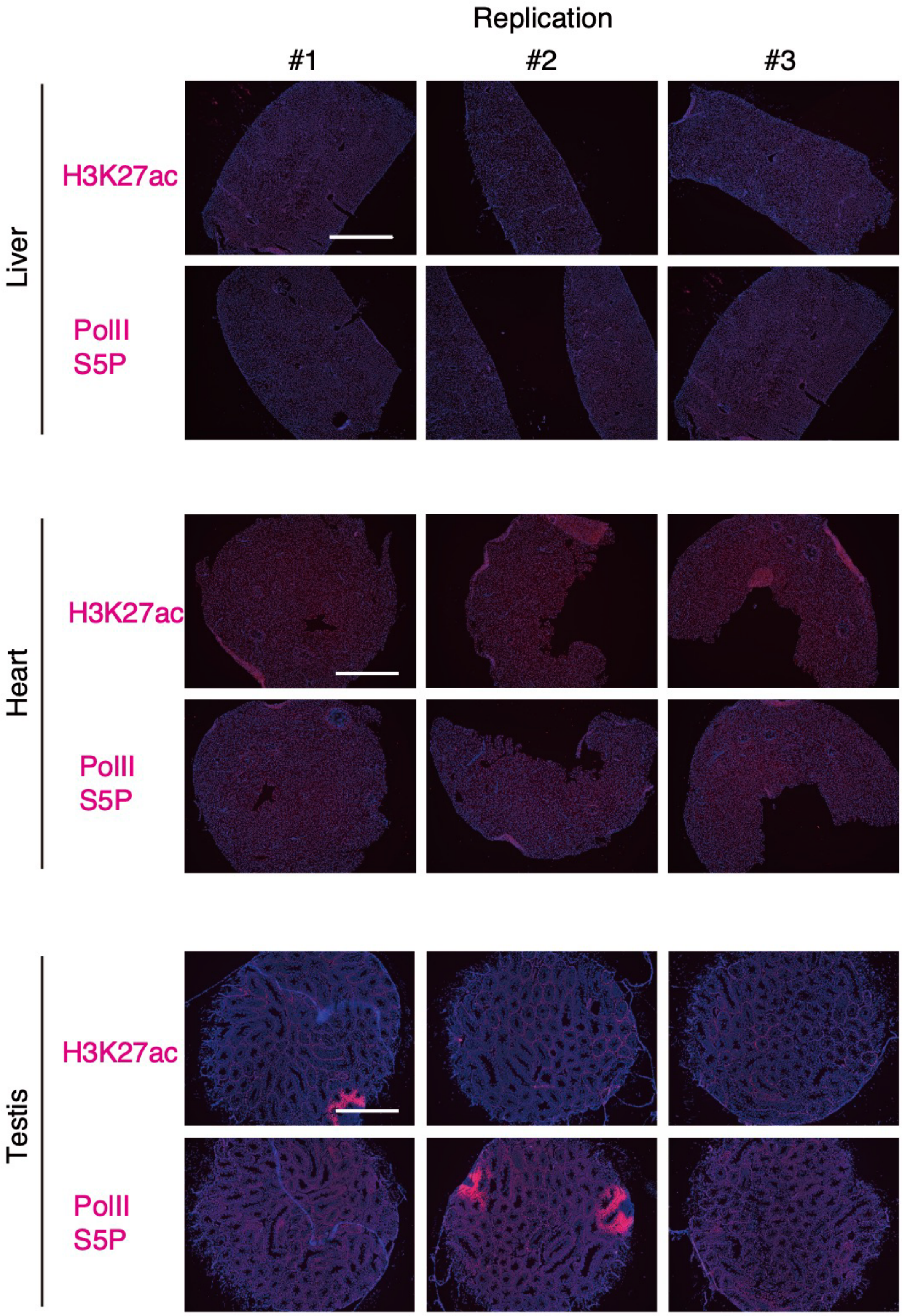


**Appendix Figure S1: Immunofluorescent images of whole sections stained with the ChIL-probe.** Immunofluorescent images of the indicated tissues for all replicates (*N*=3). Tissue sections were stained with H3K27ac or PolII S5P antibody and visualized using the fluorescent dye–conjugated ChIL-probe. DNA was counterstained with Hoechst 33342. Scale bar: 1 mm.

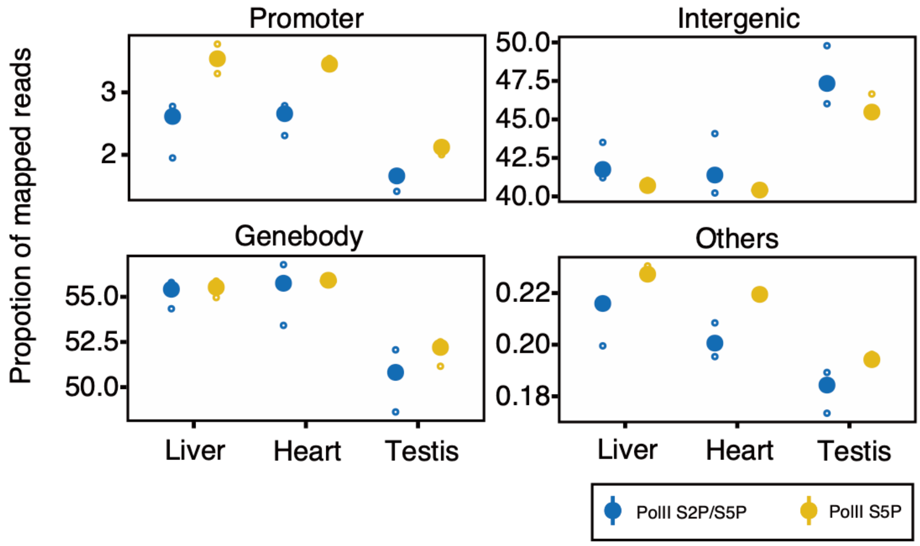


**Appendix Figure S2: Promoter escape of PolII.**

Proportions of mapped reads of PolII S5P and S2PS5P in the annotated genomic regions were compared. The difference in proportions of S5P and S2PS5P on the promoter region was statistically significant; for all tissues, Wald *p*-value was less than $1\times10^{-16}$ on $\beta_1$ ($\beta_1 = 0$ if S5P = S2PS5P in the proportion of mapped reads) in the log-linear model of $\log\mu = \beta_0 + \beta_1 x + \log M$, where $\mu$ is the read count on the promoter, $M$ *is the total number of reads*, and *x*=1 if the data are S2PS5P otherwise 0.

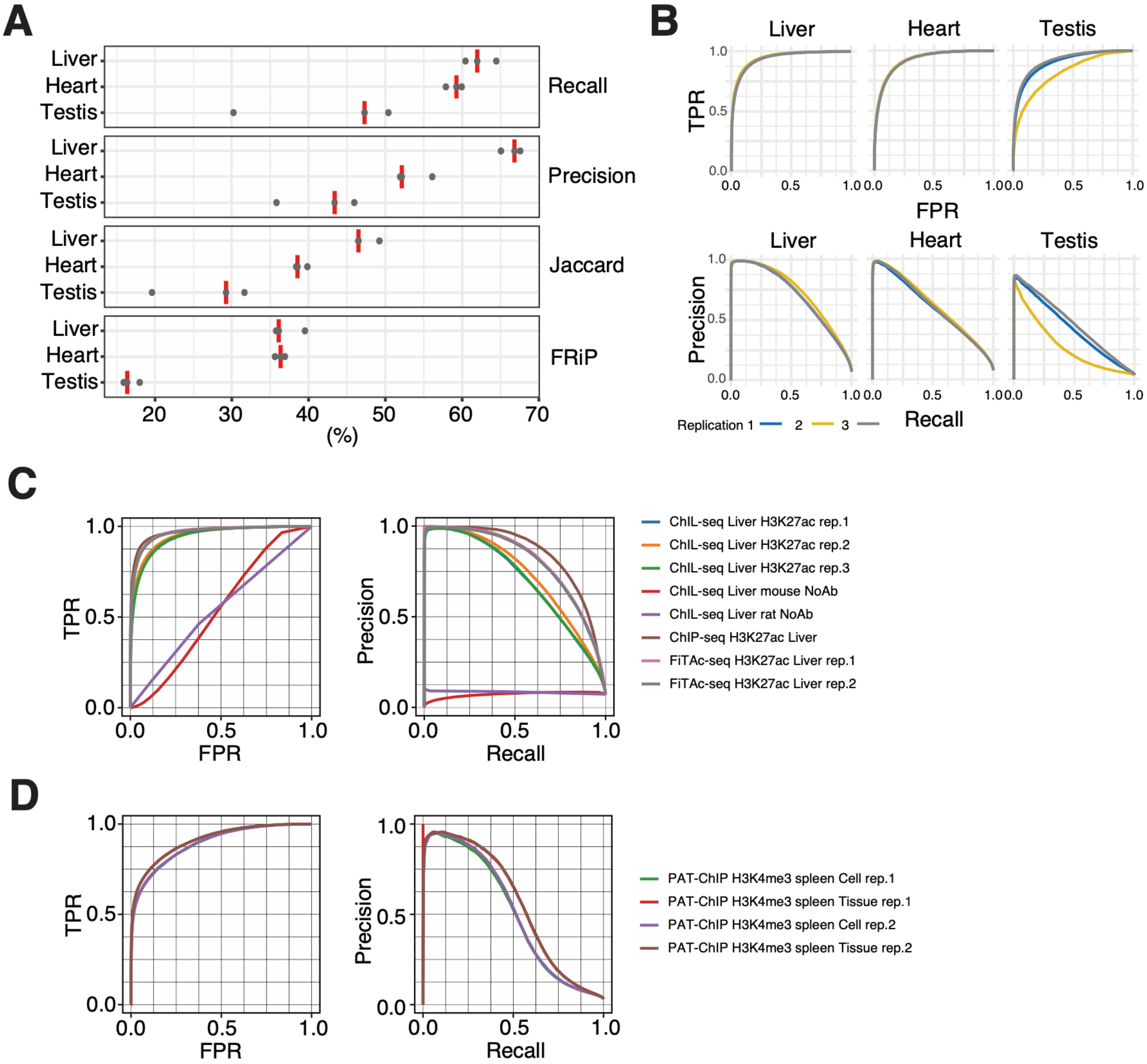


**Appendix Figure S3: Predictive performance of ChIL-seq and other methods for tissue epigenome analysis**

**(A)** Performance metrics of ChIL H3K27ac. The values indicate the consistency between ChIL-seq and gold standard ChIP-seq peaks in corresponding tissue samples. Vertical red lines are the median value of each metric. **(B)** Receiver operator characteristic (ROC) (top) and precision-recall (PR) (bottom) curves created at evaluation of each performance metrics shown in (A)**. (C-D)** ROC- and PR-curves for the performance comparison of H3K27ac ChIL-seq and FiTAc-seq in liver tissue and H3K4me3 PAT-ChIP-seq in spleen tissue. The details of the calculated performance measures were listed in Dataset EV3.

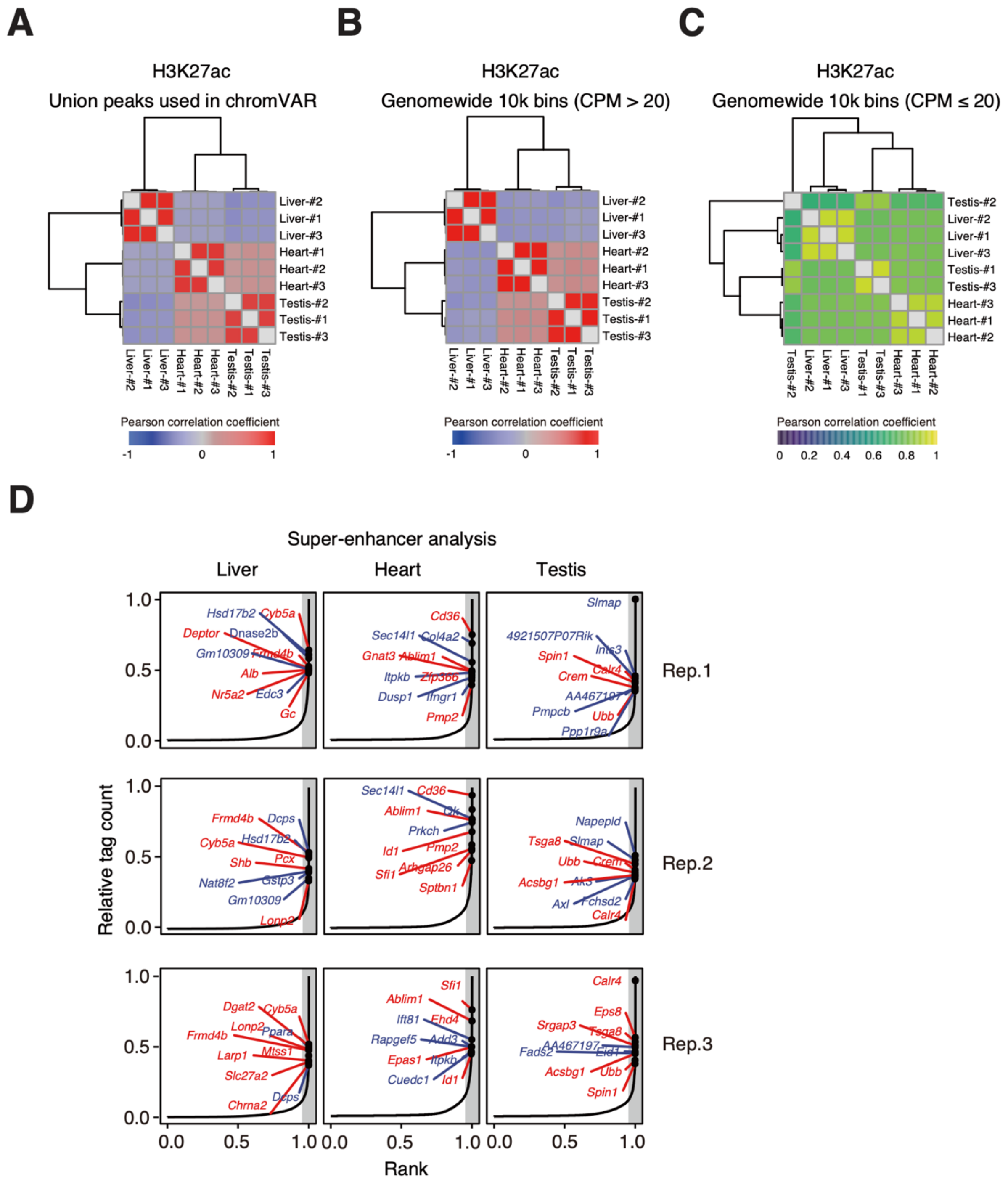


**Appendix Figure S4: Super-enhancer analysis of each replicate.**

Pearson correlation of ChIL H2K27ac signals used in the chromVAR evaluation **(A)**, the correlation in the genome-wide 10K bins with CPM>20 **(B)** and 10K bins with CPM≤20 **(C)**. The %bins satisfying CPM>20 were 1.76-2.10 in liver, 1.02-1.24 in heart, 0.26-0.44 in testis. The %reads in the CPM>20 bins were 15.94-19.92 in liver, 8.01-9.88 in heart, 1.93-3.23 in testis. **(D)** Tissue-specific enhancers are identified so that they are listed more than twice (twice: blue, all: red) in the top 5% of tag count among enhancer candidates and are not in the SEs of other tissues. Grey shades indicate the top 5% of tag count among the enhancer candidates.

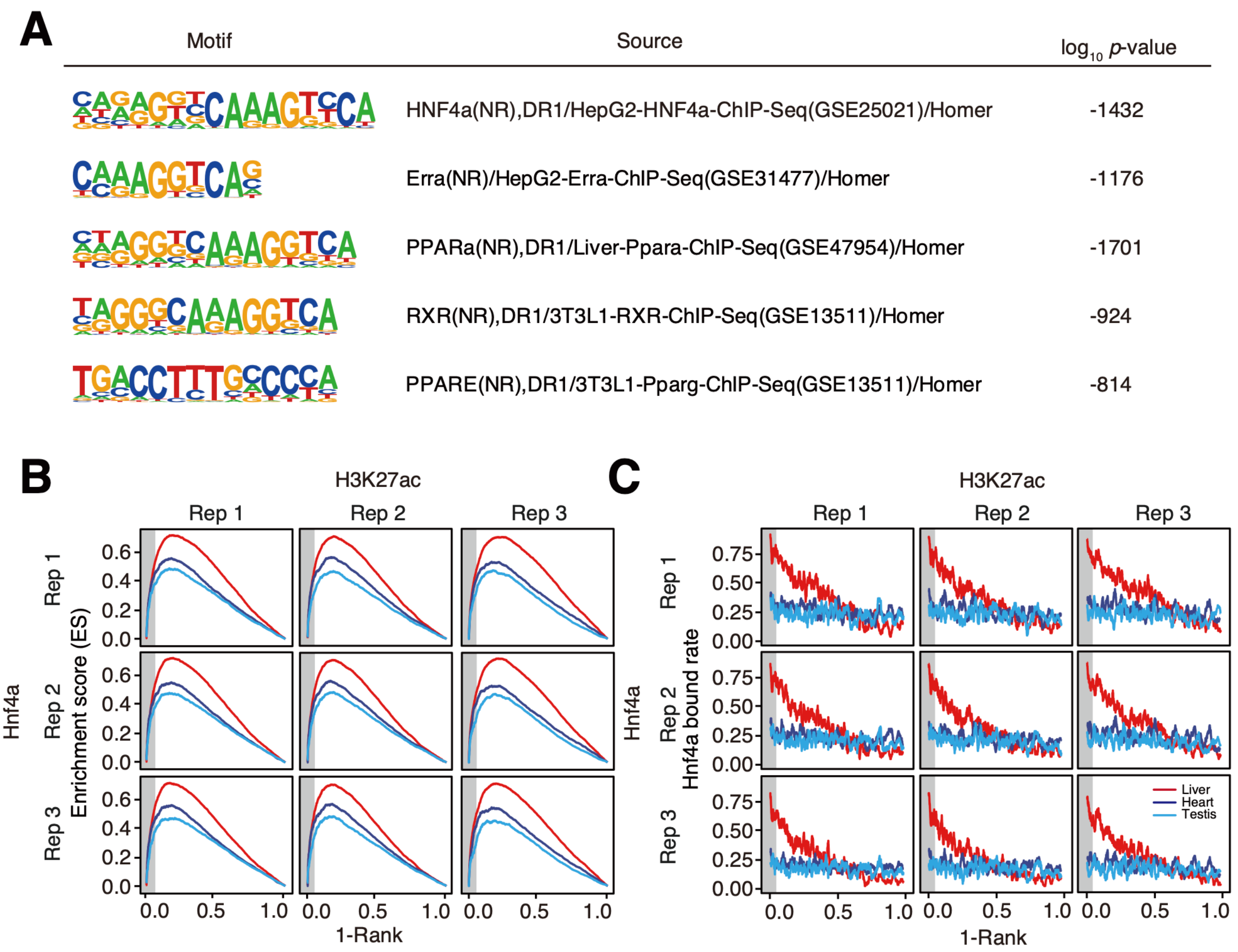


**Appendix Figure S5: Motifs and enhancers are enriched in Hnf4α ChIL-seq peaks.**

**(A)** Enrichment analysis of *known motifs* using HOMER. The motifs shown here are the top 5 based on the *p*-values. Their enrichment of motifs was evaluated within 250 bp from a summit of MACS2 peaks. The height of the motif logos corresponds to nucleotide frequencies. **(B)** Gene set enrichment analysis of Hnf4α-bound genes, and **(C)** the rate of Hnf4α-bound genes in the sliding windows of 100 genes along the ordered enhancers. All possible combinations (3 × 3 combination of replicates for ChIL H3K27ac and ChIL Hnf4α) are shown.

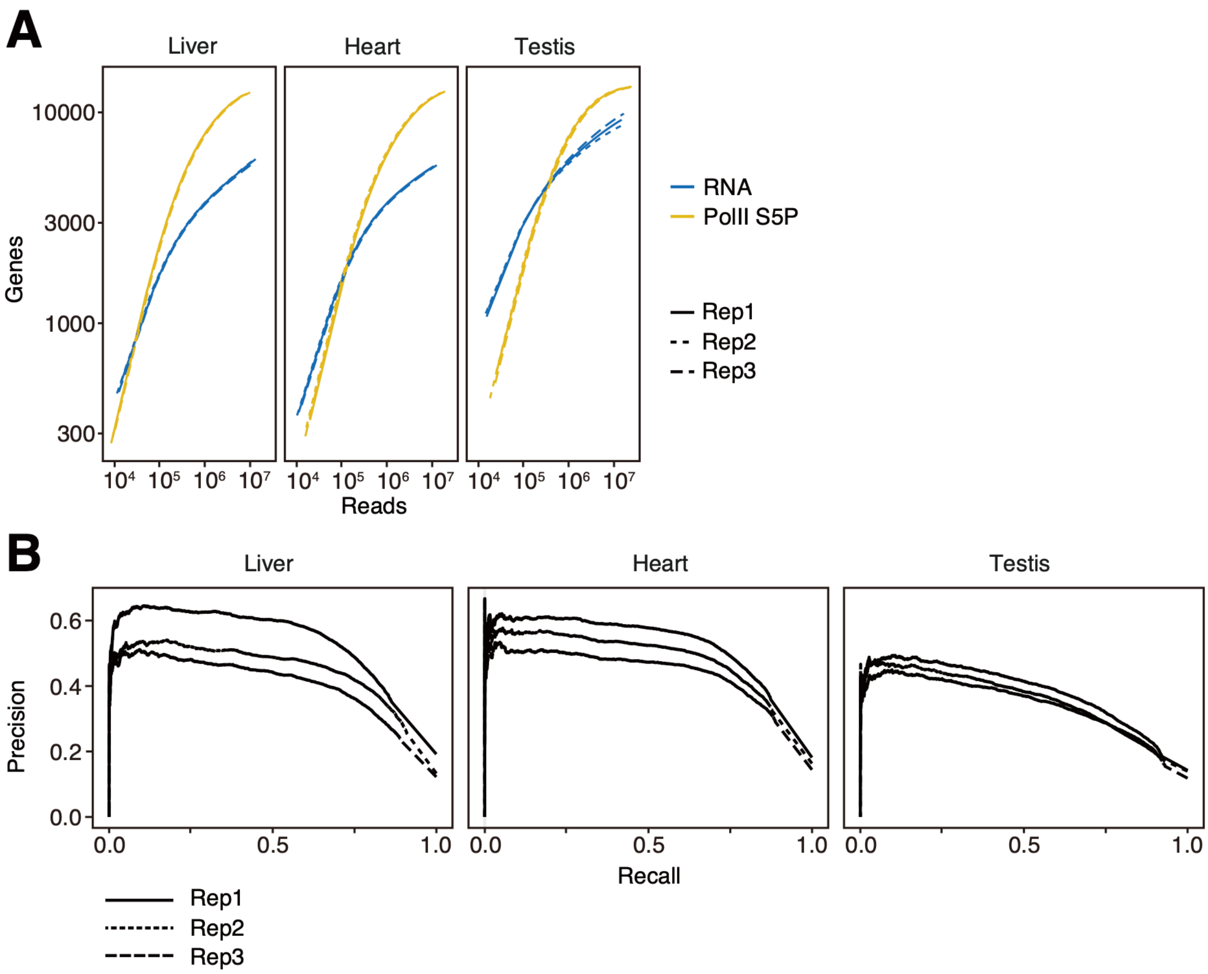


**Appendix Figure S6: Tolerant definition of *active genes* by RNA-seq.**

**(A)** Expected number of detected genes in sub-samples of total sequenced reads compared between RNA-seq (RNA) and ChIL PolII S5P. **(B)** Precision and recall curves for predicting ChIL PolII peaks based on TPM values are shown. The recall represents the proportion of PolII S5P peaks covered by the active genes, and the precision is the proportion of active genes covered by the PolII S5P peaks. Active genes are defined at each TPM threshold.

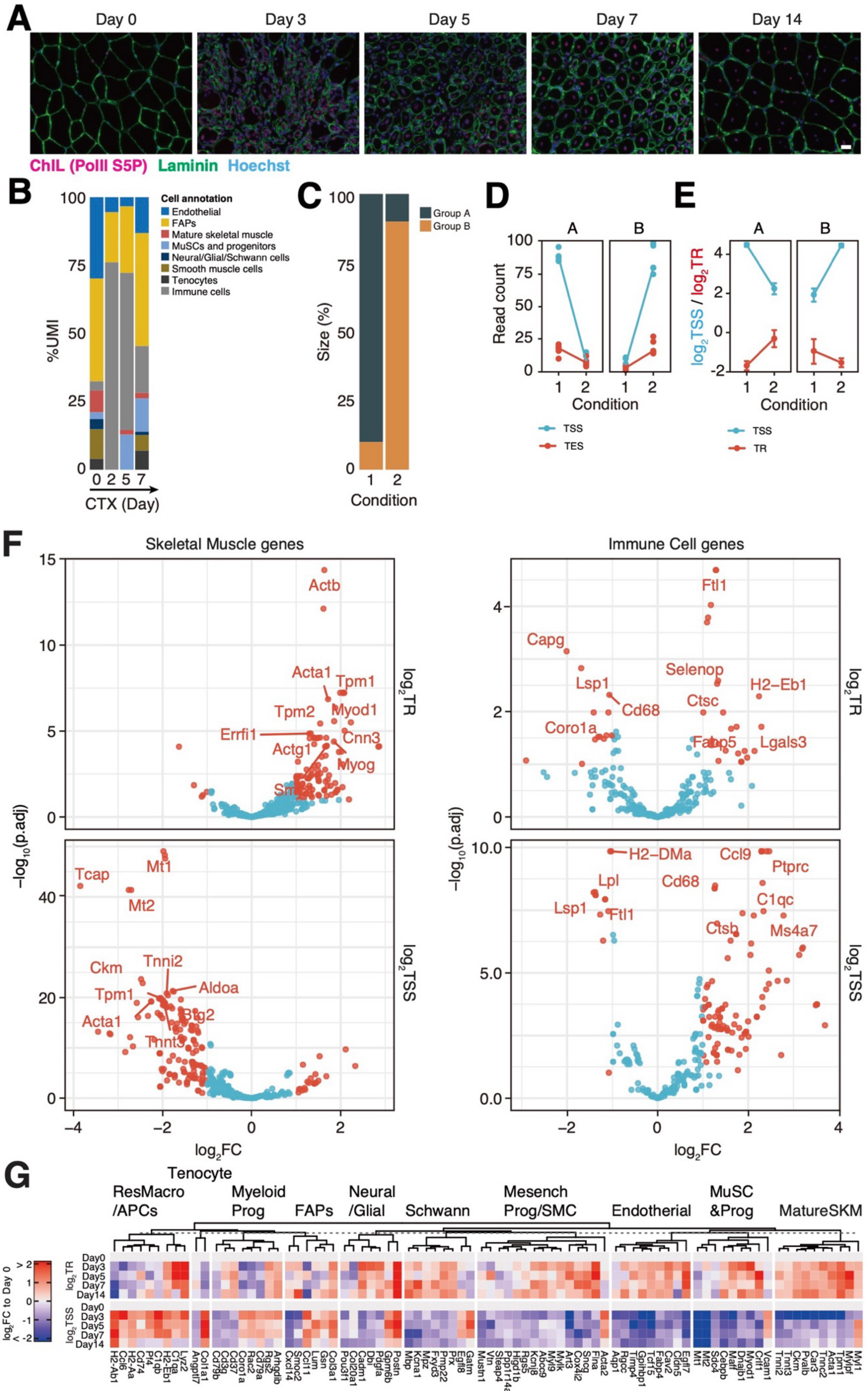
A
Day 0
Day 3
Day 5
Day 7
Day 14
ChIL (PolII S5P) Laminin Hoechst
B
Cell annotation
Endothelial
FAPs
Mature skeletal muscle
MuSCs and progenitors
Neural/Glial/Schwann cells
Smooth muscle cells
Tenocytes
Immune cells
%UMI
CTX (Day)
C
Group A
Group B
Size (%)
Condition
D
Read count
Condition
TSS
TES
E
log2TSS / log2TR
Condition
TSS
TR
F
Skeletal Muscle genes
Immune Cell genes
−log10(p.adj)
log2TR
log2TSS
log2FC
Actb
Acta1
Tpm1
Tpm2
Myod1
Errfi1
Cnn3
Actg1
Myog
Tcap
Mt1
Mt2
Ckm
Tnni2
Aldoa
Btg2
Tnnt3
Ftl1
Capg
Selenop
Lsp1
H2−Eb1
Ctsc
Cd68
Coro1a
Fabp5
Lgals3
H2−DMa
Ccl9
Ptprc
Lpl
C1qc
Ctsb
Ms4a7
G
Tenocyte
ResMacro /APCs
Myeloid Prog
FAPs
Neural /Glial
Schwann
Mesench Prog/SMC
Endotherial
MuSC &Prog
MatureSKM
log2FC to Day 0
Day0
Day3
Day5
Day7
Day14

**Appendix Figure S7: Statistical modeling of PolII reveals transcriptional dynamics in muscle regeneration.**

**(A)** The complete immunofluorescent images shown in Figure 4a. Scale bar: 20 μm. **(B)** Proportion of sequenced reads (%UMI) occupied by the annotated cell types in muscle regeneration. The single cell data (GSE143437) by De Micheli et al. was re-analyzed. **(C)** Proportion of two cell types in two conditions in artificial data. Group A occupies 10% of tissue in condition 1 and increases to 90% in condition 2**.** Group B occupies 90% of tissue in condition 1 and decreases to 10% in condition 2**. (D)** Simulated read counts of the two groups at TSS and TES in artificial data. The transcriptional state of group A was activated in condition 2 (TR was set to ×2 of condition 1). However, in this plot, the transcriptional state change was confounded by the large change in population size. **(E)** Estimated levels of signal intensities of PolII at TSS (population size) and TR (transcriptional activity) by the Poisson regression model. **(F)** Volcano plots of the contrasts (day 3 vs. day 0 after CTX injury) for TR (top) and TSS (bottom). The x-axis represents $\log_2$FC (day 3/day 0), whereas the y-axis represents $-\log_{10}$FDR. Significant changes that satisfy $|\log_2 FC| > 1$ (twofold) and FDR < 0.1 are in red. Genes that have the top 10 *p*-values are labelled. **(G)** Heatmap of TSS and TR of annotated genes with a significant change in the TSS-level as shown in Figure 4H**.**

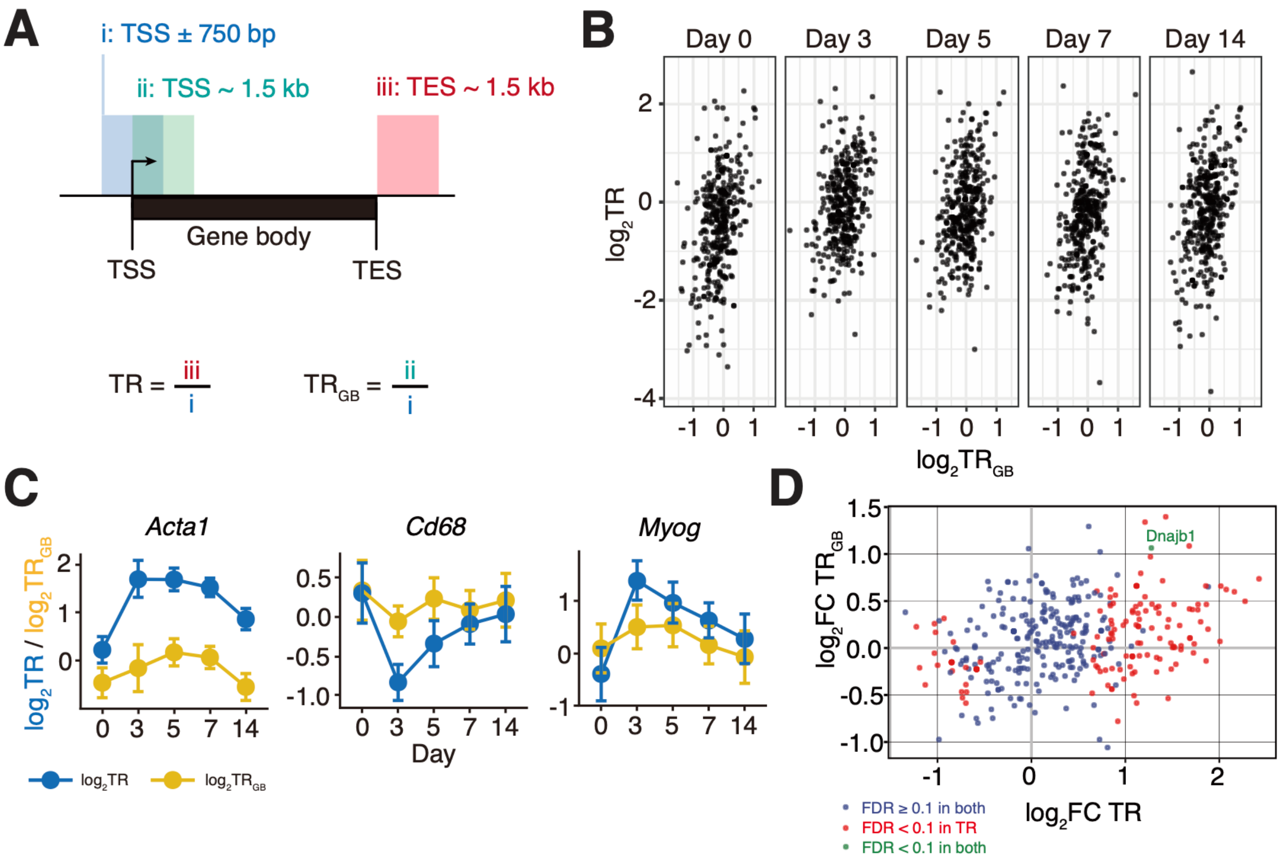


**Appendix Figure S8: The statistical test is sensitive to the definition of the traveling ratio.**

**(A)** Two definitions of the traveling ratio. TR using TES was employed in this study. $TR_{GB}$ is another definition using signals on gene body regions. **(B)** Scatter plots that compare TR and $TR_{GB}$ in the CTX injury data of ChIL PolII S5P. **(C)** Trends of TRs in the time course of the regeneration as shown in Fig. 4E. $TR_{GB}$ is indicated in yellow. Error bars indicate 95% confidence intervals of the estimated TR mean. **(D)** Scatter plot of $log_2FC$ (day 3/day 0) of TR of the annotated genes in De Micheli et al.. The dots are colored in accordance with the detected changes (FDR<0.1) by the Poisson regression model.

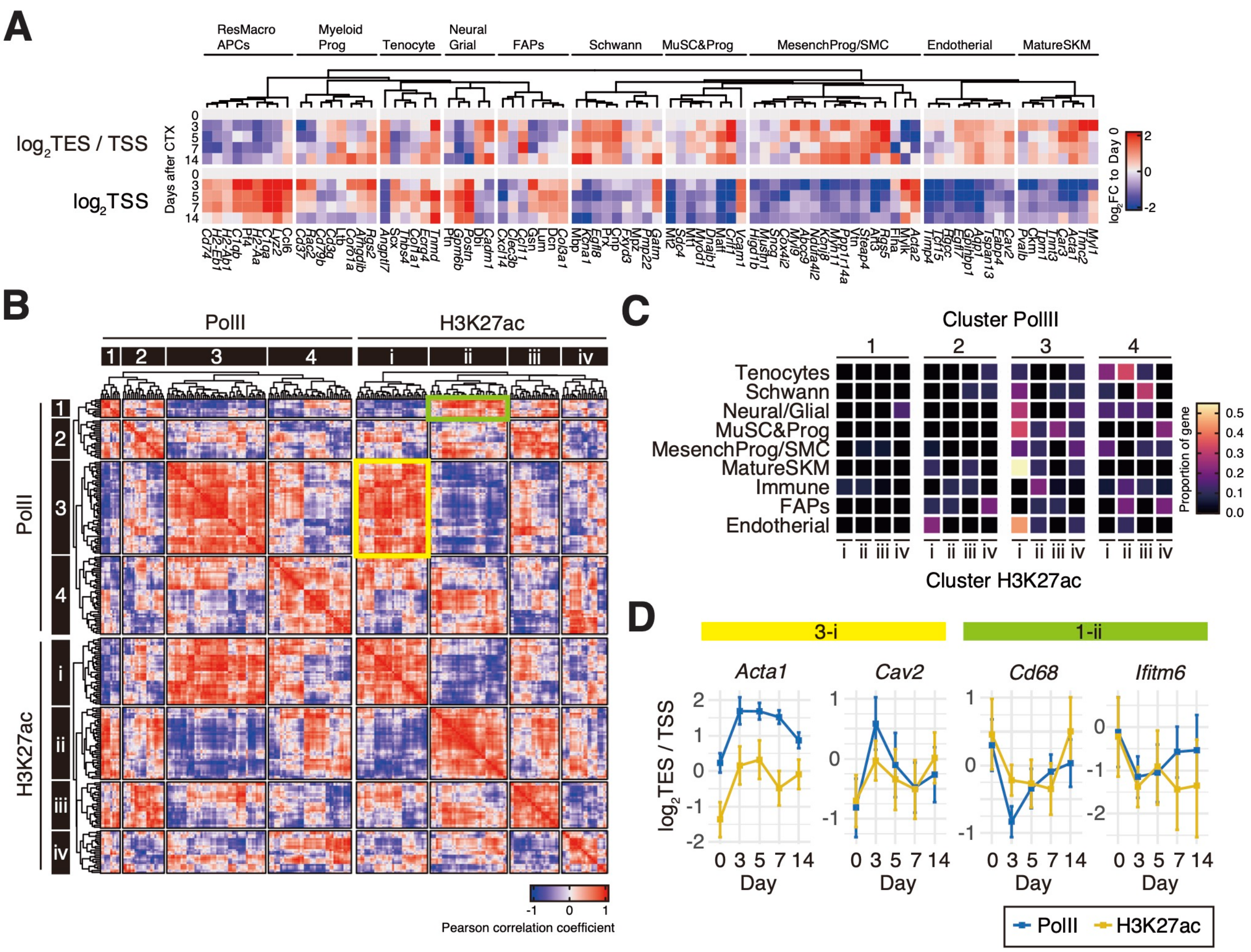

**Appendix Figure S9:** Exploration of chromatin-regulated genes in muscle regeneration.

**(A)** Heatmap of H3K27ac signals in the TSS, and the signal ratio of TES/TSS in the annotated genes with a significant change in TSS level, as shown in **Appendix Figure S7G**. **(B)** Correlation and cross-correlation heatmaps of PolII and H3K27ac distribution at genes. The correlations of TES/TSS in the time course of regeneration (five time points) was evaluated. The matrices of correlation and cross-correlation were stacked in the form of $R_{xx}$, $R_{xy}$ in the first row and $R_{yx}$ $R_{yy}$ in the second row where *x*, *y* indicates PolII and H3K27ac, respectively. **(C)** Proportions of contained genes of each cell type in the cluster are shown. The proportions are calculated as the number of contained genes in the cluster / total genes in the cell types. **(D)** Trends of TES/TSS ratios in the regeneration time course. Genes were selected as representative genes of clusters. Error bars indicate 95% confidence intervals of the estimated mean of TES/TSS.
