# Supplementary material for: Modeling population size independent tissue epigenomes by ChIL‐seq with single thin sections
Source: Mol Syst Biol. 2021 Nov 3;17(11):e10323. doi: 10.15252/msb.202110323 (PMC8564819; doi:10.15252/msb.202110323)
Supplement: Supplementary file 8 [file MSB-17-e10323-s002.pdf]

molecular systems biology

Modeling population size independent tissue epigenomes by ChIL-seq with single thin sections

Kazumitsu Maehara, Kosuke Tomimatsu, Akihito Harada, Kaori Tanaka, Shoko Sato, Megumi Fukuoka, Seiji Okada, Tetsuya Handa, Hitoshi Kurumizaka, Noriko Saitoh, Hiroshi Kimura, and Yasuyuki Ohkawa
DOI: 10.15252/msb.202110323

Corresponding author(s): Yasuyuki Ohkawa (yohkawa@bioreg.kyushu-u.ac.jp)

Review Timeline:

Transfer from Review Commons:	1st Mar 21
Editorial Decision:	15th Mar 21
Revision Received:	7th Jul 21
Editorial Decision:	28th Jul 21
Revision Received:	17th Sep 21
Editorial Decision:	28th Sep 21
Revision Received:	29th Sep 21
Accepted:	1st Oct 21

Editor: Maria Polychronidou

Transaction Report: This manuscript was transferred to MSB following peer review at Review Commons.

(Note: With the exception of the correction of typographical or spelling errors that could be a source of ambiguity, letters and reports are not edited. Depending on transfer agreements, referee reports obtained elsewhere may or may not be included in this compilation. Referee reports are anonymous unless the Referee chooses to sign their reports.)

Thank you for submitting your manuscript to Molecular Systems Biology. I apologize for the delay in getting back to you, which was due to the fact that I was out of office for a few days and it also took some time to consult with an advisor. I have now read the manuscript and your preliminary point-by-point response to the comments of the Review Commons reviewers and discussed them with the team. I have also consulted with a member of our Editorial Advisory Board. As I will explain in further detail below, we would like to invite you to submit a revised manuscript to Molecular Systems Biology.

Overall, we and the EAB member think that tsChIL seems like a potentially relevant approach for epigenomic analyses in tissue samples. However, we agree with the reviewers that as it stands the overall methodological advance remains somewhat unclear and further analyses are required to make a compelling case that tsChIL in combination with statistical modelling can go beyond what is currently possible using other methods, in terms of providing new insights into transcriptional regulation.

We think that the following points are especially important and need to be convincingly addressed in the revision:

- Providing a detailed protocol of how tsChIL works so that the reviewers can evaluate the methodology. We would ask you to use 'Structured Methods', our new Materials and Methods format (see details below), which is helpful for providing methodological details in an accessible way.

- Detailing how tsChIL compares to ChIL-seq, and importantly how it compares to other methods (e.g. MOW-ChIP and Cut&Run) and what are its advantages or unique features. A table listing this information would be helpful for the reviewers.

- Integrating the statistical modelling to the method workflow, as you indicate in your point-by-point response, sounds like a good idea. It is important to clearly describe what the statistical modelling adds to the analysis of transcriptional regulation in heterogeneous tissues. Moreover, it is essential to present evidence that it is broadly applicable and straightforward to adopt by other researchers.

- Several technical concerns, including the need to demonstrate more convincingly that tsChIL requires less reads than RNA-seq should be addressed.

- The addition of the cancer patient data sounds promising as it could potentially showcase the relevance of tsChIL for analyzing clinical samples where the amount of available tissue material tends to be limiting. We understand that it may be difficult to derive conclusive new biological insights from the analysis of these rhabdomyosarcoma samples. However, we feel that it is important to use the application to these cancer samples to make a convincing case that tsChIL presents advantages compared to alternative methods for tissue-based applications.

The eventual acceptance of the study will depend on how well the issues raised by the referees have been addressed. As you might already know, our editorial policy allows in principle a single round of major revision, and therefore it is essential to provide responses to the reviewers' comments that are as complete as possible.

To speed up the evaluation of you revised manuscript we would also ask you to address the following editorial points:

We think that the following points are especially important and need to be convincingly addressed in the revision:

- Providing a detailed protocol of how tsChIL works so that the reviewers can evaluate the methodology. We would ask you to use 'Structured Methods', our new Materials and Methods format (see details below), which is helpful for providing methodological details in an accessible way.

Following your suggestion, we have revised the ChIL protocol in accordance with the "Structured Methods". The modifications of tissue analysis are emphasized with underlines. Please see the materials and methods section.

- Detailing how tsChIL compares to ChIL-seq, and importantly how it compares to other methods (e.g. MOW-ChIP and Cut&Run) and what are its advantages or unique features. A table listing this information would be helpful for the reviewers.

Yes, we agree. We have emphasized and described the following points in detail.
First, the main advantage of our analysis method is that it does not require tissue dissociation. Recently, many studies have demonstrated that disassociation of cells in single cell analysis causes bias. Second, our method enables analysis of the epigenomic status of genes expressed in a small number of cells in a single tissue section from a single thin section. Current single cell analysis requires a large number of cells to start. It is extremely difficult to analyze a small number of rare cells in a human sample. Therefore, we have added a list of comparative methods for our tissue epigenome analysis to new **Table 1** (old Table S1), which include those that were proposed for tissue analysis on the basis of CUT&Tag, Bartosovic et al. (single cell tissue epigenome; Nature Biotech. 2021), and Deng et al. (spatial epigenome analysis; bioRxiv 2021) are additionally mentioned as recent developments in low input/single cell analyses. We have mentioned these techniques in the results section as follows.

[Result]
Low input and single cell level epigenomic analyses have been developed, such as CUT&Run[27] and MOW-ChIP[28], and spatial[29] and single cell level[30] epigenomic analyses have been applied to tissue analysis. However, the dissociation process to obtain a single cell requires a large number of starting cells. Therefore, it is currently difficult to analyze a small amount of starting cells such as those in human clinical samples. The dissociation process may cause a stress-induced bias in gene expression of the tissue[31,32]. For this reason, epigenomic analysis using tissue sections has been widely used.

- Integrating the statistical modelling to the method workflow, as you indicate in your point-by-point response, sounds like a good idea. It is important to clearly describe what the statistical modelling adds to the analysis of transcriptional regulation in heterogeneous tissues. Moreover, it is essential to present evidence that it is broadly applicable and straightforward to adopt by other researchers.

We have demonstrated that the combination of ChIL-Seq, which was optimized for tissues, and statistical models is useful to explore cell type composition and transcriptional activity in tissues upon skeletal muscle injury as a model. This revision has also added new insights into the statistical test for the traveling ratio, which has higher sensitivity when evaluating TR by TES than the gene body, which suggests that the travelling ratio still has potential for various samples to evaluate the epigenome state finely.

In light of the above, we have added the following descriptions about our analysis of transcriptional regulation in heterogeneous tissues.

[Discussion]
We proposed a method for elaborate transcriptional profiling that reflects diverse and unknown cellular compositions in tissues by integrating the statistical model of the traveling ratio with highly sensitive epigenomic analysis of ChIL-seq. RNA-seq can only provide information on an increase or decrease of the total amount of RNA in tissue sections. Conversely, our method provides two types of information simultaneously: the increase or decrease of the cell population in the tissue and the transcriptional activation of genes at high sensitivity.

[Discussion]
Using time series data from a skeletal muscle injury model, we demonstrated that our methodology is useful to assess the heterogeneous population size and transcriptional activity of cell types in tissues. We could also associate marker genes with a particular cell type. As is widely conducted in the field of gene expression analysis, ChIL-seq enables comprehensive screening of transcriptional state changes of all genes. Furthermore, in the analysis of breast cancer tissues, we showed the potential application of detecting transcriptional activity of a minor cell population, which tends to be unclear in RNA-seq.

[Discussion]
TR was previously evaluated by the ratio of the signal around the gene body to the promoter. However, we found that using the TES/TSS ratio, which has the same definition as the analysis of Bartman et al., is more sensitive for the statistical test of the traveling ratio.

We have also added the following text to describe the statistical model.

[Result; after Figure 4D]

To explain the model intuitively, we further demonstrated our TR modeling with artificial data (**Appendix Fig. S6C-E**). The model transformed a pair of (TSS and TES) count values at each locus into TSS (TR=TES/TSS). By fitting the PolII count data to this model, the TSS and TR provides the interpretation of the cell population size with an active gene and the degree of transcription activation of the genes. Furthermore, between-sample normalization by employing the offset term of the total number of reads maintains the information of the sample size of count data (i.e., a large count is more reliable than small counts).

[Discussion]

Our proposed statistical modeling of the traveling ratio can be carried out using a generalized linear model with an offset term in any statistics software. It requires input data of read counts for TSS and TES and the total number of reads and does not require any specialized analysis software. An implementation example in R is shown in our Github page.

- Several technical concerns, including the need to demonstrate more convincingly that tsChIL requires less reads than RNA-seq should be addressed.

We performed the analysis to compare the number of detected genes in the same number of reads as proposed by referee #1 comment #4 and confirmed that more genes were detected by ChIL with fewer reads than RNA-seq. The results were added to new Fig. S5.

- The addition of the cancer patient data sounds promising as it could potentially showcase the relevance of tsChIL for analyzing clinical samples where the amount of available tissue material tends to be limiting. We understand that it may be difficult to derive conclusive new biological insights from the analysis of these rhabdomyosarcoma samples. However, we feel that it is important to use the application to these cancer samples to make a convincing case that tsChIL presents advantages compared to alternative methods for tissue-based applications.

We agree with your point and we would like to show that ChIL can be applied to analyze cancer. We have added data for breast cancer, which may be of great interest to general readers. Because we obtained clinical samples of cancer and normal tissue, we performed analyses by ChIL and RNA-seq on the same tissues.

As shown in the muscle tissue of mice, the PolII signal around TSS provided similar results as the RNA-seq data. Additionally, we succeeded in estimating TR using the same model, even with N=1.

We also found that TR provided some interesting results, which showed different patterns from those of RNA-seq and TSS, and contained genes that were activated in some breast cancers. The results suggested that TR captured a kind of cellular state in breast cancer, which was not detected by RNA-seq. However, we have avoided discussing the biological significance because it requires further verification by obtaining a large number of samples for clinical studies.

Please see new Fig. 5 and the last section of the results.

The eventual acceptance of the study will depend on how well the issues raised by the referees have been addressed. As you might already know, our editorial policy allows in principle a single round of major revision, and therefore it is essential to provide responses to the reviewers' comments that are as complete as possible.

Rev_Com_number: RC-2020-00572
New_manu_number: MSB-2021-10323
Corr_author: Ohkawa
Title: Modeling population size independent tissue epigenomes by ChIL-seq with single-thin sections

Reviewer #1 (Evidence, reproducibility and clarity (Required)):
In the manuscript, Maehara et al. describe a new method called tsChIL-seq. It is developed based on a published technique, ChIL-seq, for visualizing the distribution of target molecules and profiling epigenetic feature at tissue level. Using this new method, the authors claim that epigenomic analysis can be conducted without cell/nucleus extraction from tissue, which may alter the epigenetic feature of the cells. The authors firstly demonstrated that tsChIL can be used to visualize the proteins of interest at subcellular level and presented how H3K27ac and RNA polymerase II distribute among cells in three types of tissues: liver, heart, and testis. Then, the authors utilized the technique to generate whole genome-wide data on H3K27ac and RNA Pol II-S5P binding for all the three tissues and characterized the sensitivity and accuracy of this purposed framework. Furthermore, the authors identified the transcriptional dynamics during the process of skeletal muscle regeneration using tsChIL-RNAPII data and single-cell RNA-seq data by the combining tsChIL-seq and an established statistical model. Overall, the data and method can be potentially interesting. However, as a method paper, their technical details are very sketchy and confusing. The authors didn't show adequate information about the optimization of ChIL-seq and some key steps in the tsChIP-seq, such as tissue immobilization, permeabilization and immunostaining. It is very hard to evaluate whether the method is novel or reproducible. Thus I cannot recommend the publication of the paper in its current form.

We apologize for the lack of rationale for some modifications to our original ChIL protocol. We have updated Figure 1A to highlight successful application of ChIL using tissue samples and added the detailed methods of the protocol of ChIL for tissue.

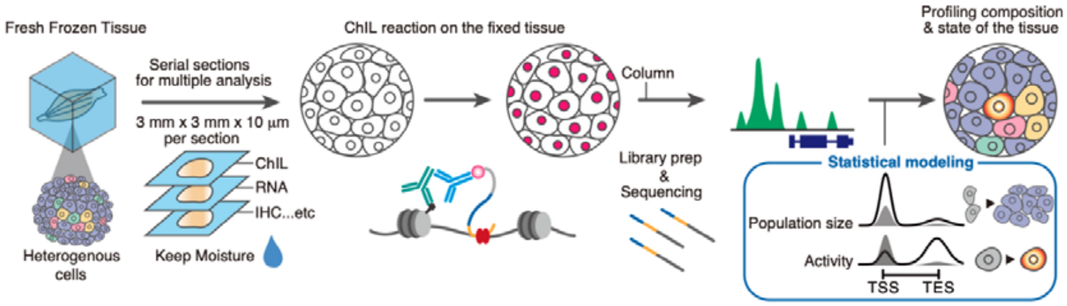

We also avoid using the term "tsChIL-seq" that can be easily misunderstood when referring to a new experimental technique. We have focused on modeling tissue epigenomes by leveraging the various merits of the dissociation-free and low input requirements of ChIL, which were not fully used in our original study.

Please see our point-by-point responses for details.

Major questions:

1. The authors failed to provide enough information about the tsChIL-seq and the details in the steps. The paper as is, is impossible to reproduce by other labs. TsChIL-seq, as a method stemmed from ChIL-seq, was designed for epigenomic analysis at tissue-level. It is quite different from the original version, ChIL-seq, which was designed for cell/nuclei processing. The authors should present more details about how to conduct tsChIL-seq without isolating cells from tissue. In the "Materials and Methods" section, the authors only showed that the incubation time needs to be extended for several steps in tsChIL-seq but didn't explain the rationale behind these changes and how these changes would improve the results.

We apologize that we did not properly explain the rationale of our method and there may some misunderstanding. The original ChIL-seq does not require isolation of cells/nuclei.

However, as this reviewer pointed out, some changes to the experimental conditions that differed from the original were indeed made because we used tissues sections. Our ChIL-seq technology is a technique based on immunostaining. The advantage of immunostaining is that specific signals are obtained on solid-phase carriers relatively easier than reactions in solution, such as immunoprecipitation. It also allows highly sensitive analysis without isolating cells. However, ChIL-seq with tissue sections required a different approach compared with ChIL-seq of fixed cells as reported previously, because ChIL-seq with tissue sections did not work by following a conventional immunostaining protocol for immunohistochemistry.

Therefore, we made changes to ChIL-seq for tissue sections. First, we keep the tissue sections moist and avoided the air drying and/or antigen activation treatment of tissue sections in conventional tissue immunostaining. Next, we optimized the ChIL reaction conditions by modifying ChIL-RNA amplification with IVT reactions and the ChIL-RNA purification steps to eliminate noise of the non-DNA origin of the cells in the tissue produced after the ChIL reaction.

We have updated these points in the results and the detailed method of ChIL for tissue. Please see our updated methods section. We have also emphasized the modifications for tissue.

2. In page 3, line 56 to 57, the authors indicated that it is hard to profile epigenetic features using ChIP-seq based technology with low input cell number. There is very little acknowledgement of previous works in the field. At a minimum, the authors need to compare their approach with existing low-input technologies such as MOWChIP and Cut&run.

We were somewhat careless about the wording. We intended to discuss low input analysis using tissue sections. Additionally, we have already compared low input and single cell chromatin analysis methods, which included MOWChIP and Cut&RUN, in our previous studies of ChIL (reported in Nature Cell Biology and Nature Protocols). In the revised manuscript, we calculated the same performance metrics for our data (e.g., precision, recall, and FRiP) and referred to the performance of MOWChIP and Cut&RUN, although a direct comparison is difficult because the starting materials and number of cells are different.

Therefore, we calculated the same performance metrics as those in our original ChIL-seq study and compared them with the performance of ChIL-seq for cells. We believe these results are useful for comparison with existing methods such as MOWChIP and Cut&RUN.

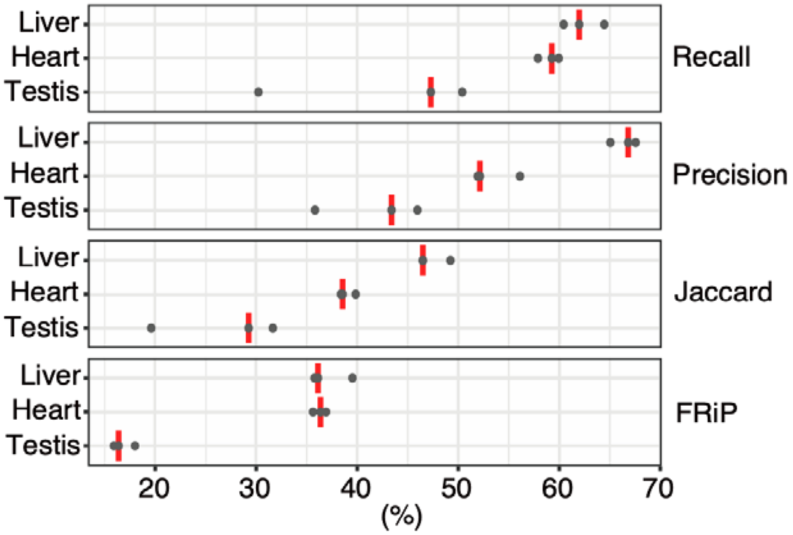

Analysis of the quality of tissue ChIL has been reflected in the text as follows.

[Result]

First, we evaluated the agreement of the ChIL-seq peaks with the reference ChIP-seq peaks as we performed in previous reports[7,8]. ChIP-seq data from ENCODE Bing Ren's data[3] were used as the gold standard reference for ChIP-seq peaks in tissues (**Appendix Fig. S3A–B**). Recall is coverage of the reference ChIP-seq peaks and precision is the proportion of true positives in query ChIL-seq peaks. Recall was 58%–60% for the heart, 65%–68% for the liver, and 30%–50% for the testis, and precision was 52%–56% for the heart, 65%–68% for the liver, and 36%–46% for the testis. We also calculated the Jaccard index that is an integrated score of both recall and precision (heart: 38%–40%; liver: 46%–49%; testis: 20%–29%). Although it was difficult to make a direct comparison of the values because of the different experimental conditions and cell types, the ChIL results in tissues were comparable with our original H3K27ac ChIL of C2C12 cells (30%–40% for 100–100,000 cells). Additionally, in accordance with the ENCODE standard for ATAC-seq peaks (acceptable for FRiP > 0.2, recommended for > 0.3), the liver and heart were met these criteria. Conversely, the testis tended to have relatively low agreement with the reference ChIP-seq peaks. This may be because of the more diverse composition of cells in testis tissue, especially compared with liver tissue that mostly consists of homogeneous hepatocytes.

We had provided a summary table of previous attempts of epigenome analysis of tissue sections in Table S1 of the initial manuscript. In this revision, we have updated the attempts of low input and single cell epigenome analyses of tissues and summarized them together with the characteristics of each method in new Table 1.

3. In page 7, line 196, Sox5 doesn't seem to be the binding motif enriched in the testis-H3K27ac signal, according to the figure 2C. The authors may want to provide quantitative details to show that Sox5 is more enriched than Gabpa or CREB1.

Figure 2C shows the top 10 motifs of gain (bias corrected deviation of chromVAR) from each tissue within the motifs that meet the criterion of deviation Z of >1.96 (i.e. considering $p < 0.05$). Sox5 meets this criterion in the testis (2.90–3.48 in Dataset EV4; old Table S5). To provide more quantitative details of the results, in addition to the current EV4, we have prepared a table of the number of peaks that match the motifs as shown below.

	TotalOL motifs	Liver-m1	Liver-m2	Liver-m3	LV-m1	LV-m2	LV-m3	Testis-m1	Testis-m2	Testis-m3
**TotalOL peaks**		45706	47278	47116	25161	24738	23675	17752	15129	17880
**MA0087.1_Sox5**	3998	2595	2736	2718	1703	1642	1610	1363	1210	1333
**MA0062.2_Gabpa**	3267	2731	2794	2763	1639	1531	1467	1125	889	1225
**MA0018.2_CREB1**	903	724	750	748	441	424	418	315	269	352
**MA0007.3_Ar**	2952	1930	2036	2000	1189	1176	1085	1068	950	1071

(a part of new Extended Dataset EV5)

This table shows the number of motifs that matched the ChIL H3K27ac peak used in the chromVAR analysis. On the basis of the number of used motifs alone, Sox5 was comparable with Gabpa, which had the second highest deviation Z score in the testis. The coverage of Sox5 was relatively low (30%; ~1,300/4,000) compared with other tissues. However, the higher percentage of Sox5 among all H3K27ac peaks (~1,300/17,000 = ~10%) placed Sox5 relatively high (11th) in terms of the deviation Z score.

4. In page 8, line 259-260, the authors claimed that tsChIL RNAP2 requires fewer reads than RNA-seq. However, there is no evidence in this manuscript to support this statement. In order to make it more convincing, the authors may want to provide some data here, like with the same amount of detected genes, the number of reads tsChIL RNAP2 used is smaller than the number of reads required for RNA-seq.

We agree that Fig. 3A provided rather indirect evidence of the requirement for fewer reads in ChIL. To make it more convincing, we analyzed the number of detected genes and the number of reads used in RNA-seq and RNAP2 ChIL in new Figure S5A.

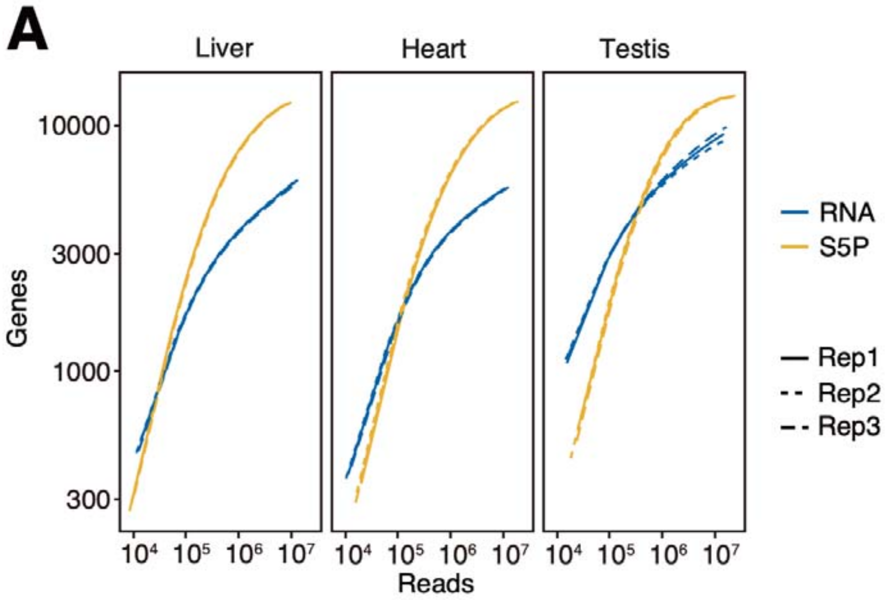

We have added the results of this evaluation to the text as follows.

[Result]

We further calculated the expected number of detected genes in the subsamples of reads (RNA-seq and PolII S5P ChIL-seq) based on Heck et al.. In the case of ChIL-seq, a gene was considered detected if the read count was >0 in the gene region ±1 kb. As a result, the number of detected genes of both ChIL-seq and RNA-seq were close to each other at 1×10^4–10^6 reads, and thereafter in all tissues, showed higher numbers in PolII S5P ChIL-seq (**Appendix Fig. S5A**). These results indicate that PolII S5P ChIL-seq detects more genes than RNA-seq when the number of reads is more than 1×10^5.

Minor questions:

1. In page 4, line 110, the size of input is in conflict with the information provided in fig.1A. The authors need to clarify which number is correct.

The correct size is 3 mm × 3 mm × 10 µm. We have corrected this in new Fig. 1A.

3. In page 7, line 225, the authors used "TEs" in the statement but never mentioned what "TEs" stands for before. The authors need to clarify.

We have avoided the abbreviation because TE was used once in our manuscript.

Reviewer #1 (Significance (Required)):

The method could be interesting. However, the technical detail provided was insufficent for assessment. It is impossible to evaluate the reproducibility for the same reasons.

Reviewer #2 (Evidence, reproducibility and clarity (Required)):
Maehara et al. present a method, tsChIL, based on their prior work ChIL-seq to profile histone modification (H3K27ac) and RNAPII on tissue sections. They first demonstrated their approach by profiling H3K27ac and RNAPII in three tissues (heart, lung and testis) and also examined the correlation between RNAPII and transcriptional dynamics in a biological system of skeletal muscle regeneration by modeling RNAPII traveling combining with published scRNA-seq datasets. However, given that many low-input ChIP-seq methods have been developed for single cells or tissue sections, the methodological advance is not clear here, not to mention the trivial improvement of this work from their previous tsChIL-seq. Further, the second part related to modeling RNAPII travelling did not obtain new findings in terms of transcriptional regulation.

We appreciate your comments on the manuscript. Both referees asserted that there was a lack of clarity in the methodological advances of our method. We have therefore revised the manuscript to better demonstrate the novelty of the method.

Please see our point-by-point responses below.

Specific main concerns:
1. The RNAPII-S5P is known to enrich in promoter-proximal regions, while RNAPII-S2P predominates in regions that are more distal from the promoter during transcription elongation (PMID: 11018013). However, read distribution of the RNAPII-S5P ChIL in Fig.1G exhibited a very small fraction of reads mapped to promoter regions. That needs more clarification.

The anti-PolII S5P antibody that we used is established in our previous study (Odawara et al.) and we found that the signal was dominantly enriched in the promoter-proximal region, which consistent with the paper the reviewer mentioned (PMID: 11018013) and it also reacts to S5PS2P as well.

To confirm this point, we further analyzed PolII-S2PS5P in addition to the S5P data and then compared the relative coverages of promoter-proximal and gene body regions. The data showed that the S5P is more enriched in the promoter region compared to S2PS5P.

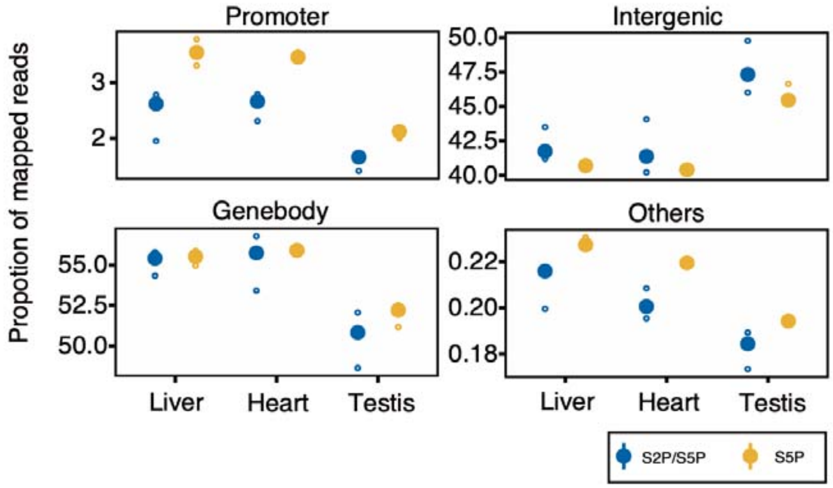

We added the result to the text as follows.

[Result]

The PolII S5P antibody that we used detects both S5P alone and S5PS2P in the C-terminal domain (CTD) of PolII. PolII is known to switch from promoter-proximal pausing state to elongation state by phosphorylation of Ser2 in addition to Ser5 in the C-terminal domain (CTD). In **Appendix Figure S2**, we compared the proportion of mapped reads in the same genomic region in Figure 1G and found that the S5P was a higher proportion than that in the S2PS5P in the gene promoters. These results suggest that ChIL with S5P and S5PS2P antibodies reflect the state of transcription in response to CTD phosphorylation of PolII.

2. The authors performed analyses such as library complexity and correlation analysis to validate the data quality of tsChIL in Fig. 1, but global evaluation of signal-to-noise ratio is lacking. It would be good to see the peak numbers, peaks overlapped with those from conventional bulk ChIP, etc...

We have evaluated the performance metrics as suggested and reflected this in new Figure S3A. Although the objective evaluation of the performance using called peaks is difficult because of the arbitrary setting of thresholds, we instead evaluated the ROC and PR curves that evaluate the performances at variable thresholds (Fig. S3B).

The quality assessments of tissue ChIL have been reflected in the text. Please refer to new Fig. S3 and the response to comment 2 of referee #1 for details.

3. In Fig. 1H, the correlation analysis shows that liver and heart clustered together based on genome-wide 10 kb-binned H3K27ac signal. However, the motif enrichment analysis in Fig. 2C based on H3K27ac signal in peaks shows that regulatory TF of liver is more specific, while the pattern of TF motif score in heart and testis is quite similar. What if using the signal in bins as input for chromVAR? I am concerned about the potential noise during analyses.

Because the chromVAR analysis was based on the signal of the peak and we filtered irreproducible peaks, it is possible for somewhat different results to be presented in Figs. 1H and 2C. Indeed, the genome-wide correlation evaluated signals at both on- and off-peaks. Therefore, the reviewer's point is correct.

However, it is difficult to analyze entire bins in the genome by chromVAR. Hence, we reproduced the signal correlation between the heart and testis as seen in chromVAR using a genome-wide bin. We revealed that the difference came from an out-of-peak region.

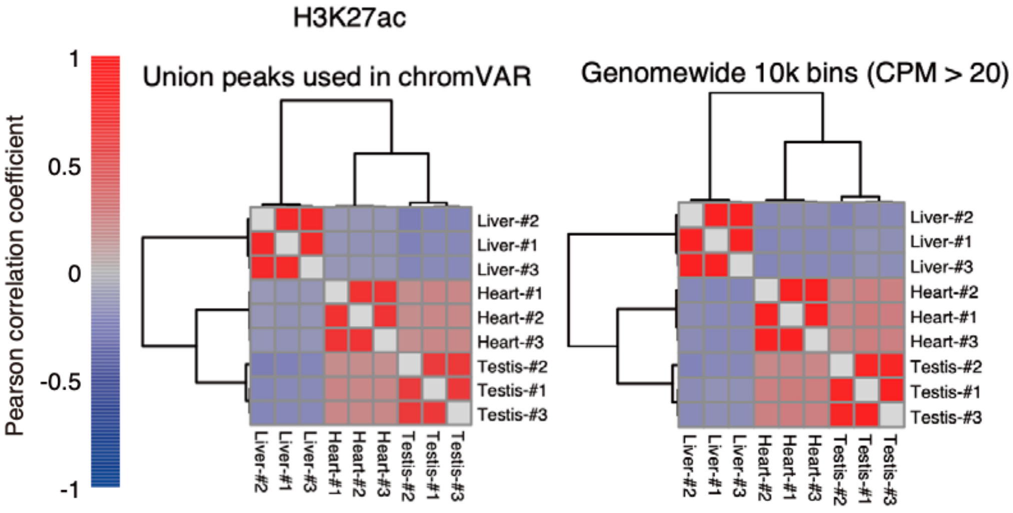

We have discussed this point in the text below.

[Result]

Figure 2C shows the similarity between the testis and heart in the chromVAR analysis and **Figure 1H** shows the similarity between the liver and heart. The difference appeared to be attributable to the chromVAR method of evaluating only signals around peaks. To test this hypothesis, we evaluated signal correlations of ChIL H3K27ac by restricting the signals on peaks (the union of the peaks in the three tissues) (**Appendix Fig. S3C**). As expected, the signals of the heart and testis showed similarity to the chromVAR results. This result was also reproduced using the genome wide 10K bins with strong signals (count per million [CPM] is greater than 20 in any tissue). These results suggest that we need to be aware of the difference between genome-wide correlations, which include potential background signals, and signal correlations on peaks.

4. For analyzing the traveling ratio, please clarify the rationale of region selection for statistical modeling using TSS-region (−750 to +750 bp) and TES-region (0 to +1,500 bp), and why the RNAPII signals at gene body regions are not considered for evaluating the elongation process of transcription.

This is because a recent study by Bartman et al. (Figure S1F in PMID: 30554946), which we refer to in the text, has confirmed that including the gene body region instead of the 3′-end region has less effect on the TR estimation. We therefore compared the results of TR estimation with/without a gene body signal.

Upon this verification, we found that the TR evaluation using the TES region, which allows more reads to be detected, was more favorable for statistical testing, although the two results of the TR are positively correlated.

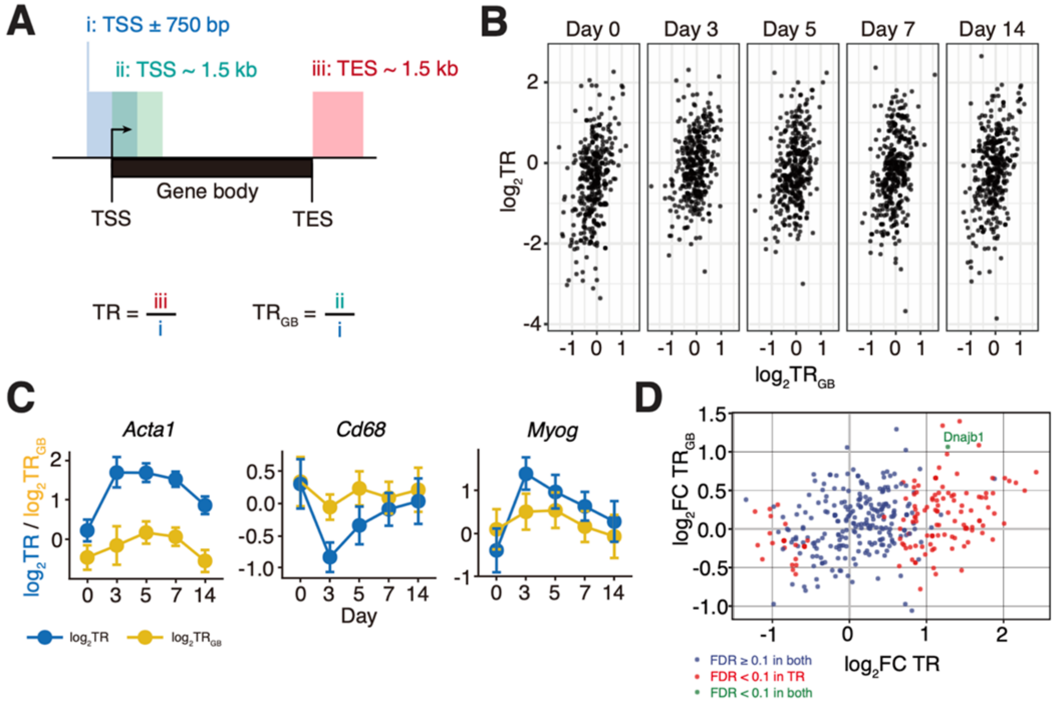

These validation results were added to Figure S7 as above and the text as follows.

[Result]

The definition of TR used in this study (TES/TSS) does not consider the information from gene body (GB) regions (**Appendix Fig. S7A**). We therefore evaluated the effect of changing the definition of TR. We first evaluated Pearson correlation coefficients between our TR and the TR that used GB (TR_{GB}). Days 0, 3, 5, 7, and 14 were 0.39, 0.30, 0.32, 0.33, and 0.39, respectively (**Appendix Fig. S7B**). Both TRs showed positive correlations, but TR_{GB} showed a rather mild change in TR, whereas our definition of TR (TES/TSS) showed a relatively pronounced change in TR on day 3 (**Appendix Fig. S7C**). To verify this observation, we tested the TR changes of day 0 vs. day 3 after muscle injury. As a result, we found no significant (FDR<0.1) changes in TR_{GB}, except for *Dnajb1* (**Appendix Fig. S7D**). This indicates that TR estimation using the TES region is favorable for statistical evaluation of changes in the transcriptional state.

5. In Fig. 4H, the authors selected 66 genes with TR change (FDR < 0.1) among different timepoints, but only part of the genes were shown in the heatmap. Furthermore, there are only few genes exhibiting decreasing trends of TR after injury. To make clear how cell number and TR harbor the activated genes for each cell type, it may be worth having an additional figure to visualize genes upon significant changes in TSS-level, then to see the dynamic pattern of TR.

We have only shown genes mapped to scRNA-seq annotations for validation.
We agree that showing a heatmap of genes with significant TSS changes will help to better understand the trend of the cell number variation of each cell type used in this annotation. As suggested, we created a figure similar to Fig. 4H for the 83 genes with significant changes (FDR<0.1) in TSS.

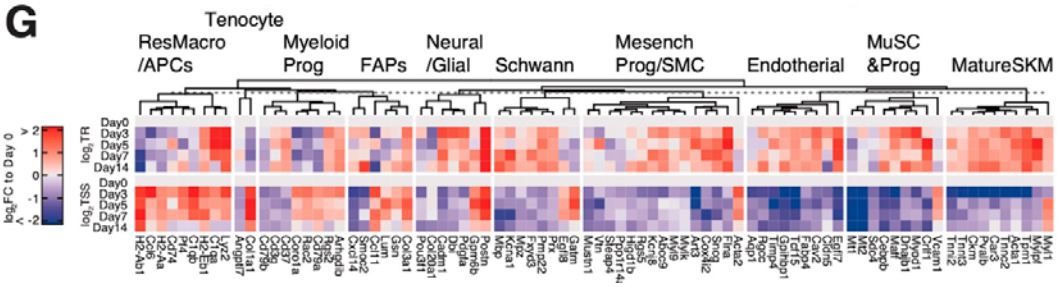

This result has been added just before Fig. 4H as shown below.

[Result]

First, to assess changes in the number of cells in the tissue after muscle injury, we created a heatmap that showed the changes in the TSS level of all 83 genes used in this cell type annotation (**Appendix Fig. S6G**). In particular, the number of cells in macrophages showed an increasing trend in response to inflammation, and myeloid progenitors and FAP showed a slightly elevated trend. Conversely, the numbers of cells in Schwann, mesenchymal progenitor/smooth muscle cells, MuSCs & progenitors, and mature SKMs were decreased from the preinjury level, whereas many genes showed an increasing TR trend.

6. The exploration of dynamics between population and transcriptional regulation using statistical modeling is interesting. However, this analysis largely relies on the well-defined annotation and differentially expressed genes among different cell types derived from existing scRNA-seq datasets, which hardly provides new biological insights. Moreover, to validate the power of this method, I think that visualization and analysis of ChIL signal in serial-thin tissue sections is supposed to be performed as an important application to dissect the regulatory mechanism at spatio-temporal resolution in a complex biological system.

We have performed the validations using the cell type annotation derived from scRNA-seq. However, this does not mean that scRNA-seq is essential but serves as an example of how to

link genes and cell types. The case of one-too-many correspondences between genes and cell types was also discussed as a possible future development. In this study, we used scRNA-seq data to validate changes in the transcriptional activity of each cell type. We have demonstrated the validity of our method but limited this work to the scope of proof-of-principle.

We have therefore added data in the revised version by analyzing the transcriptional profile in cancer tissues using RNAPII ChIL (please see our new Fig. 5), where the cellular composition is diverse among samples, which makes it difficult to obtain single cell data or annotations. Similar to the results shown in Fig. 3, the gene expression profile of RNAPII in cancer tissues correlated with the results of RNA-seq in the same tissues. Furthermore, RNAPII provided information on transcriptional activation through evaluation of TR. We believe that the results of this analysis provide an illustrative example of transcriptional profiling of tissues of unknown composition combined with statistical modeling of RNAPII ChIL.

Minor points:

1. Several typos exist in the manuscript. For example, In the "Tissue preparation" of methods, "mise" should be "mice".

We have fixed the typos in the revised manuscript.

2. The abbreviations should be unified throughout the manuscript. For example, "Pol2", "PolII", and "PolIIS5P".

We fixed the issue in the revised manuscript.

Reviewer #2 (Significance (Required)):

The authors present an improved method based on their prior technique ChIL-seq to work on tissue sections. This would be one of many similar methods developed in the field. The authors nicely demonstrated their usefulness in some cases although the entire procedure appeared rather complex. Researcher interested in tissue ChIP-seq may want to give it try.

I have expertise on single-cell multiomics technologies, and epigenetic regulation of gene transcription in the field of developmental biology.

Referees cross-commenting

Overall, I agree with Reviewer #1 that insufficient details as to how the tsChIL-seq was optimized from the original ChIL-seq are provided in the methods.

Thank you again for submitting your revised study to Molecular Systems Biology along with the referee reports from Review Commons. We have now heard back from the two reviewers who were asked to evaluate your revised study. As you will see below, the reviewers think that the study has improved as a result of the performed revisions. However, reviewer #2 still lists some remaining concerns, which we would ask you to address in a revision. We have also consulted with reviewer #1, as part of our cross-commenting process in which all reviewers are given the chance to comment on each other's reports. Reviewer #1 mentioned that they agree with the comments of reviewer #2 and that it should be possible to address them in an additional round of revision.

On a more editorial level, we would ask you to address some remaining issues listed below:

REFEREE REPORTS

Reviewer #1:

The authors have addressed the concerns satisfactorily. I can recommend the publication.

Reviewer #2:

In this revision, the authors have addressed most of my concerns. A number of clarifications and new data about the application of tsChIL-seq were added, and this manuscript has been improved. However, major concerns still remain.

1. Since similar methods have been developed to profile histone modification starting from low input even single cells, it is essential to make a full comparison with known technologies. The authors have listed some epigenomic methods in Table 1, but the comparison of data quality for different methods is still lacking. I hardly agree with that "a direct comparison is difficult because the starting materials and number of cells are different" from the authors' response to the point 2 of Reviewer #1. It is pretty common to make comparison of signal-to-noise ratios across these methods with varying cell numbers, which is undoubtedly important and helpful for evaluating ChIL-seq data in tissue.
2. The authors performed correlation analysis for different replicates in three tissues using chromVAR TF scores in union peaks and H3K27ac signals in genomewide 10k bins (CPM > 20), and found that liver was negatively correlated with heart and testis in Figure S3C. However, the correlation between liver and heart/testis is ~0.7 in Figure 1H. If this difference was due to the potential noise, I do not think that the data with such noise could be used to uncover true biological signals.
3. Based on PolII ChIL-seq data, the authors provided a statistical model to interpret the change of population size and transcriptional activity during dynamic biological process. This can easily be obtained by single-cell RNA-seq analysis, especially when there are no other datasets and applications for this model on other histone marks or epigenetic factors except for PolII.
Therefore, although this manuscript has been improved, I am still concerned about the data quality at this timepoint.

Thank you again for submitting your revised study to Molecular Systems Biology along with the referee reports from Review Commons. We have now heard back from the two reviewers who were asked to evaluate your revised study. As you will see below, the reviewers think that the study has improved as a result of the performed revisions. However, reviewer #2 still lists some remaining concerns, which we would ask you to address in a revision. We have also consulted with reviewer #1, as part of our cross-commenting process in which all reviewers are given the chance to comment on each other's reports. Reviewer #1 mentioned that they agree with the comments of reviewer #2 and that it should be possible to address them in an additional round of revision.

We are very grateful for the chance to improve and resubmit our manuscript. We believe we have appropriately addressed the points raised by you and the reviewers, and have outlined our modifications in the responses below.

On a more editorial level, we would ask you to address some remaining issues listed below:

Reviewer #1:

The authors have addressed the concerns satisfactorily. I can recommend the publication.

We appreciate this comment and are grateful to this reviewer for helping us improve the presentation of our methods.

Reviewer #2:

In this revision, the authors have addressed most of my concerns. A number of clarifications and new data about the application of tsChIL-seq were added, and this manuscript has been improved. However, major concerns still remain.

We appreciate this professional advice. We have performed additional data analysis and include new H3K27ac ChIL-seq data from muscle tissue in response to these comments. We believe this revised version will now be acceptable.

Please see our point-by-point responses below:

1. Since similar methods have been developed to profile histone modification starting from low input even single cells, it is essential to make a full comparison with known technologies. The authors have listed some epigenomic methods in Table 1, but the comparison of data quality for different methods is still lacking. I hardly agree with that "a direct comparison is difficult because the starting materials and number of cells are different" from the authors' response to the point 2 of Reviewer #1. It is pretty common to make comparison of signal-to-noise ratios across these methods with varying cell numbers, which is undoubtedly important and helpful for evaluating ChIL-seq data in tissue.

We agree with this point. Indeed, performance comparisons with the methods listed in Table 1, while keeping in mind differences in the number of tissues and cells (sections) being used, will provide readers with useful guidelines for selecting epigenomic analysis methods for tissues. Therefore, we compared the ROC and FRiP with the epigenomic analysis method for tissue sections listed in Table 1 to comprehensively evaluate the S/N and efficiency of data acquisition. ChIP-seq data for the reference tissues derive from the gold standard ENCODE project, as described in our report (Harada *et al*, 2018; Handa *et al*) and the previous revision. Regarding data selection, we were unable to compare Chrom-EX PE because the FASTQ file was not available. However, it has previously been shown that FiTAc is superior to Chrom-EX PE (Font-Tello *et al*, 2020). Therefore, we have cited this study and omitted the comparison here. We also evaluated H3K4me3 data in the spleen of PAT-ChIP for the comparison of the performance of epigenome analysis in mouse tissue, although it is not directly comparable with our data. The results are shown below.

First, we focused on FiTAc-seq, which is an improved version of FiT-seq focusing on histone acetylation, and is considered to be state-of-the-art in tissue-based epigenomic analysis. FiTAc-seq showed very high agreement and high S/N with ENCODE ChIP-seq data (AUC: 0.96–0.97), even though it targeted FFPE. Our ChIL-seq, using a relatively small number of intercepts, also showed a performance measure comparable with that of FiTAc-seq (AUC: 0.94–0.95). Thus, we proved that ChIL-seq can achieve a sufficiently high performance with fewer cells.

These results will be inserted after the calculation of the performance index for ChIL-seq alone (Figure S3) in the previous revision, as shown below. A detailed table has been added to the new Dataset EV3.

[page 7, line 228]

"Although it was difficult to make a direct comparison of the values because of the different experimental conditions and cell types used, we compared the performance of ChIL-seq with that of other methods for tissue epigenome analysis (**Table 1**). FiTAc-seq (Font-Tello *et al*, 2020) is an improved version of FiT-seq for histone acetylation, and is a state-of-the-art method for the epigenomic analysis of FFPE tissue sections. We evaluated the concordance of FiTAc-seq with ENCODE liver ChIP-seq (**Appendix Figure S3C)**. FiTAc-seq showed very high concordance with ENCODE ChIP-seq data (Recall: 70%, Precision 75%, AUC: 0.96–0.97), even though it was performed on FFPE. PAT-ChIP of spleen H3K4me3, a similar tissue epigenomic analysis method, showed high agreement with ENCODE tissue ChIP-seq data (AUC:~0.9; **Appendix Fig. S3D**). Moreover, our ChIL-seq obtained from a relatively small number of cells (one section; **Table 1**) was also highly comparable with FiTAc-seq data for the same tissue and histone modifications (AUC: 0.94–0.95)."

[new Figure S3C-D and new Dataset EV3 for the detail]

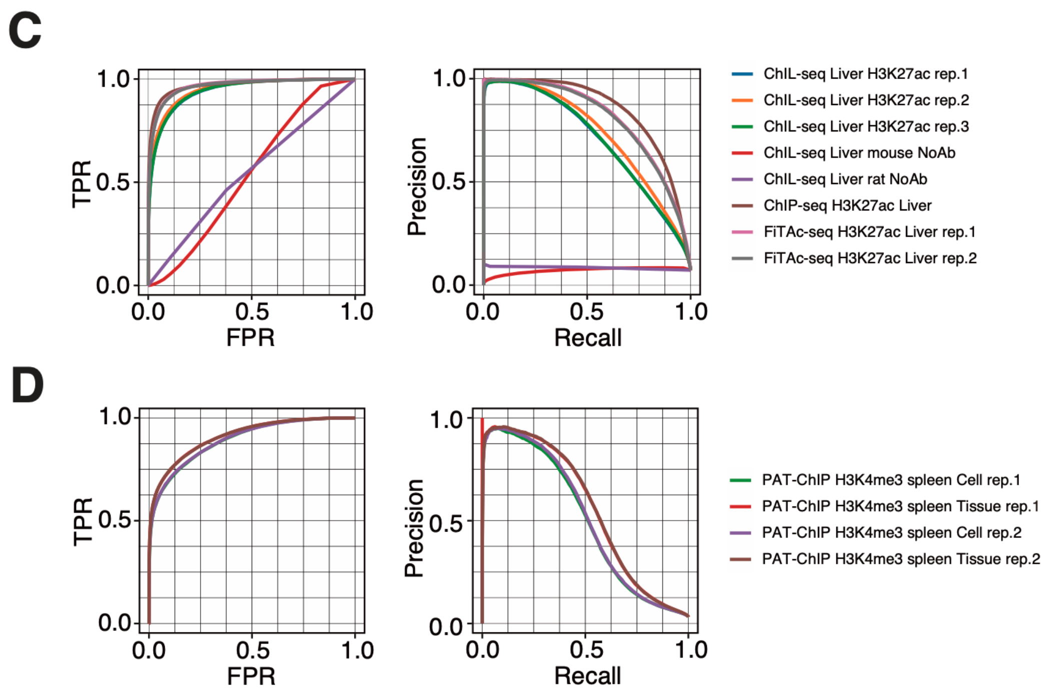

2. The authors performed correlation analysis for different replicates in three tissues using chromVAR TF scores in union peaks and H3K27ac signals in genomewide 10k bins (CPM > 20), and found that liver was negatively correlated with heart and testis in Figure S3C. However, the correlation between liver and heart/testis is ~0.7 in Figure 1H. If this difference was due to the potential noise, I do not think that the data with such noise could be used to uncover true biological signals.

First we would like to note that the calculation of correlation coefficients for genome-wide bins is greatly impacted by bins outside peaks (with no or low signals) which account for the vast majority of the genome.

To further investigate the potential noise referred to by the reviewer, we evaluated the effect on the correlation calculated from bins with low signal, which is generally considered background signal (noise) and was not discussed in the previous revision.

From the data added in the previous revision, cited below, we found that the S/N varies among tissue types.

"Recall was 58%–60% for the heart, 62%–64% for the liver, and 30%–50% for the testis; precision was 52%–56% for the heart, 65%–67% for the liver, and 36%–46% for the testis."

As mentioned in our response to your comment #1 above, liver H3K27ac data have a high S/N, while testis data have a low S/N, which is referred to in the manuscript.

[new Figure S4]

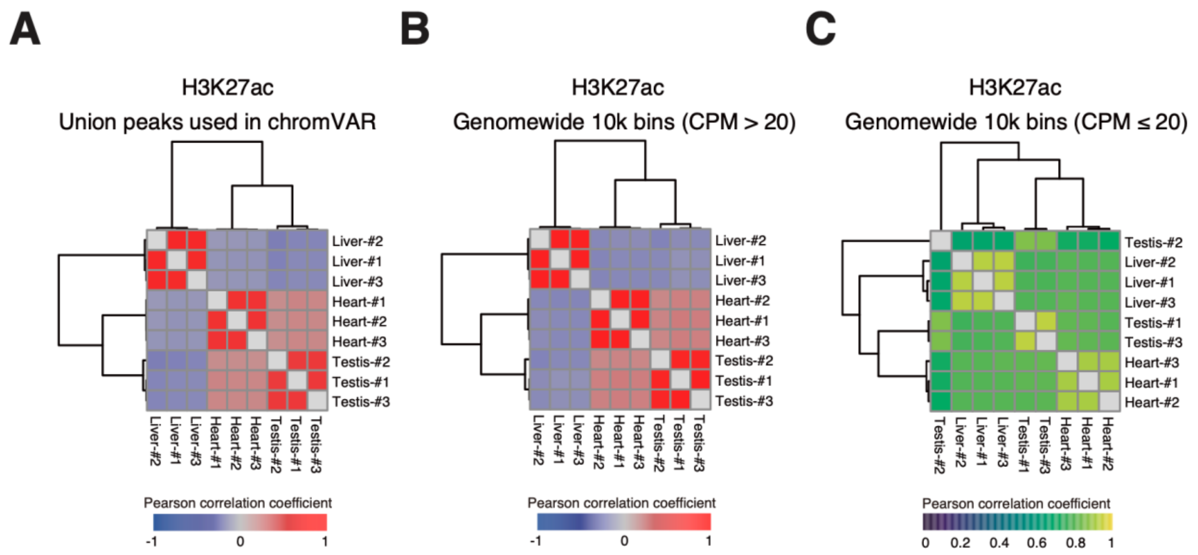

We focused on the testis data, which have a particularly low S/N. We selected nine datasets of H3K27ac from each tissue, and observed that replicate 2 was outside the testis cluster when correlation coefficient matrixes for bins with CPM<20 were used. It therefore appears that the background signal has a relatively strong effect in the testis. This tendency is not observed in liver or heart data. Therefore, it is possible that the fall in S/N depends on the tissue, but we do not think this is a problem in the ChIL method itself.

Even with such low signal-to-noise data, we believe that it is possible to avoid the effect of potential noise and perform a reasonable analysis by focusing on the region with high signal. This can be done by limiting the analysis to the peak region, as shown in our chromVAR example. In fact, the analysis limited to CPM >20, which was added in the previous revision, fits into the same testis cluster and showed a high correlation between replicates.

Based on the results of the above verification, a new Fig. S4 and additional text were added as follows:

[page 9, line 289]

"In the liver data, for example, the bins satisfying CPM >20 account for about 2% of the genome, but for nearly 20% of the total reads (**Appendix Fig. S4C**). Therefore, the liver data show a high accumulation of signal in the peak region. In contrast, testis data showed that bins satisfying CPM >20

accounted for less than 1% of all bins and contained 2%–3% of total reads. The majority (98%–99%) of the genomic regions with CPM ≤ 20 signals are considered to be the main source of genome-wide correlation. This suggests that assessing the peak regions can help cope with the low S/N testis data."

3. Based on PolII ChIL-seq data, the authors provided a statistical model to interpret the change of population size and transcriptional activity during dynamic biological process. This can easily be obtained by single-cell RNA-seq analysis, especially when there are no other datasets and applications for this model on other histone marks or epigenetic factors except for PolII.

Therefore, although this manuscript has been improved, I am still concerned about the data quality at this timepoint.

We believe this is an important point. We have therefore revisited our work, incorporating these suggestions.

First, we agree that scRNA-seq is desirable for profiling the cellular composition of tissues if it can be performed easily and successfully. However, there might be problems such as cost, efficiency in obtaining the cell population of interest, or difficulty in dissociation in some tissues. Also, even if dissociation is performed, it is known to cause bias in gene expression (Machado *et al*, 2021; Miyawaki-Kuwakado *et al*, 2021), and we believe that scRNA-seq is not necessarily always the best option.

Machado L, Relaix F & Mourikis P (2021) Stress relief: emerging methods to mitigate dissociation-induced artefacts. *Trends Cell Biol*: S0962-8924(21)00096–9

Miyawaki-Kuwakado A, Wu Q, Harada A, Tomimatsu K, Fujii T, Maehara K & Ohkawa Y (2021) Transcriptome analysis of gene expression changes upon enzymatic dissociation in skeletal myoblasts. *Genes to Cells* 26: 530–540

Therefore, we focused on transcription activity as another way of profiling the cell composition rather than transcriptome profiling. It should be noted that transcriptional activity is not necessarily the same as gene expression, which is determined by the balance between RNA synthesis and degradation. Therefore, the purpose of this paper is not to propose PolII ChIL-seq as an alternative to scRNA-seq, but to verify the correlation between transcriptional activation and gene expression levels, and then to test the possibility of profiling transcriptional activity in a heterogeneous cell population.

The advantages of employing ChIL-seq for tissue analysis and combining statistical models, as presented in this paper, are summarized in the two points below:

- the ability to perform multiple epigenomic analyses on the same tissue using serial sections
- the ability to quantify the epigenomic state (shape) which is less affected by the size of the cell population in the tissue.

Therefore, we tested the two advantages of ChIL-seq mentioned above by analyzing the epigenome, as suggested by the reviewer. In the additional experiment, we added 25 new ChIL-seq datasets (5 time points × 5 replicates) of H3K27ac in the muscle injury model, and applied the same statistical model as TR to quantify the distribution change of H3K27ac. We confirmed that evaluation of the signal at the TSS is affected by population size, similar to PolII data. As a more advanced application, we cross-referenced epigenomic data from two types of epigenomes in the same tissue to search for genes

where transcription and H3K27ac localization (TES/TSS) are synchronized during muscle regeneration; namely, where the active intervention of chromatin in gene activation is inferred.

With the addition of this analysis, we believe that we have been able to further demonstrate the usefulness of ChIL-seq in tissue profiling. Please refer to the details reflected in the text:

[page 15, line 538]

"The above validation shows that the TR statistical model can be used to analyze epigenomic state changes that are less affected by changes in cell population size in the tissue. Moreover, ChIL-seq has the advantage that multiple epigenomic analyses can be performed on the same tissue using serial sections. As an application of this advantage, we evaluated the H3K27ac data using the same statistical model as used for TR (**Appendix Fig. S9A**). In the case of H3K27ac, TR is not a measure of transcriptional elongation but an indicator of changes in the distribution of histone modifications at a locus, as defined by TES/TSS. As we show in the muscle injury Pol II data, an increased signal around the TSS with increased immune cells was observed, together with a decreased signal in the skeletal muscle and muscle progenitor cells. Next, we searched for genes in which histone acetylation is actively involved in transcriptional regulation to relate the biological significance to the measure of distributional change (TES/TSS) at the locus. For each time point, we clustered each Pol II and H3K27ac data using the TES/TSS index estimated from five replicates (**Appendix Fig. S9B**). We obtained four clusters with correlated signal distribution changes. By calculating the cross-correlation between them (H3K27ac vs. PolII), we identified 3-i as the largest cluster among those whose signal distribution changes were synchronized between H3K27ac and PolII. Cluster 3-i contained many genes expressed in epithelial and skeletal muscle and skeletal muscle progenitor cells (**Appendix Fig. S9C, D**). We also identified cluster 1-ii, in which H3K27ac and PolII were synchronized but showed a different temporal pattern than cluster 3-i. Cluster 1-ii contained genes representative of immune cells. These results suggest the active involvement of chromatin in gene activation during muscle regeneration and demonstrate the advantages of ChIL-seq for performing epigenomic analysis in the same tissue with multiple types of epigenomes."

[new Figure S9]

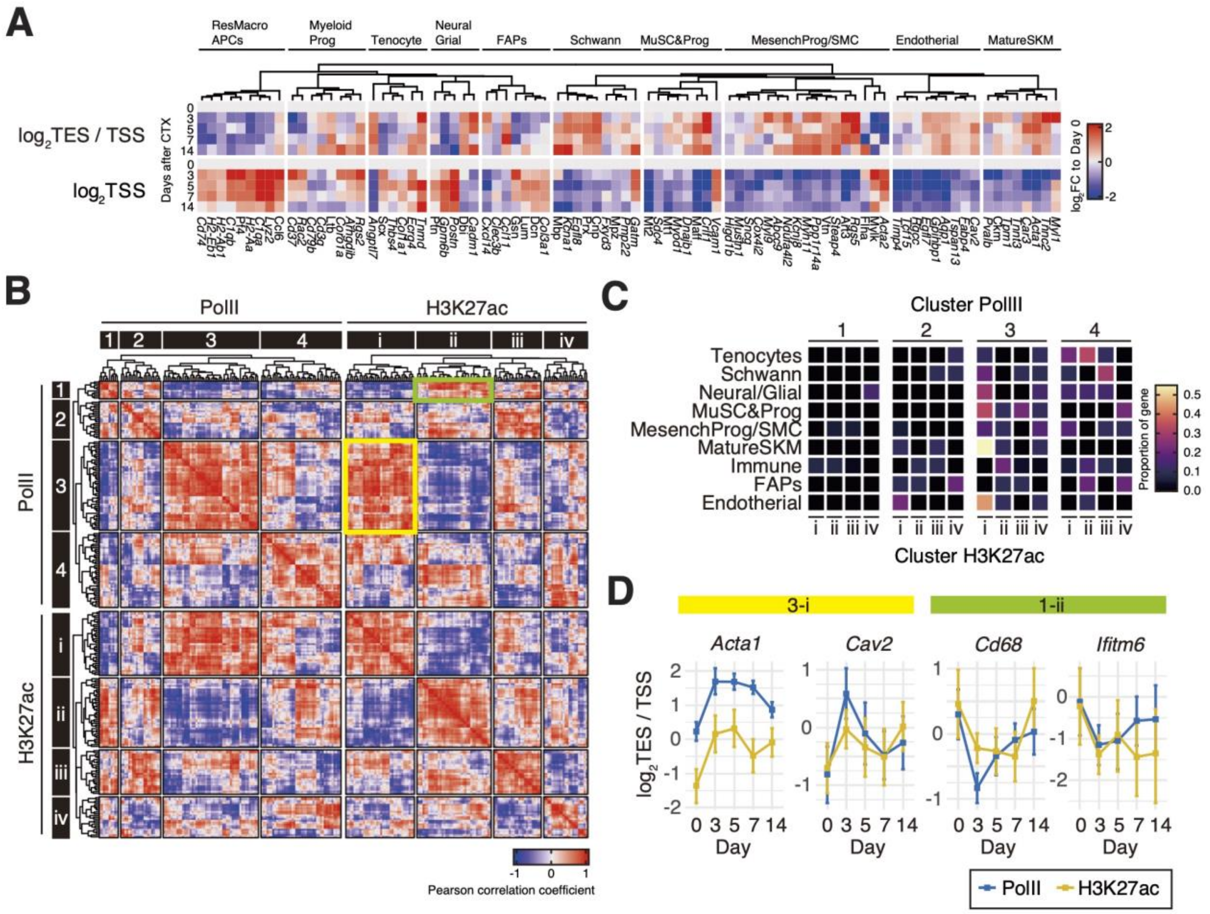

"**Appendix Figure S9**: Exploration of chromatin-regulated genes in muscle regeneration. **(A)** Heatmap of H3K27ac signals in the TSS, and the signal ratio of TES/TSS in the annotated genes with a significant change in TSS level, as shown in Figure S7G. **(B)** Correlation and cross-correlation heatmaps of PolII and H3K27ac distribution at genes. The correlations of TES/TSS in the time course of regeneration (five time points) was evaluated. The matrices of correlation and cross-correlation were stacked in the form of R_{xx}, R_{xy} in the first row and R_{yx} R_{yy} in the second row where x, y indicates PolII and H3K27ac, respectively. **(C)** Proportions of contained genes of each cell type in the cluster are shown. The proportions are calculated as the number of contained genes in the cluster / total genes in the cell types. **(D)** Trends of TES/TSS ratios in the regeneration time course. Genes were selected as representative genes of clusters. Error bars indicate 95% confidence intervals of the estimated mean of TES/TSS."

2nd Revision - Editorial Decision 28th Sep 2021

Thank you again for submitting your revised study to Molecular Systems Biology. We have now evaluated the revised manuscript and we think that the performed revisions have satisfactorily addressed the remaining issues raised by the reviewers. As such, I am glad to inform you that the study is now in principle suitable for publication in Molecular Systems Biology.

Before we can formally accept the study for publication, we would ask you to address some remaining editorial issues listed below.

3rd Authors' Response to Reviewers 29th Sep 2021

The authors have made all requested editorial changes.

Thank you again for sending us your revised manuscript. We are now satisfied with the modifications made and I am pleased to inform you that your paper has been accepted for publication.

EMBO PRESS

YOU MUST COMPLETE ALL CELLS WITH A PINK BACKGROUND ↓

PLEASE NOTE THAT THIS CHECKLIST WILL BE PUBLISHED ALONGSIDE YOUR PAPER

Corresponding Author Name: Yasuyuki Ohkawa
Journal Submitted to: Molecular Systems Biology
Manuscript Number: MSB-2021-10323

Reporting Checklist For Life Sciences Articles (Rev. June 2017)

This checklist is used to ensure good reporting standards and to improve the reproducibility of published results. These guidelines are consistent with the Principles and Guidelines for Reporting Preclinical Research issued by the NIH in 2014. Please follow the journal's authorship guidelines in preparing your manuscript.

A- Figures
1. Data
The data shown in figures should satisfy the following conditions:

- the data were obtained and processed according to the field's best practice and are presented to reflect the results of the experiments in an accurate and unbiased manner.
- figure panels include only data points, measurements or observations that can be compared to each other in a scientifically meaningful way.
- graphs include clearly labeled error bars for independent experiments and sample sizes. Unless justified, error bars should not be shown for technical replicates.
- if n< 5, the individual data points from each experiment should be plotted and any statistical test employed should be justified
- Source Data should be included to report the data underlying graphs. Please follow the guidelines set out in the author ship guidelines on Data Presentation.

2. Captions

Each figure caption should contain the following information, for each panel where they are relevant:

- a specification of the experimental system investigated (eg cell line, species name).
- the assay(s) and method(s) used to carry out the reported observations and measurements
- an explicit mention of the biological and chemical entity(ies) that are being measured.
- an explicit mention of the biological and chemical entity(ies) that are altered/varied/perturbed in a controlled manner.

- the exact sample size (n) for each experimental group/condition, given as a number, not a range;
- a description of the sample collection allowing the reader to understand whether the samples represent technical or biological replicates (including how many animals, litters, cultures, etc.).
- a statement of how many times the experiment shown was independently replicated in the laboratory.
- definitions of statistical methods and measures:
  - common tests, such as t-test (please specify whether paired vs. unpaired), simple χ2 tests, Wilcoxon and Mann-Whitney tests, can be unambiguously identified by name only, but more complex techniques should be described in the methods section;
  - are tests one-sided or two-sided?
  - are there adjustments for multiple comparisons?
  - exact statistical test results, e.g., P values = x but not P values < x;
  - definition of 'center values' as median or average;
  - definition of error bars as s.d. or s.e.m.

Any descriptions too long for the figure legend should be included in the methods section and/or with the source data.

In the pink boxes below, please ensure that the answers to the following questions are reported in the manuscript itself. Every question should be answered. If the question is not relevant to your research, please write NA (non applicable). We encourage you to include a specific subsection in the methods section for statistics, reagents, animal models and human subjects.

USEFUL LINKS FOR COMPLETING THIS FORM

http://www.antibodypedia.com
http://1degreebio.org
http://www.equator-network.org/reporting-guidelines/improving-bioscience-research-repor
http://grants.nih.gov/grants/olaw/olaw.htm
http://www.mrc.ac.uk/Ourresearch/Ethicsresearchguidance/Useofanimals/index.htm
http://ClinicalTrials.gov
http://www.consort-statement.org
http://www.consort-statement.org/checklists/view/32-consort/66-title
http://www.equator-network.org/reporting-guidelines/reporting-recommendations-for-tumo
http://datadryad.org
http://figshare.com
http://www.ncbi.nlm.nih.gov/gap
http://www.ebi.ac.uk/ega
http://biomodels.net/
http://biomodels.net/miriam/
http://jjj.biochem.sun.ac.za
http://oba.od.nih.gov/biosecurity/biosecurity_documents.html
http://www.selectagents.gov/

B- Statistics and general methods

Please fill out these boxes ↓ (Do not worry if you cannot see all your text once you press return)

1.a. How was the sample size chosen to ensure adequate power to detect a pre-specified effect size?	No sample-size calculation was done in the demonstration of the exploratory data analysis (screening). We performed at least three replicates to show the reproducibility of next generation sequencing data (e.g., by genome-wide correlations and intersection of called peaks).
1.b. For animal studies, include a statement about sample size estimate even if no statistical methods were used.	We used N = 3 for mouse tissues, N = 5 for the Poisson regression model of muscle tissue and N = 1 for the purchased human tissue sample.
2. Describe inclusion/exclusion criteria if samples or animals were excluded from the analysis. Were the criteria pre-established?	No data was excluded from the analysis.
3. Were any steps taken to minimize the effects of subjective bias when allocating animals/samples to treatment (e.g. randomization procedure)? If yes, please describe.	NA
For animal studies, include a statement about randomization even if no randomization was used.	Groups of mice were randomly allocated into ChIL/RNA-seq.
4.a. Were any steps taken to minimize the effects of subjective bias during group allocation or/and when assessing results (e.g. blinding of the investigator)? If yes please describe.	Investigators were blinded to the group allocation up to the ChIL/RNA-seq steps.
4.b. For animal studies, include a statement about blinding even if no blinding was done	NA
5. For every figure, are statistical tests justified as appropriate?	Statistical tests were performed in all figure where it is required.
Do the data meet the assumptions of the tests (e.g., normal distribution)? Describe any methods used to assess it.	The overdispersions were not observed for the count data of ChIL-seq by confirming the mean-dispersion plots.
Is there an estimate of variation within each group of data?	Yes

Is the variance similar between the groups that are being statistically compared?	NA

C- Reagents

6. To show that antibodies were profiled for use in the system under study (assay and species), provide a citation, catalog number and/or clone number, supplementary information or reference to an antibody validation profile. e.g., Antibodypedia (see link list at top right), 1DegreeBio (see link list at top right).	The antibodies used in this study were listed in Structured Method.
7. Identify the source of cell lines and report if they were recently authenticated (e.g., by STR profiling) and tested for mycoplasma contamination.	NA

* for all hyperlinks, please see the table at the top right of the document

D- Animal Models

8. Report species, strain, gender, age of animals and genetic modification status where applicable. Please detail housing and husbandry conditions and the source of animals.	Eight-week-old C57BL/6N mice (male/female) were purchased from Japan SLC,Inc.
9. For experiments involving live vertebrates, include a statement of compliance with ethical regulations and identify the committee(s) approving the experiments.	All animal procedures were conducted in accordance with the Guidelines for the Care and Use of Laboratory Animals and were approved by the Institutional Animal Care and Use Committee (IACUC) at Kyushu University.
10. We recommend consulting the ARRIVE guidelines (see link list at top right) (PLoS Biol. 8(6), e1000412, 2010) to ensure that other relevant aspects of animal studies are adequately reported. See author guidelines, under 'Reporting Guidelines'. See also: NIH (see link list at top right) and MRC (see link list at top right) recommendations. Please confirm compliance.	Confirmed

E- Human Subjects

11. Identify the committee(s) approving the study protocol.	The study protocol was approved (number: 579-04) by the institutional review board for clinical research (IRB) of Kyushu University.
12. Include a statement confirming that informed consent was obtained from all subjects and that the experiments conformed to the principles set out in the WMA Declaration of Helsinki and the Department of Health and Human Services Belmont Report.	NA
13. For publication of patient photos, include a statement confirming that consent to publish was obtained.	NA
14. Report any restrictions on the availability (and/or on the use) of human data or samples.	ChIL and RNA-seq data of purchaced tissue samples are publicly available at NCBI GEO.
15. Report the clinical trial registration number (at ClinicalTrials.gov or equivalent), where applicable.	NA
16. For phase II and III randomized controlled trials, please refer to the CONSORT flow diagram (see link list at top right) and submit the CONSORT checklist (see link list at top right) with your submission. See author guidelines, under 'Reporting Guidelines'. Please confirm you have submitted this list.	NA
17. For tumor marker prognostic studies, we recommend that you follow the REMARK reporting guidelines (see link list at top right). See author guidelines, under 'Reporting Guidelines'. Please confirm you have followed these guidelines.	NA

F- Data Accessibility

18: Provide a "Data Availability" section at the end of the Materials & Methods, listing the accession codes for data generated in this study and deposited in a public database (e.g. RNA-Seq data: Gene Expression Omnibus GSE39462, Proteomics data: PRIDE PXD000208 etc.) Please refer to our author guidelines for 'Data Deposition'.<br><br>Data deposition in a public repository is mandatory for:<br>a. Protein, DNA and RNA sequences<br>b. Macromolecular structures<br>c. Crystallographic data for small molecules<br>d. Functional genomics data<br>e. Proteomics and molecular interactions	We described Data Availability section as instructed.
19. Deposition is strongly recommended for any datasets that are central and integral to the study; please consider the journal's data policy. If no structured public repository exists for a given data type, we encourage the provision of datasets in the manuscript as a Supplementary Document (see author guidelines under 'Expanded View' or in unstructured repositories such as Dryad (see link list at top right) or Figshare (see link list at top right).	The datasets and computer code produced in this study are available in the following: NCBI GEO (https://www.ncbi.nlm.nih.gov/geo/query/acc.cgi?acc=GSE159024), computer codes and sample dataset (https://github.com/kazumits/tissueChIL).
20. Access to human clinical and genomic datasets should be provided with as few restrictions as possible while respecting ethical obligations to the patients and relevant medical and legal issues. If practically possible and compatible with the individual consent agreement used in the study, such data should be deposited in one of the major public access-controlled repositories such as dbGAP (see link list at top right) or EGA (see link list at top right).	NA
21. Computational models that are central and integral to a study should be shared without restrictions and provided in a machine-readable form. The relevant accession numbers or links should be provided. When possible, standardized format (SBML, CellML) should be used instead of scripts (e.g. MATLAB). Authors are strongly encouraged to follow the MIRIAM guidelines (see link list at top right) and deposit their model in a public database such as Biomodels (see link list at top right) or JWS Online (see link list at top right). If computer source code is provided with the paper, it should be deposited in a public repository or included in supplementary information.	Computational codes to reproduce our results are publicly available at Github.

G- Dual use research of concern

22. Could your study fall under dual use research restrictions? Please check biosecurity documents (see link list at top right) and list of select agents and toxins (APHIS/CDC) (see link list at top right). According to our biosecurity guidelines, provide a statement only if it could.	NA
